# RBM20 variants disrupt Ca^2+^ handling and metabolism in dilated and non-compaction cardiomyopathy stem cell models

**DOI:** 10.1038/s41392-026-02838-7

**Published:** 2026-07-14

**Authors:** Sabine Rebs, Farbod Sedaghat-Hamedani, Elham Kayvanpour, Hanna Eberl, Branimir Berečić, Jan Dudek, Nicole Wagensohner, Aylin Seedorf, Julia K. Unsöld, Daniela Hübscher, Christoph Reich, Teresa Klein, Sören Doose, Viacheslav O. Nikolaev, Tjark Buchwald, Ahmed Wagdi, Michael Kohlhaas, Nataliya Dybkova, Patricia Morais Costa, Kaomei Guan, Gerd Hasenfuss, Eva A. Rog-Zielinska, Samuel Sossalla, Markus Sauer, Christoph Maack, Malte Tiburcy, Benjamin Meder, Katrin Streckfuss-Bömeke

**Affiliations:** 1 Institute of Pharmacology and Toxicology, University of Würzburg, Würzburg, Germany; 2 Clinic for Cardiology and Pneumology, University Medical Center Göttingen and DZHK (German Center for Cardiovascular Research), partner Site Göttingen, Göttingen, Germany; 3 Institute for Cardiomyopathies Heidelberg, Department of Cardiology, Angiology and Pneumology, University Hospital Heidelberg, and DZHK (German Center for Cardiovascular Research), partner Site Heidelberg, Heidelberg, Germany; 4 Rehaklinik Königstuhl, Heidelberg, Germany; 5 Institute of Pharmacology and Toxicology, University Medical Center Göttingen and DZHK (German Center for Cardiovascular Research), partner Site Lower Saxony, Göttingen, Germany; 6 Comprehensive Heart Failure Center (CHFC), University Clinic Würzburg, Würzburg, Germany; 7 Department of Biotechnology and Biophysics, Biocenter, University of Würzburg, Am Hubland, Würzburg, Germany; 8 Institute for Experimental Cardiovascular Research, University Medical Center Hamburg-Eppendorf, Hamburg, and DZHK (German Center for Cardiovascular Research) partner site Hamburg, Hamburg, Germany; 9 Cluster of Excellence Multiscale Bioimaging: from Molecular Machines to Networks of Excitable Cells (MBExC 2067), University of Göttingen, Göttingen, Germany; 10 Institute for Cardiovascular Physiology, University Medical Center Göttingen, Göttingen, Germany; 11 Institute for Experimental Cardiovascular Medicine, University Heart Centre, Medical Centre - University of Freiburg, and Faculty of Medicine, University of Freiburg, Freiburg, Germany; 12 Institute of Pharmacology and Toxicology, Technical University Dresden, Dresden, Germany; 13 Department of Cardiology, Campus Kerckhoff, Bad Nauheim, Germany; 14 Cardio-Pulmonary Institute (CPI), Giessen, Germany; 15 Medical Clinic I, Cardiology and Angiology, Justus-Liebig-University, Giessen, Germany; 16 Medical Clinic and Polyclinic I, University Clinic Würzburg, Würzburg, Germany; 17 Stanford Genome Technology Center, Department of Genetics, Stanford, CA USA; 18 Present Address: Cluster of Excellence Multiscale Bioimaging: from Molecular Machines to Networks of Excitable Cells (MBExC 2067), University of Göttingen, Göttingen, Germany; 19 Present Address: Clinic for Cardiology and Pneumology, University Medical Center Göttingen and DZHK (German Center for Cardiovascular Research), partner Site Göttingen, Göttingen, Germany

**Keywords:** Cardiology, Translational research

## Abstract

Mutations in the splice-regulator RBM20 cause heart failure with reduced ejection fraction (HFrEF), typically manifesting as dilated cardiomyopathy (DCM). Mutations at position 634 in the RS-domain cause DCM *with* (R634L) or *without* (R634W) left ventricular non-compaction (LVNC). However, the mechanisms underlying phenotype variability and personalized therapy beyond HFrEF remain unclear. We generated induced pluripotent stem cell-derived cardiomyocytes (iPSC-CM), 3D-cardiospheres and engineered myocardial tissues from patients with RBM20 mutations R634L (LVNC) or R634W (DCM). Using CRISPR/Cas9, we created isogenic rescue and mutation-insertion lines, identifying RBM20 mis-localization, splicing errors in *TTN* and *RYR2*, and sarcomere irregularities in both. DCM-CM showed increased resting Ca^2+^ leak and reduced Ca^2+^ transient amplitude, typical of HFrEF, and spatial disorganization of sarcoplasmic reticulum and mitochondria. In contrast, LVNC-CM exhibited elevated Ca^2+^ transient amplitude with faster kinetics, driven by elevated cAMP and mis-spliced, hyperactive CAMK2D, leading to PLN-hyperphosphorylation and increased metabolic respiration. Further, LVNC showed desmosomal derangement potentially from mis-splicing of *Junction plakoglobin* and reduced 3D cardiosphere compaction. Despite distinct mechanisms, contractile force was reduced in both. Isogenic controls confirm mutation causality. Drug intervention with verapamil partially improved selected abnormal Ca^2+^ handling and contractile phenotypes in LVNC- and DCM-CM, whereas the CAMK2D inhibitor AIP improved systolic Ca^2+^ handling predominantly in LVNC-CM. In conclusion, different amino acid substitutions at the same RBM20-residue induce opposing Ca^2+^-handling and structural phenotypes. While DCM features impaired Ca^2+^ handling, LVNC shows defective cell-cell coupling and activated Ca^2+^ handling and metabolism, yet insufficient to compensate for organ-level dysfunction. This supports personalized pharmacological therapies in early HF, and potential CRISPR/Cas9 repair for RBM20 cardiomyopathy.

## Introduction

Heart failure (HF) is among the most common causes of hospitalization in Europe and the United States and is associated with high morbidity and mortality. Among patients with HF with reduced ejection fraction (HFrEF), dilated cardiomyopathy (DCM) contributes to 30-40% of cases. DCM is characterized by left ventricular (LV) dilation, thinning, and contractile dysfunction.^1,2^ In contrast, LV non-compaction cardiomyopathy (LVNC) is rarer and characterized by hyper-trabeculation of the myocardium showing deep recesses within the ventricular muscle, and can additionally be accompanied by dilation of the chambers.^2^ LVNC is either the result of developmental defects with incomplete myocardial compaction, which generally occur during the 5th to 8th week of embryogenesis, or later in life due to genetic defects or afterload increases.^3^ Despite distinct clinical features, the recent European guidelines classify non-compaction or hyper-trabeculation as an additional phenotypic trait of DCM, rather than a distinct cardiomyopathy.^2^ Numerous genetic studies have revealed overlapping gene loci for LVNC and DCM, including many that affect sarcomeric or structural proteins, some having a severely impaired prognosis.^4–6^ Hence, genetic testing has a pivotal role in diagnosis and risk stratification in both patient groups.^7^

Across large European and North American DCM cohorts, pathogenic RNA Binding Motif Protein 20 (RBM20) variants account for approximately 2–3% of clinically diagnosed DCM cases.^8,9^ RBM20 cardiomyopathy shows high and age-dependent penetrance: in the largest familial cohort, 66% of genotype-positive relatives developed HFrEF, and 30% experienced a life-threatening arrhythmic or HF event, with a mean age at first clinical presentation typically in the third to fourth decade.^10,11^ We previously associated the distinct RBM20-variant R634L with LVNC, which has not been observed in pure DCM cases.^6^ RBM20 is a major cardiac splicing factor with more than 30 target mRNAs linked to cardiac muscle function, such as *RYR2*, *TTN*, or *CAMK2D.*^12^ The mutational hotspot within the RBM20 gene is located at the highly conserved RS-domain (p.633-638 - PRSRSP) in exon 9. This RS-domain mediates protein-protein interactions with spliceosomal components, promoting assembly of the spliceosome on its target pre-mRNA, such as *TTN.*^13^ Additionally, the RS-stretch functions as a nuclear localization signal to import RBM20 protein back into the nucleus, where it can function as a splice factor.^14^ Whether defective RBM20 splicing function is caused by mutation-dependent mis-splicing within the nucleus or loss of import of RBM20 into the nucleus is still an active area of research.^15^ In this hotspot, 16 mutations have been described as pathogenic variants, including the DCM-variant p.R634W and the LVNC-variant p.R634L.^15^ Recent studies expanded the pathogenic scope of RBM20 mutations to hypertrophic cardiomyopathy.^16,17^ There is also evidence that mutations outside the RS-domain are causative for inherited DCM.^18^ However, if and how mutations in the same gene and even the same amino acid position can determine different cardiomyopathies needs a deeper mechanistic understanding.

The generation of induced pluripotent stem cells (iPSC) derived from patients, as well as the introduction of mutations by CRISPR/Cas9 genome editing, is an invaluable tool of precision medicine.^19,20^ The successful differentiation of functional iPSC-cardiomyocytes (CM) was an important step in cardiac research, and many studies have hence demonstrated that patient-specific iPSC-CM recapitulate disease-related cardiac phenotypes in vitro.^21–23^ Although RBM20 research has spanned the model organisms of mice, rats, and pigs, the animal models often represent an RBM20 knockout model or a homozygous variant rather than the clinically relevant heterozygous missense mutations or have a species-specific limitation of animal physiology.^12,24,25^ Of note, very rare heterozygous loss of function variants were recently reported in the context of arrhythmia.^26^ Some studies used patient-specific-iPSC for analysis of RBM20 mutations (i.e., p.S635A and R636S),^22,27^ while others have introduced a patient-relevant mutation into a healthy iPSC line (P633L, R634Q, R636S).^28,29^ In general, the pathological effects of RBM20 mutations are attributed to erroneous splicing of target genes and/or RBM20 protein accumulation in the cytoplasm. However, it is currently unclear which one of these two is the predominant driver for the development of cardiomyopathies. In general, variants in RBM20 typically deteriorate excitation-contraction coupling through alterations in sarcomeric structure and function or cytosolic Ca^2+^ handling, commonly resulting in reduced contractile performance.^15^

Here, we analyzed two genetic variants in RBM20 at the very same amino acid position p.R634, which were linked to either a LVNC (p.R634L) or DCM phenotype (p.R634W) and investigated the pathogenesis of these mutations on the molecular, cellular, and functional level. We used iPSC-CM generated from respective cardiac patients and compared them to isogenic rescue and insertion lines generated by CRISPR/Cas9 genome editing. A comparable analysis of both RBM20 variants using patient-specific stem cell models allows for an in-depth analysis of RBM20-linked LVNC and DCM. Furthermore, this study expands the scope of RBM20-pathology into metabolic and structural alterations and provides a rationale for a more personalized approach with Ca^2+^ channel blockers, especially in early-stage HF.

## Results

### Patient-specific iPSC-CM from two different cardiomyopathy families with distinctive RBM20 mutations

**Fig. 1:**
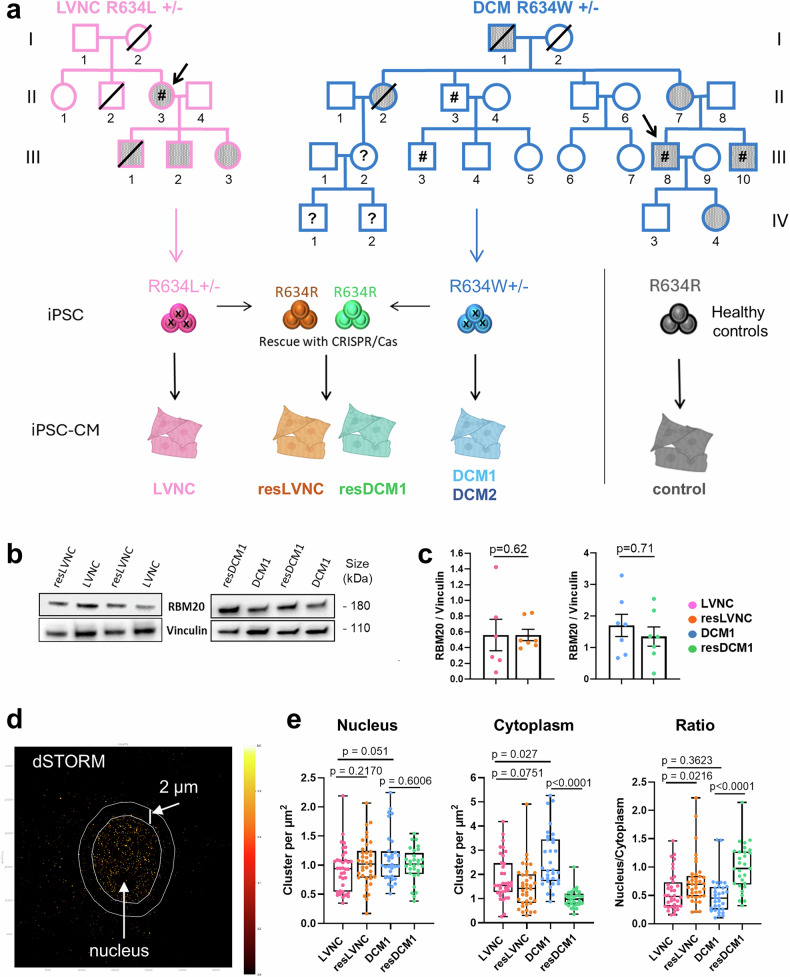
Generation of iPSC-CM of LVNC- and DCM-affected family members with RBM20 mutations. **a** Pedigree of LVNC- and DCM-affected families carrying a RBM20 mutation at amino acid position R634. Filled symbols labeled disease-affected individuals. Symbols marked with a (#) are individuals who donated somatic material used for reprogramming into iPSC. Crossed symbols mean the individual is deceased. The arrow marks the patient of which isogenic control lines were derived. The control category (grey/black) comprises iPSC-CM, which was derived from healthy DCM family members and unrelated healthy individuals already published by our group. **b** Example of Western blot membranes for RBM20 and Vinculin for in LVNC-, resLVNC-, DCM1- and resDCM1-CM. **c** Quantification of RBM20 protein levels normalized to Vinculin used as loading control. Data is presented as bar graphs with mean ± SEM, where each dot represents one cardiac differentiation. *P*-values by Mann-Whitney test. **d** Representative RBM20 *d*STORM image from DCM1-CM. RBM20 was labelled with a fluorescently-labeled antibody against RBM20. Two ROIs per cell, nucleus, and cytoplasm, were analyzed with LOCAN to determine the RBM20 protein amount that is distributed between nucleus and cytoplasm. **e** Quantification of RBM20 cluster densities, calculated for the nucleus, the cytoplasm, and the ratio of nucleus divided by cytoplasm value for each cell. Data is presented in box plots showing all data points. Each dot in the box plot represents one cell with [Number of differentiations/analyzed cells] for LVNC [2/36], resLVNC [2/38], DCM1 [2/32], and resDCM1 [2/29]. *P*-values calculated by Mann-Whitney test (LVNC vs resLVNC; LVNC vs DCM1 and DCM1 vs resDCM1]

To study the relationship between RBM20 variants in the context of two different cardiomyopathy traits, we recruited one patient diagnosed with LVNC (DCM with non-compaction trait) and two patients with DCM (without non-compaction), each from different families (Fig. 1a), for the reprogramming of somatic material into iPSC lines. The LVNC index patient (LVNC pedigree II.3) harbors the RBM20 variant p.R634L and was diagnosed with a severe form of LVNC and HF.^6^ Diagnosed at age 39, she had a LVEF of only 20%, which led to the implantation of an implantable cardioverter-defibrillator (ICD). Her three children are also affected by heart disease: III.1 died at a young age due to tetralogy of Fallot. The other son and daughter both inherited the RBM20 variant p.R634L with varying severities. Her daughter (III.3) exhibits LV hyper-trabeculation but is symptom-free with a normal LVEF (56%) at age 35. The son (III.2) underwent heart transplantation at age 17 due to severe LVNC and HF. The RBM20 DCM-linked variant p.R634W was initially identified in cohort screenings.^30^ Two brothers (DCM pedigree III.8, III.10) characterized with DCM and reduced LVEF (40% and 30%, respectively) harbor this RBM20 variant p.R634W. Additionally, the daughter (IV.4) of DCM1 (III.8) inherited the p.R634W variant and also suffers from DCM (Fig. 1a). The data from the latest clinical visit for the three patients is provided in Supplementary Table 1. The two DCM patients are presented separately by different colors or symbols. To evaluate the pathogenic potential of p.R634L and p.R634W mutations, patient-specific RBM20 iPSC were generated. CRISPR/Cas9 was applied to produce isogenic rescue iPSC lines, termed resLVNC and resDCM1 (Fig. 1a). In addition to healthy controls previously described by us,^21^ two healthy family members from the DCM family (II.3 and III.3) donated somatic material (Fig. 1a). The generation and characterization of DCM-iPSC lines from patient III.8 (here termed DCM1) and the corresponding isogenic control line (here termed resDCM1) was recently published by our groups.^31^ The remaining iPSC lines generated in this study exhibit full pluripotency and spontaneous in vitro differentiation capacity (Supplementary Fig. 1). Furthermore, the patient lines show genetic integrity after gene editing (Supplementary Fig. 2a) and harbor the expected nucleotide sequences (Supplementary Fig. 2b). All iPSC lines successfully differentiated into beating cardiomyocytes (iPSC-CM) with an efficiency of 80 – 97% cardiac troponin T (cTNT)-positive cells (Supplementary Fig. 2c) and high expression of the ventricular marker *MYL2* concomitant with low expression of the atrial marker *NR2F2* (Supplementary Fig. 2d). Furthermore, the maturation of iPSC-CM is a critical step toward generating physiologically relevant cardiac models. In our study, we employed long-term culture to enhance the maturation status for all cardiac differentiations used. Additionally, a 3D spheroid culture in maturation medium was selectively used for assays requiring advanced maturation to complement the long-term culture model.^32^ Taken together, the generated iPSC-lines provide a suitable model for the study of RBM20-linked cardiomyopathies.

### RBM20 mutations affect protein localization and RBM20-dependent splice targets

The RBM20 mutations have no effect on RBM20 expression levels between LVNC- and DCM-CM (Fig. 1b/c). However, since the mutations are located in RBM20´s RS-domain, which is essential for nuclear localization, we analyzed the impact of both variants on the localization of the RBM20 protein. Immunofluorescence stainings of RBM20 demonstrated that for both LVNC- and DCM-CM, RBM20 signals are detected in the sarcoplasm and the nucleus, whereas for healthy and isogenic iPSC-CM the RBM20 signal is distinctly limited to the nucleus (Supplementary Fig. 3a). To quantify this observation super-resolution imaging of RBM20 was performed using single-molecule localization microscopy by *direct* stochastic optical reconstruction microscopy (*d*STORM)^33,34^ (Fig. 1d). Employing customized software LOCAN^35^ with a DBSCAN^36^ clustering algorithm (Fig. 1d), we observed that the ratio of RBM20 densities (nucleus to sarcoplasm) in LVNC- and DCM-CM is significantly reduced compared to their isogenic rescue lines. (Fig. 1e). Notably, this ratio is driven by higher values for cytoplasmatic RBM20, and DCM-CM shows significantly more accumulation than LVNC-CM. Taken together, these results indicate that RBM20 protein aberrantly accumulates in the sarcoplasm in the presence of a missense mutation at position p.634, with the DCM-associated variant p.R634W showing a more pronounced effect.

**Fig. 2:**
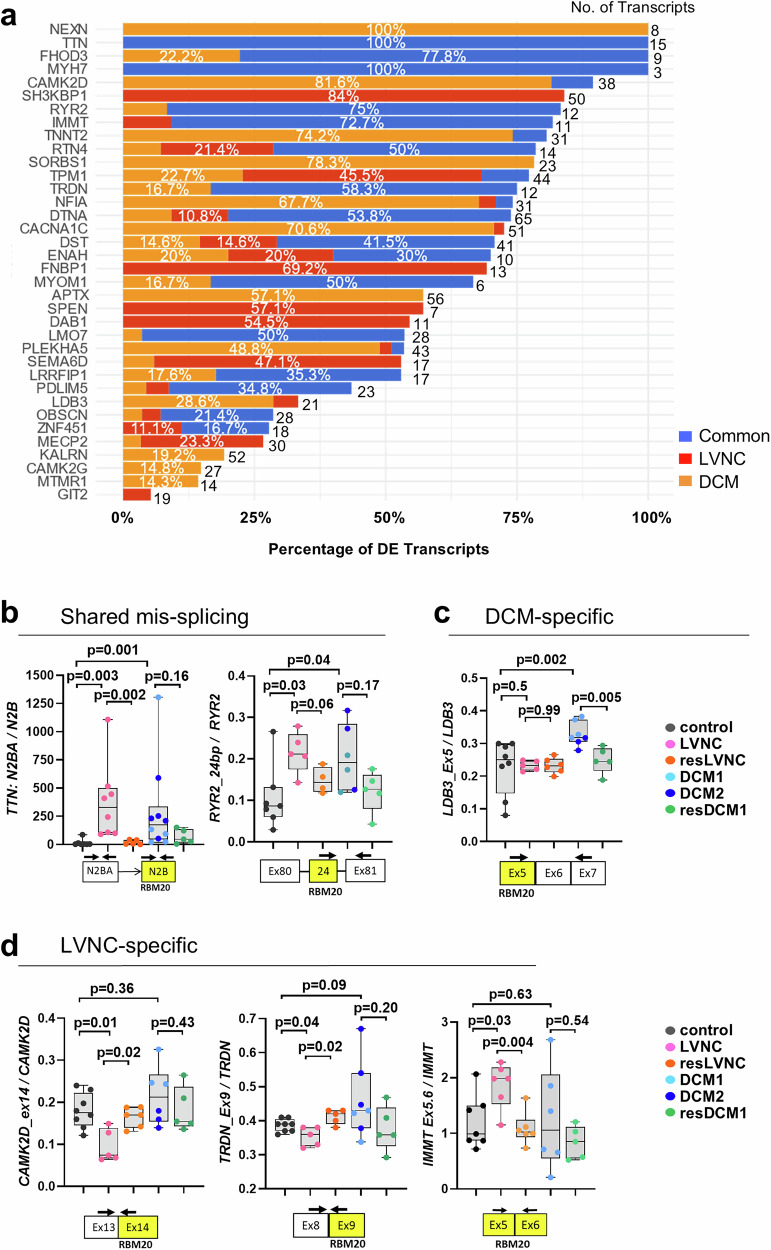
RBM20-dependent splice defects in LVNC- and DCM-CM. **a** The stacked bar chart depicts the differentially expressed exon usage percentage across a selection of RBM20 target genes. For each gene, the total number of unique transcripts is shown as a percentage of three categories: common differentially expressed (DE) exon usage for DCM and LVNC (blue), DE exon usage unique to DCM (orange), and DE exon usage unique to LVNC (red). The y-axis lists RBM20 target genes. The numbers on the bars indicate the actual percentages for segments greater than 10%. In addition, the total number of unique transcripts per gene is shown to the right of the bars. **b**–**d** QPCR analysis of RBM20 splice targets. Primers were designed to target the exon of interest, and expression levels were normalized to the total gene expression by using primers directed against a constitutively expressed exon. Data is shown as box plots, whereas every dot represents one cardiac differentiation experiment. P-values were determined by Mann-Whitney test control vs. patient line (LVNC, DCM1/DCM2) and patient line vs. rescue line (resLVNC, resDCM1). **b** Shared mis-splicing events in LVNC- and DCM-CM. The primers were designed against the N2BA and N2B domain of *TTN*, and an intronic 24 bp insertion of *RYR2*. **c** Differential mis-splicing for exon 5 in *LDB3* in DCM-CM. **d** Differential mis-splicing for exon 14 in *CAMK2D* and exon 9 in *TRDN* for LVNC-CM

Besides localization, we assessed the effects of RBM20 mutations p.R634L and p.R634W on RBM20-dependent splice targets. RBM20 splice targets have been well defined since the first description by Guo and colleagues in 2012^12^ and can be categorized functionally into Ca^2+^ handling genes, ion channels, and structural proteins/genes. To gain further mechanistic insight, gene expression and splice isoform profiling were performed. Principal component analysis (PCA) plot shows clustering of disease versus rescue lines, but some overlap between DCM and LVNC (Supplementary Fig. 3b). We identified 1874 genes that are differentially expressed between resLVNC and LVNC, with 718 downregulated and 1156 upregulated (adjusted *p*-value ≤ 0.05, abs (fold change) > 1.5) and 3377 genes between resDCM1 and DCM with 1782 downregulated and 1595 upregulated (adjusted *p*-value ≤ 0.05, abs (fold change) > 1.5). Only 24 genes were differentially expressed (adjusted *p*-value ≤ 0.05, abs (fold change) > 1.5) between LVNC and DCM, whereas 14 of these genes account for y-linked genes since DCM lines are derived from a male patient (Supplementary Fig. 3c, Supplementary Table 7). Notably, gene expression of RBM20 splice targets is scarcely affected in LVNC- and DCM-CM (Supplementary Fig. 3d). Furthermore, gene ontology (GO) analysis of the top 100 differentially expressed genes for resLVNC vs LVNC and for resDCM1 vs DCM1 does show only a few cardiac relevant GO term hits (Supplementary Fig. 4a, b and Supplementary Tables 8, 9). Taken together, this suggests that gene expression differences are unlikely to drive the disease phenotypes observed for LVNC- and DCM-CM. However, analyzing the gene expression data for gene splice isoform expression, we observed that known RBM20 splice targets are spliced differently depending on the RBM20 variant. Intriguingly, only two of the 36 RBM20 splice targets analyzed are identically spliced between LVNC- and DCM-CM, which are *MYH7* and *TTN* (Fig. 2a), while others are distinct for LVNC, such as *SH3KPB1* or *NEXN* for DCM-CM (Supplementary Fig. 5a). In conclusion, the profiling indicated that differential gene isoform expression of RBM20 target genes is a more dominant disease driver for these RBM20-dependent cardiomyopathies instead of the change in the overall gene expression.

**Fig. 3:**
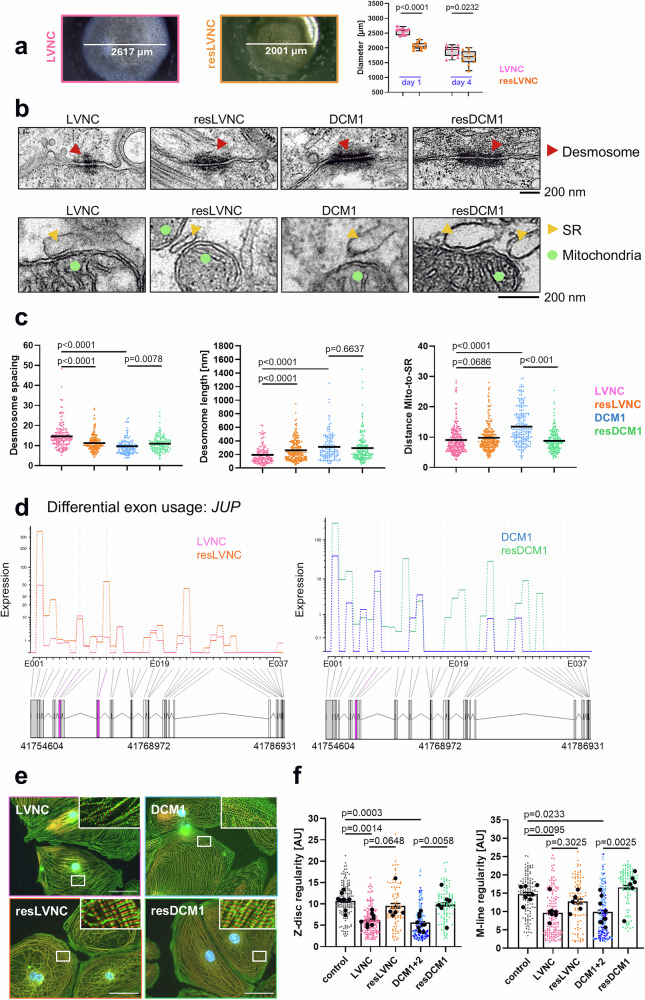
RBM20 ultrastructural and sarcomeric defects in LVNC- and DCM-CM. **a** Representative brightfield images of 3D spheroid culture of LVNC- and resLVNC spheroids. Quantification of diameter of acquired spheroid images for single independent spheroids: LVNC [10] and resLVNC.[10] Data is shown as box plots, whereas every dot represents one independent spheroid. Reported *p*-values were calculated by Mann–Whitney test LVNC vs resLVNC for day 1 and day 4, respectively. The LVNC-spheroids exhibit a greater diameter at d1 and d4 after spheroid casting. **b** Representative transmission electron micrographs revealed smaller desomosome connections in LVNC-spheroids and increased SR-mitochondrial distance in DCM1-spheroids. Ultrastructural features are marked in red (desmosome), yellow (SR: sarcoplasmic reticulum), and green (mitochondria). SR = sarcoplasmatic reticulum. **c** Quantification of desmosome length, desmosome spacing and distance of mitochondria to SR in independent regions within imaged spheroids. Data is shown as scatter blot with mean, where each dot represents an independent region for *n* = 2 for each cell line. [Number of regions analyzed for desmosome length, spacing and SR-mito distance] for the LVNC [159, 140 255]-, resLVNC [170,211,258]-, DCM1 [130, 137,198]-, and resDCM1 [122, 166, 173]-spheroids. *P*-values were calculated by Kruskal–Wallis test with Dunn´s multiple comparisons for LVNC vs resLVNC, LVNC vs DCM1, and DCM1 vs resDCM1. **d** Splice graphs with exonic bin usage of *JUP* (Junction Plakoglobin) of LVNC and DCM-CM derived from the mRNA sequencing data set. LVNC shows more hits in differential exon usage in the desmosomal gene *JUP* than DCM. Graphs represent the differential exon expression profiles. The *y*-axis represents normalized read counts of exons (exon usage), and the *x*-axis shows individual exons within a gene. The lower panels show the gene structure, with the bars below the *x*-axis representing exons, and the lines between the bars representing introns. The numbers at the bottom are genomic locations of the gene. Purple bars mark exons with significantly altered usage (FDR-adjusted *p*-value < 0.05) in the DEX analysis of isogenic versus patient cell lines. **e** Representative immunofluorescence stainings of LVNC-, resLVNC-, DCM1-, and resDCM1-CM. Visualization of sarcomeric Z-disc by staining against α-actinin (green) and the M-line by TTN (M8/M9 antibody) (red). LVNC- and DCM-CM show disarrayed sarcomeric structures. For visualization purposes the brightness and contrast have been enhanced in these exemplary images. Scale bars: 50 µm. **f** Quantifying Z-disc (α-actinin) and M-line (TTN) sarcomeric regularity using Fast Fourier transformation and peak amplitude of the first-order peak as a measure for regularity. Data is presented as bar graphs with mean ± SEM showing all data points, whereas the large black dots represent the mean of an individual differentiation. Multiple pictures were analyzed [number of differentiations/analyzed pictures] regarding Z-disc regularity for control [8/146], LVNC [9/164], resLVNC [5/86], DCM1+ 2 [9/175], resDCM1 [6/105], and regarding M-line regularity for control [7/128], LVNC [9/164], resLVNC [5/86], DCM1+2 [9/175], and resDCM1 [6/105]. *P*-values were calculated with nested 1way-ANOVA with Sidak´s multiple comparisons for the selected groups: control vs LVNC, control vs DCM1+ 2, LVNC vs resLVNC, DCM1+2 vs resDCM1

Next, we selected splice targets that were also identified in *Rbm20* knockout rats^12^ and translated them to the corresponding human loci, allowing us to assess in our iPSC-based system which isoforms are expressed, and which rat exons correspond to human gene regions.^37^ Splicing of titin (*TTN*) and ryanodine receptor 2 (*RYR2*) demonstrated RBM20-dependent mis-splicing in LVNC- and DCM-CM (Fig. 2a). The *TTN* mRNA showed transcripts with an increased ratio of longer (N2BA) to shorter (N2B) isoform due to decreased N2B levels when compared to control-CM, although this effect was not rescued for resDCM1 (Fig. 2b and Supplementary Fig. 3e). The shorter N2B isoform is increased during maturation and annotated as the adult *TTN* form. Expression of *RYR2*-mRNA that includes a cryptic 24 bp insertion from intron 80-81 was also increased for LVNC- and DCM-CM compared to control-CM (Fig. 2b). By contrast, transcripts with included exon 5 in LIM domain binding 3 (*LDB3)* were only increased in DCM-CM compared to control- and resDCM1-CM (Fig. 2c). Furthermore, the inclusion of exon 9 in triadin (*TRDN)* as well as inclusion of a nuclear localization signal (NLS) in exon 14 of the Ca^2+^/calmodulin-dependent protein kinase type 2 delta (*CAMK2D)* and exon5/6 of inner mitochondrial membrane protein (*IMMT)* were only affected in LVNC-CM, but not in DCM-CM compared to control- and resLVNC-CM (Fig. 2d). In conclusion, these data suggest that splicing of RBM20-targets was affected differentially depending on the missense mutation R634L or W, while mis-localization of the RBM20-protein was similar for both missense variants. Since splicing is highly dependent on maturation status, we analyzed two important RBM20 splice targets, *TTN* and *CAMK2D*, again in 3D spheroid cultured iPSC-CM. This recently published culture approach showed beneficial maturation effects, especially compared to 2D long-term culture.^32^ Notably, LVNC exhibits a structural component (trabecular recesses in the left ventricle) that cannot be adequately captured in 2D cultures, but becomes evident in 3D cardiac spheroid culture, where LVNC-spheroids display less compaction with increased diameters compared to resLVNC-spheroids (Fig. 3a). The splice defects in *TTN* and *CAMK2D* become more clearly detectable (Supplementary Fig. 5b) with 3D spheroid culture, evident by the significant rescue in the isogenic line resDCM1 for *TTN-N2B*, which was not observed before in the 2D long-term cultured set (Fig. 2b).

### Ultrastructural and sarcomeric impairment in LVNC- and DCM-CM is caused by the distinct RBM20 mutations

To gain further insight into the less compact structure of LVNC-spheroids, we used transmission electron microscopy (TEM) to investigate the sub-cellular ultrastructure. Quantification of desmosome architecture revealed decreased length and increased spacing between the single CM in LVNC-spheroids compared to DCM-spheroids, which was attenuated by rescue of the RBM20 mutation p.R634L. In contrast, DCM1-spheroids exhibited a longer distance between SR and mitochondria when compared to resDCM1- and LVNC-spheroids (Fig. 3b, c). We examined RBM20 mutation-dependent differences in exon expression (DEEs) between LVNC and DCM within desmosomal genes and identified Junction plakoglobin (*JUP*) as a key candidate. LVNC displayed a greater number of differential exon usage events in *JUP* compared to DCM (Fig. 3d). Taken together, the two distinct RBM20 mutations influence different aspects of CM ultrastructure.

A sarcomere is the smallest contractile unit of a CM, and the amount and regularity of the sarcomeres determines the contractile properties of cardiac cells.^38^ Since both RBM20 mutations R634L (LVNC) and R634W (DCM) alter the *TTN* isoform, we focused on the sarcomeric organization pattern in iPSC-CM. To this end, double staining was used to visualize the sarcomeric Z-disc with α-actinin and the M-line with TTN M8/M9 antibodies (Fig. 3e), followed by quantification of sarcomeric regularity using fast Fourier Transformation (FFT). We observed significant impairment in sarcomeric regularity for the Z-disc and M-line in LVNC-CM as well as DCM-CM compared to control-CM (Fig. 3f). Isogenic rescue iPSC-CM showed a regular sarcomeric structure comparable to the control cells, demonstrating the importance of wt-RBM20 for sarcomeric regularity. As sarcomeric irregularity is a common trait for RBM20 mutations, we cannot distinctly pinpoint whether this is due to *TTN* mis-splicing or RBM20 mis-localization as these molecular aberrations are shared between LVNC- and DCM-CM.

### Dysfunctional Ca^2+^ homeostasis and contractility for LVNC- and DCM-CM

**Fig. 4:**
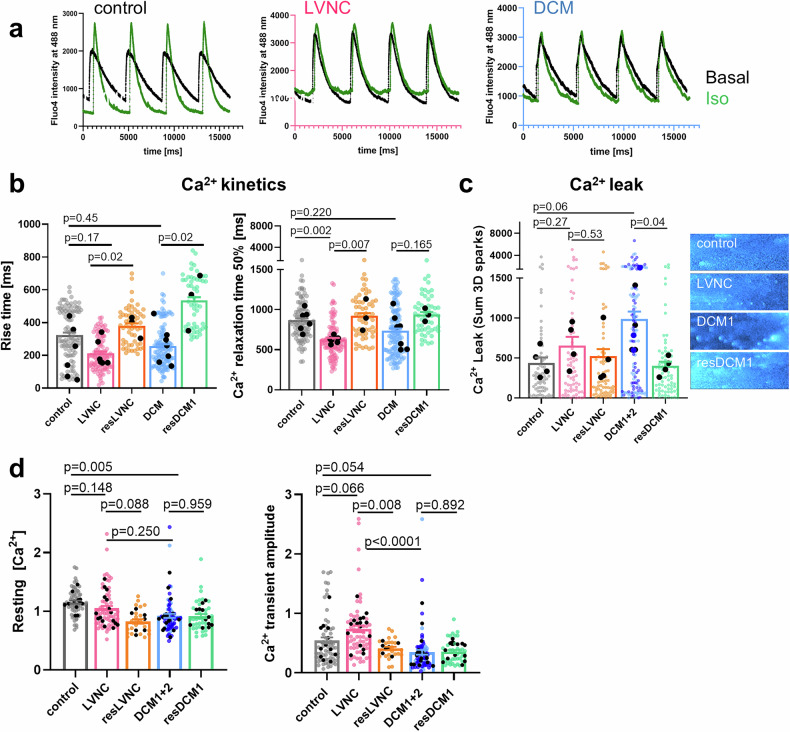
LVNC- and DCM-CM show differential Ca^2+^ handling pathologies. **a** Exemplary single-cell Ca^2+^ traces from iPSC-CM of control, LVNC, and DCM at basal (black) and Isoprenaline (Iso) stimulation (1 µM) measured with Fluo-4-AM at 0.25 Hz pacing. **b** Ca^2+^ transient rise time (time to peak) and relaxation time in ms in untreated iPSC-CM using Fluo-4-AM at 0.25 Hz pacing. [number of differentiations/analyzed cells] for control [6/94], LVNC [6/101], resLVNC [3/55], DCM [7/126], and resDCM1 [3/51]. Data is represented as bar graph with mean + SEM with each colored dot represents one measured cell with the large black dots depicting the mean of one cardiac differentiation. *P*-values were calculated with nested *t*-test: control vs LVNC, control vs DCM1, LVNC vs resLVNC, and DCM1 vs resDCM1. **c** Increased Ca^2+^ leakage in DCM-CM. Quantification of Ca^2+^ leak (left) and representative original confocal line scans showing SR Ca^2+^ sparks in iPSC-CM using the ImageJ sparkmaster plugin (right). Data is shown in bar graph with mean + SEM [number of differentiations/analyzed cells] for control [4/70], LVNC [4/67], resLVNC [4/70], DCM1 [4/63]/ DCM2 [3/55], and resDCM1 [4/71]. Each colored dot represents one measured cell with the black dots depicting the mean of one cardiac differentiation. *P*-values were calculated with nested *t*-test: control vs LVNC, control vs DCM1+ 2, LVNC vs resLVNC and DCM1+ 2 vs resDCM1. **d** Measurements of resting Ca^2+^ (transient baseline) and Ca^2+^ transient amplitude with ratiometric dye Fura-2-AM at 0.5 Hz pacing. Data is shown in bar graph with mean ± SEM, where each dot represents one measured cells from multiple cardiac differentiations with the black colored dot representing the mean of one independent FURA measurement: [number of differentiations/analyzed cells/measured dishes] for control [4/57/12], LVNC [4/55/16], resLVNC [2/22/7], DCM1+ 2 [5/59/18], and resDCM1 [3/35/12]. *P*-values were calculated nested *t*-test for control vs LVNC, control vs DCM1, LVNC vs resLVNC, and DCM1 vs resDCM1

Cardiac contractility is governed by the processes of excitation-contraction (EC) coupling, where Ca^2+^ handling plays a key role.^39^ Since we observed that RBM20 affects the splicing of Ca^2+^ handling genes such as *RYR2, TRDN,* or *CAMK2D*, we evaluated whether the different RBM20 mutations affect Ca^2+^ homeostasis. Employing the cytosolic Ca^2+^-dye Fluo-4-AM, we observed accelerated rates of Ca^2+^ cycling in LVNC-CM with reduced Ca^2+^ transient rise times compared to resLVNC-CM and shorter relaxation rates compared to unrelated and isogenic controls (Fig. 4a, b). In DCM-CM, a similar trend was observed, but only Ca^2+^ rise time was significantly shortened compared to resDCM1-CM (Fig. 4a, b). The dominant regulator of cardiac contractility is β-adrenergic stimulation, and therefore, we treated CM with the β-adrenergic receptor agonist Isoprenaline (Iso). While control- and DCM-CM demonstrated a prominent acceleration in rise time (time to peak) after Iso treatment, this was not the case in LVNC-CM, where the basal rates were already accelerated (Supplementary Fig. 6a). This was rescued in resLVNC-CM, which thereby also restored the β-adrenergic response. Diastolic (resting) SR Ca^2+^ release via leaky RYR2 is a key mechanism for contractile dysfunction and arrhythmias in HF and other cardiac diseases.^40^ The Ca^2+^ leak was significantly increased in DCM-CM of both patients (DCM1 and 2), but not LVNC-CM, which was reversed in resDCM1-CM (Fig. 4c). Furthermore, we investigated the impact of RBM20-mutations on resting Ca^2+^ and Ca^2+^ transient peak amplitude using the ratiometric Ca^2+^-dye Fura-2-AM. The resting Ca^2+^ content was not significantly altered between the lines (Fig. 4d), but LVNC-CM showed increased systolic Ca^2+^ transient amplitudes compared to isogenic controls, whereas DCM-CM exhibited reduced transients compared to LVNC- and control-CM (*p* = 0.054) (Fig. 4d). Taken together, the disturbed Ca^2+^ kinetics in LVNC-CM, the significant SR Ca^2+^ leak in DCM-CM, and the diametrically opposed aberrations in systolic Ca^2+^ amplitudes indicate that different RBM20 mutations dictate diverging alterations in Ca^2+^ homeostasis.

**Fig. 5:**
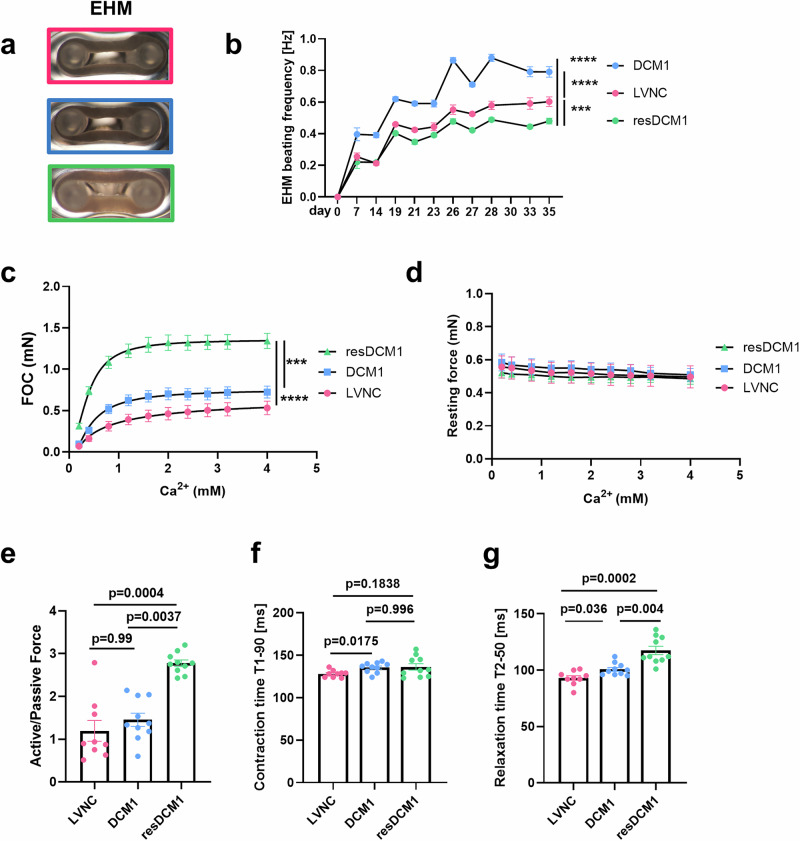
LVNC- and DCM-EHM show differential Ca^2+^ handling pathologies and contractile dysfunction. **a** Engineered human myocardium (EHM) for LVNC, DCM1, and resDCM1 on flexible holders consisting of 70% iPSC-CM and 30% human foreskin fibroblasts. **b** Beating rate of the EHM measured on different days since EHM casting (day 0 refers to the day of casting). Data is presented as superimposed symbols with mean ± SEM for *n* = 24 EHM per cell line. Two-Way ANOVA for day 35 resDCM1 vs LVNC and resDCM1 vs DCM1. **c**–**g** 9-10 EHM were used for isometric force measurements for each line. **c** Force of contraction (FOC) measurements under rising Ca^2+^ concentrations. Data is presented as superimposed symbols with mean ± SEM. *P*-values were calculated by Two-way RM ANOVA with Geisser-Greenhouse´s correction. *P*-value (column factor) against resDCM1 vs DCM1 and resDCM1 vs LVNC (significant values are marked with *p < 0.05, **p < 0.01, ***p < 0.001, and ****p < 0.0001). **d** Resting force measurements under rising Ca^2+^ concentration. Data is presented as superimposed symbols with mean ± SEM. P-values were calculated by Two-way RM ANOVA with Geisser-Greenhouse´s correction. *P*-value (column factor) against resDCM1 vs DCM1 and resDCM1 vs LVNC (not significant). **e**–**g** *P*-value by Kruskal–Wallis test with Dunns’s multiple comparison test (**e**) or Brown-Forsythe and Welch ANOVA with Dunnett´s multiple comparisons test (**f**, **g**). Data is presented as bar graph with mean ± SEM, whereas each dot represents one independent EHM experiment. **e** Active/passive force (at 4 mM Ca^2+^) ratio. **f** Contraction time (T1-90: to 90% of baseline) of EHM in ms. **g** Relaxation (T2-50: to 50% of baseline) time of EHM in ms

Therefore, we next investigated the impact of the distinct RBM20 mutations on contractility and force generation. Engineered human myocardium (EHM) was produced from iPSC-CM, and commercially available human foreskin fibroblasts (Fig. 5a). The EHM were cast as a 3D ring structure around two flexible poles and allowed to mature for 4–6 weeks. The isogenic resDCM1 line served as control. During the 4 weeks of EHM maturation, the beating frequency is optically captured and reaches a plateau after 3-4 weeks. At day 35 after EHM casting, the LVNC- and the DCM1-EHM exhibit a significantly faster beating rate than the corresponding rescue line (Fig. 5b). As a functional end-point assessment, the EHM were measured under isometric conditions in organ baths. Both LVNC-, as well as DCM1-EHM, showed a reduced force of contraction (FOC) (active force) compared to resDCM1 (Fig. 5c). This is in line with a reduced contractility amplitude as a parameter of systolic dysfunction of 3D spheroid isolated LVNC-iPSC-CM compared to resLVNC (Supplementary Fig. 6g). In contrast, the resting force of EHM (passive force) did not differ between the lines (Fig. 5d). Importantly, the active/passive force ratio as an indicator of heart muscle dysfunction was reduced in both LVNC- and DCM1-EHM, compared to resDCM1-EHM underscoring a pathological contractility when an RBM20 mutation is present (Fig. 5e). Of note, the LVNC iPSC-CM number in the tissue was reduced compared to all other lines despite the same cell numbers being used for casting the EHM. In contrast, the amount of α-actinin-positive cells did not differ between LVNC and all other lines used (Supplementary Fig. 6c–e). Accordingly, the FOC per iPSC-CM (in EHM) was preserved in contrast to the 3D tissue (Supplementary Fig. 6f). Lastly, the contraction time was reduced for LVNC-EHM compared to DCM-EHM, whereas the relaxation time was reduced in both LVNC- and DCM-EHM compared to resDCM1, with LVNC exhibiting the most prominent decrease (Fig. 5f, g). This is in line with the accelerated Ca^2+^ kinetics data (Fig. 4b).

### Increased cAMP levels and increased PLN phosphorylation events are specific to LVNC-CM, but not DCM-CM

**Fig. 6:**
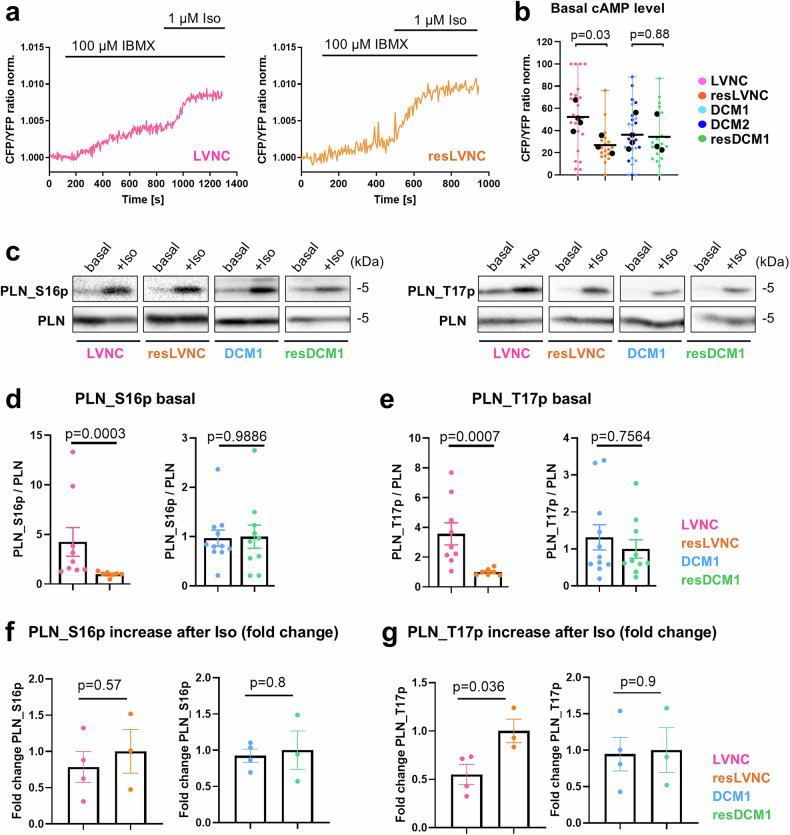
LVNC-CM exhibit increased cAMP levels and altered PKA- and CAMK2D-dependent phosphorylation of PLN. **a** Representative cAMP FRET traces from the EPAC-cAMP-FRET sensor in adenovirally transduced iPSC-CM from indicated patient groups at basal level. The maximal FRET response was induced using the unselective phosphodiesterase (PDE) inhibitor (IBMX) (100 µM). **b** LVNC-CM show elevated basal cAMP levels. Quantification of maximal FRET response to IBMX at basal state. Data is presented as mean with range showing all data points, whereas the large black dots represent the mean of an individual differentiation with [number of differentiations/analyzed cell]: LVNC [4/22], resLVNC [3/18], DCM1+ 2 [4/24], and resDCM1 [3/18]. *P*-values were calculated with nested *t*-test between the respective groups LVNC vs resLVNC and DCM1+ 2 vs resDCM1. **c** Representative original Western blot membranes stained for PLN and its PKA-phosphorylation site S16p (left) and its CAMK2D-phosphorylation site T17p (right). Samples were taken unstimulated (basal) and after Iso treatment (1 µM for 15 min). **d** + **e** Quantification of PLN-S16p and PLN-T17p in untreated iPSC-CM. Data is shown in bar graph with mean ± SEM, whereas every dot represents one cardiac differentiation for LVNC,[9] resLVNC,[7] DCM1 [11], and resDCM1.[10] The *p*-values were calculated by Mann–Whitney test. **f** + **g** Quantification of PLN-S16p and PLN-T17p in Iso treated iPSC-CM (1 µM, 15 min). Increase in phosphorylation after Iso treatment shown as the fold change from basal to Iso level and normalized to respective isogenic line, therefore, LVNC-CM exhibit a weaker increase in PLN-T17p level after Iso. Data is shown in bar graph with mean ± SEM, whereas every dot represents one cardiac differentiation. The *p*-values were calculated by Student´s t-test. Iso = isoprenaline

Due to RBM20-dependent alterations in Ca^2+^ homeostasis, we sought to investigate the β-adrenergic signaling pathway, including the second messenger cAMP, as well as protein kinase A (PKA) and CAMK2D activity. The cAMP dynamics were measured by transduction of iPSC-CM with an adenoviral construct expressing the Förster resonance energy transfer (FRET)-based cAMP sensor Epac1-cAMPs under the control of the cytomegalovirus promoter.^41^ First, we analyzed cAMP synthetization under basal conditions by pretreating iPSC-CM with the unselective phosphodiesterase (PDE) inhibitor 3-isobutyl-1-methylxanthin (IBMX) in the absence of Iso (Fig. 6a). We detected significantly higher FRET responses in LVNC-CM compared to resLVNC-CM, whereas DCM-CM did not differ from their isogenic control (Fig. 6b), suggesting increased basal cytoplasmic cAMP levels in LVNC-CM. Upon Iso treatment, we did not observe differences in CFP/YFP ratios between the groups (Supplementary Fig. 6b). Next, we explored the phosphorylation status of proteins involved in Ca^2+^ cycling. One protein that strongly influences Ca^2+^ reuptake into the SR, and therefore the Ca^2+^ transient decay time, is the SERCA-inhibitory protein phospholamban (PLN). PLN´s inhibitory effect can be ameliorated by phosphorylation by protein kinase A (PKA) of serine 16 (S16p) and CAMK2D of threonine 17 (T17p). Therefore, we focused on the phosphorylation status of PLN in basal and Iso-treated samples. At baseline, the LVNC-CM exhibited significantly higher phosphorylation of PLN-S16p by PKA and PLN-T17p by CAMK2D compared to resLVNC-CM (Fig. 6c–e). As expected, Iso-treated iPSC-CM showed a prominent phosphorylation increase in all samples (Fig. 6c). However, compared to the phosphorylation increase in resLVNC, the LVNC-CM showed a significantly lower rise in phosphorylation events after Iso for the CAMK2D site PLN-T17p, which could be due to higher phosphorylation levels at basal state, indicating that the CAMK2D-dependent PLN-T17p site is affected to a higher extent. Like the cAMP findings, DCM-CM do not show any deviations in phosphorylation status of PLN before and after Iso treatment (Fig. 6c–g).

In conclusion, under basal conditions, key regulatory enzymes involved in cytosolic Ca^2+^ handling are hyperphosphorylated in LVNC-CM, but not DCM-CM. In LVNC-CM, the elevated levels of cAMP with subsequently higher phosphorylation of PLN by PKA and CAMK2D could be the driver for the distinctly fastened Ca^2+^ kinetics in LVNC-CM.

### Increased mitochondrial respiration in LVNC-CM, but not DCM-CM

**Fig. 7:**
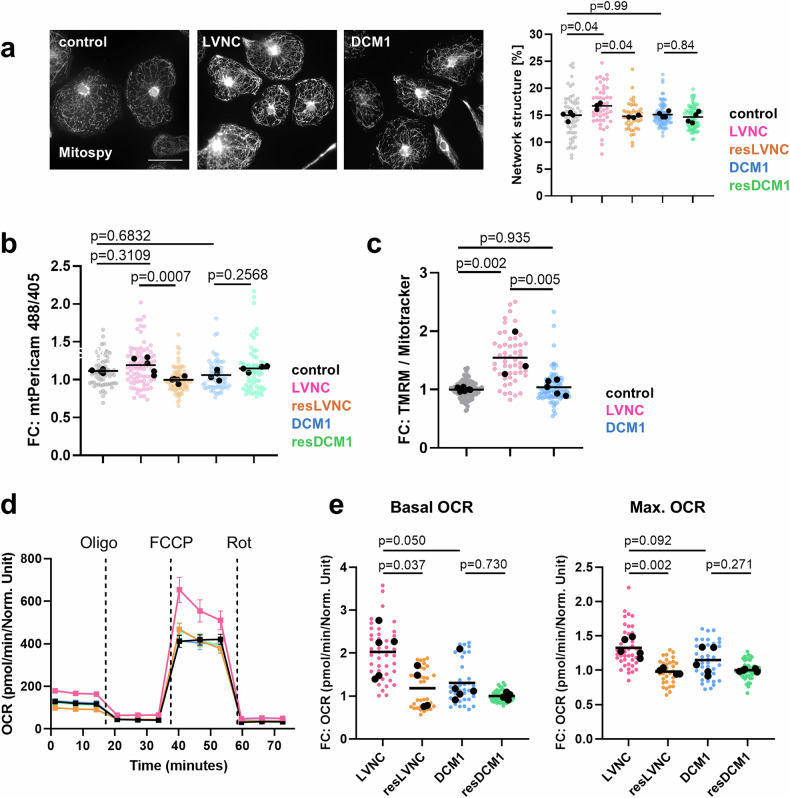
Mitochondrial and metabolic profiling of LVNC- and DCM-CM. **a** Representative Mitospy-stainings. Brightness and contrast were enhanced for visualization purposes. Scale bar: 50 µm. Quantified mitochondrial network in [%] using the MiNa ImageJ plugin. Data is presented as scatter plot with mean showing all data points with the large black dots representing the mean of one cardiac differentiation: [number of differentiations/analyzed images] for control [4/59], LVNC [3/45], resLVNC [3/40], DCM1 [4/81] and resDCM1 [4/71]. *P*-values were calculated by nested 1way ANOVA with Sidak´s comparisons for control vs LVNC, control vs DCM1, LVNC vs resLVNC, and DCM1 vs resDCM1. **b** Measurement of mitochondrial Ca^2+^ in iPSC-CM using plasmid-encoded mtPericam, a ratiometric Ca^2+^ probe that localizes to the mitochondria. LVNC-CM shows elevated levels of mitochondrial Ca^2+^. To compare measurements from different days, the samples were normalized resLVNC-CM, which were part of every experiment. Data is presented as scatter plot with mean, whereas the large black dots represent the mean of one cardiac differentiation with [number of differentiations/analyzed images] for control [4/60], LVNC [5/75], resLVNC [5/75], DCM1 [4/57], and resDCM1 [4/60]. *P*-values were calculated by nested 1way ANOVA with selected comparisons by Sidak´s multiple comparisions test for control vs LVNC, control vs DCM1, LVNC vs resLVNC, and DCM1 vs resDCM1. **c** TMRM measurement of mitochondrial membrane potential. To compare measurements from different days the samples were normalized to control. LVNC-CM show increased mitochondrial membrane potential. Data is presented as scatter plot with mean, whereas the large black dots represent the mean of one cardiac differentiation: [number of differentiations/analyzed images] for control [7/110], LVNC [3/51], and DCM1 [5/79]. *P*-values were calculated with nested 1way ANOVA with Tukey´s correction. **d** Representative Seahorse-curves for LVNC-, resLVNC-, DCM1- and resDCM1-CM. **e** The iPSC-CM from LVNC, resLVNC, DCM1, and resDCM1 were evaluated for basal and maximal respiration capacity. The measurements were normalized by total protein level. To display measurements from different plates in one graph, the data were normalized to resDCM1, which was part of every measurement. Data is presented as scatter blot with mean displaying all data points with the large black dots representing the mean of one cardiac differentiation: [number of differentiations/analyzed wells] for LVNC [5/40], resLVNC [4/32], DCM1 [4/32], and resDCM1 [5/40]. *P*-values were calculated by nested 1way ANOVA with selected comparisions by Sidak´s multiple comparisions test for LVNC vs resLVNC, LVNC vs DCM1, and DCM1 vs resDCM1. FC fold change, OCR oxygen consumption rate

EC coupling consumes large amounts of energy that is replenished by oxidative phosphorylation in mitochondria. To constantly adapt mitochondrial energetics to energy consumption, mitochondria take up Ca^2+^, which stimulates Krebs cycle dehydrogenases to fuel the respiratory chain with electrons, establishing the mitochondrial membrane potential (ΔΨ_m_), which in turn drives ATP production.^42^ Therefore, we explored the impact of both mutations on mitochondrial and metabolic function. Visualizing the mitochondrial network with Mitospy revealed that compared to control, this was more prominently developed in LVNC-CM, but not DCM-CM (Fig. 7a). Transfection with a genetically encoded mitoPericam-probe revealed distinct mitochondrial Ca^2+^ signals, which were verified by co-localization with TMRM (Supplementary Fig. 6h). In fact, we observed higher mitochondrial Ca^2+^ levels in LVNC-CM than DCM1-CM, which decreased in the isogenic resLVNC-CM (Fig. 7b). Further, the mitochondrial membrane potential ΔΨ_m_ was increased in LVNC-CM, but not DCM-CM, compared to control (Fig. 7c). Accordingly, both basal and maximal oxygen consumption rates (OCR) were elevated in LVNC-CM compared to resLVNC-CM (Fig. 7d, e). These results suggest a higher metabolic activation that is distinct to the LVNC-phenotype.

### Pharmacological intervention in the therapy of RBM20-based cardiomyopathies

**Fig. 8:**
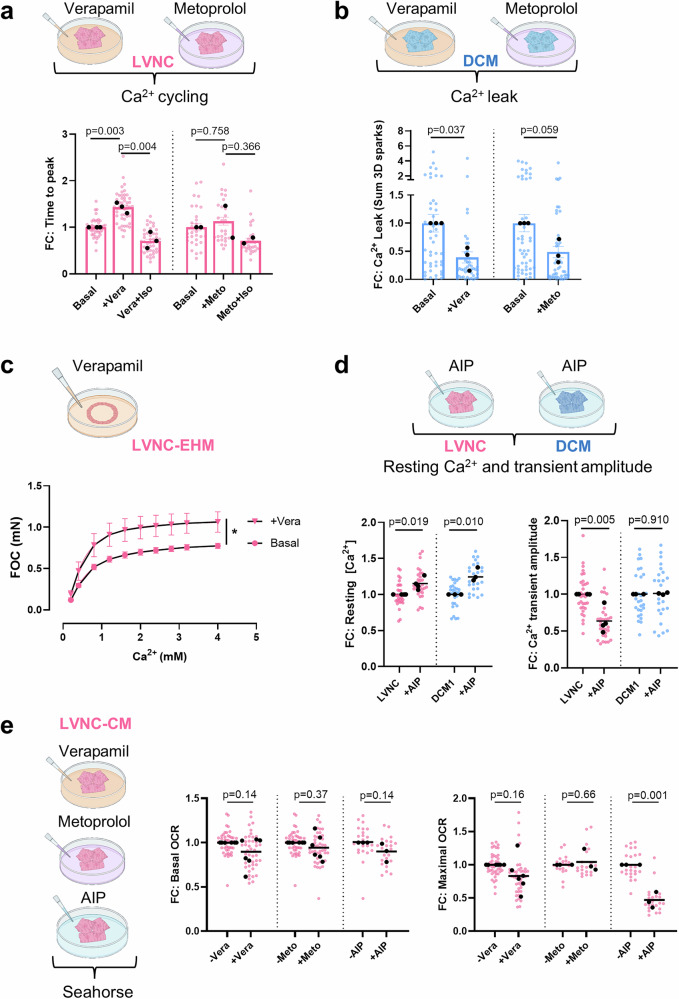
Pharmacological intervention in the therapy of RBM20-based cardiomyopathies. **a** Pharmaceutical intervention with Ca^2+^ channel- (verapamil) and β-blocker (metoprolol) in LVNC-CM their effects on Ca^2+^ kinetics. After basal measurement, the cells were incubated for 15 min with verapamil (30 nM) or metoprolol (5 µM). For each measurement, the data is normalized to the untreated (basal) condition. Data is presented as bar graph with mean ± SEM. Each pink colored dot represents a single LVNC-CM cell, whereas and the large black colored dots represent the mean of one cardiac differentiation: [number of differentiations/analyzed cells] for verapamil set: Basal – Vera – Vera+Iso [3/38 – 3/45 – 3/41]. Metoprolol set: Basal – Meto – Meto+Iso [2/29 – 2/27 – 2/28]. P-values were calculated by nested t-test for Basal vs Vera; Vera vs Vera+Iso; Basal vs Meto and Meto vs Meto+Iso. **b** Pharmaceutical intervention with Ca^2+^ channel- (verapamil) and β-blocker (metoprolol) in DCM1-CM their effects on Ca^2+^ leakage. After basal measurement, the cells were incubated for 15 min with verapamil (30 nM) or metoprolol (5 µM). For each measurement, the data is normalized to the untreated (basal) condition. Data is presented as bar graph with mean ± SEM. Each blue colored dot represents a single DCM1-CM cell, whereas and the large black colored dots represent the mean of one cardiac differentiation: [number of differentiations/analyzed cells] for Basal – Vera [3/44–3/45] and Basal – Meto [3/47 – 3/45]. *P*-values were calculated by nested *t*-test. **c** Treatment of LVNC-EHM for 7 days with verapamil (30 nM) and subsequent analysis of FOC (force of contraction). Data is presented as superimposed symbols (*n* = 3 independent EHM per condition) with mean ± SEM. *P*-values were calculated by Two-way ANOVA with Tukey’s multiple comparison test. *P*-value is marked with *p < 0.05, **p < 0.01, ***p < 0.001 and ****p < 0.0001. **d** Treatment of LVNC- and DCM1-CM with a CAMK2D-inhibitor (AIP) and its effect on resting Ca^2+^ and Ca^2+^ transient amplitude. After basal measurements, cells were treated with AIP (1 µM) for 15 min. Data shows all single cell measurements (colored) as scatter plot with mean, whereas and the large black colored dots represent the mean of one cardiac differentiation. Data is presented as fold change (FC) with the basal condition of each experiment set to 1. [number of differentiations/analyzed cells] for LVNC basal [4/31] and AIP [4/29], and DCM1 basal [3/28] and AIP [3/24]. *P*-values were calculated by nested *t*-test. **e** Pharmaceutical intervention with Ca^2+^ channel- (verapamil), β-Blocker (metoprolol), or CamKII-inhibitor AIP in LVNC-CM and their effects on OCR (oxygen consumption rate). Cells were treated for 24 h with verapamil (30 nM), metoprolol (5 µM), or AIP (1 µM). Data is normalized to the basal condition and depicted as fold change (FC). Data shows all single cell measurements (colored) as scatter plot with mean, whereas the large black colored dots represent the mean of one cardiac differentiation. For basal OCR [number of differentiations/analyzed LVNC-CM] basal vs vera [6/43 vs 6/42]; basal vs meto [6/43 vs 6/41] and basal vs AIP [3/22 vs 3/22]. For maximal OCR [number of differentiations/analyzed LVNC-CM] basal vs vera [6/42 vs 6/41]; basal vs meto [3/19 vs 3/19] and basal vs AIP [3/23 vs 3/22]. *P*-values were calculated by nested *t*-test. Vera Verapamil, Meto Metoprolol, FC Fold change, OCR oxygen consumption rate

Based on the disturbed Ca^2+^ handling, we explored possibilities for drug intervention. Based on their clinical phenotype of HFrEF, both patients received guideline-recommended therapy with β-blockers, angiotensin receptor blockers, and mineralocorticoid receptor antagonists (Supplementary Table 1). However, since Ca^2+^ transient amplitudes and Ca^2+^ handling kinetics were paradoxically elevated in LVNC-CM, we examined whether Ca^2+^ channel blockade with verapamil or inhibition of CAMK2D with autocamide-2-related inhibitory peptide (AIP) would improve abnormal Ca^2+^phenotypes and compared these to the effects of the β-blocker (metoprolol), since cAMP and CAMK2D signaling-both under the control of β-adrenergic stimulation^43^ were also altered in both cell lines. In fact, treatment with verapamil (30 nM) reduced Ca^2+^ transient rise kinetics and restored the physiological response to β-adrenergic stimulation in LVNC-CM (Fig. 8a). These effects were more pronounced for verapamil than for metoprolol (5 µM), as metoprolol treatment only restored the Iso response but did not affect basal Ca^2+^ kinetics (Fig. 8a). Furthermore, verapamil (30 nM) and metoprolol (5 µM) significantly decreased the Ca^2+^ leak in DCM1-CM (Fig. 8b). To supplement these findings, we treated LVNC-EHM with verapamil (30 nM) for 7 days and observed an increase in systolic force generation upon verapamil treatment (Fig. 8c). In addition, we explored whether the CAMK2D inhibitor AIP improves the phenotype observed in LVNC-CM, since previous analyses revealed CAMK2D mis-splicing, accompanied by increased phosphorylation of CAMK2D-dependent PLN-T17p. Treating LVNC- and DCM-CM with AIP (1 µM) resulted in elevated resting Ca^2+^ levels for both LVNC- and DCM1-CM (Fig. 8d), whereas no effect was observed in control iPSC-CM (Supplementary Fig. 6i). Intriguingly, the Ca^2+^ transient amplitude is only significantly decreased in LVNC-CM, but not DCM-CM, indicating a more prominent role for CAMK2D as a player for the elevated systolic Ca^2+^ transient amplitude in LVNC (Fig. 8d). Since elevated respiratory capacity in LVNC-CM may be related to the increased energetic demand imposed by activation of EC coupling, we examined whether verapamil, metoprolol or AIP would also attenuate the elevated metabolic activity. However, while metoprolol had no effect on basal or maximal OCR, verapamil showed a strong trend in that direction (Fig. 8e). Intriguingly, AIP has a clear influence on respiration by significantly decreasing the maximal OCR (Fig. 8e). Based on these findings, Ca^2+^ channel blockade with verapamil may be a promising personalized treatment approach in the patient with the RBM20-induced LVNC phenotype, which necessitates further clinical investigation.

### DCM mutation p.R634W in LVNC background mirrors the DCM phenotype

**Fig. 9:**
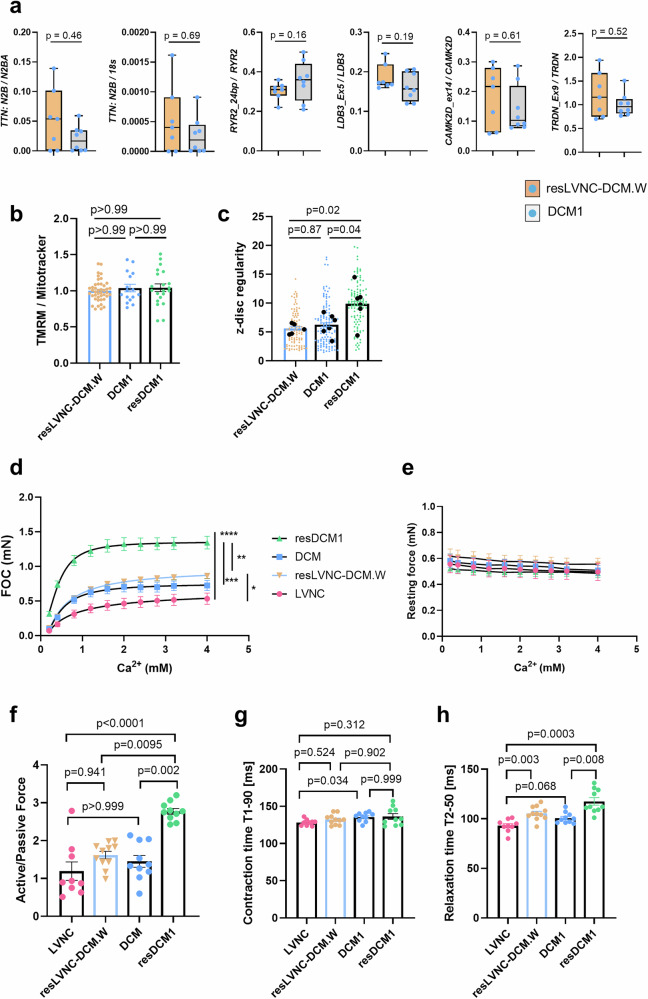
ResLVNC-DCM.W mirror the DCM phenotype. **a** QPCR analysis of RBM20 splice targets in resLVNC-DCM.W vs DCM1, which both harbor the DCM mutation RBM20 p.R634W. Primers were designed to target the exon of interest, and expression levels were normalized to the total gene expression by using primers directed against a constitutively expressed exon. Data is shown as box plots, whereas every dot represents one differentiation experiment. No significant differences were detected for splicing of the N2BA and N2B domain of *TTN*, an intronic 24 bp insertion of *RYR2*, exon 14 inclusion in *CAMK2D*, exon 9 splicing in *TRDN* and exon 5 splicing in *LDB3*. P-values were determined by Mann–Whitney test. **b** TMRM measurement of mitochondrial membrane potential. To compare measurements from different days the samples were normalized to resLVNC-DCM.W. Bar graphs depicts mean ± SEM showing all data points for [number of differentiations/analyzed pictures] for resLVNC-DCM.W [2/43], DCM1 [1/16], and resDCM1 [1/21]. *P*-values were determined by Kruskal–Wallis multiple comparisons with Dunn´s correction. **c** Quantification of Z-disc sarcomeric regularity (α-actintin) using Fast Fourier transformation calculating the peak amplitude of the first-order peak. Bar graphs depicts mean ± SEM showing all data points, whereas the large dots represent the mean of one cardiac differentiation for resLVNC-DCM.W [5/99], DCM1 [6/117], and resDCM1 [6/105]. *P*-values were determined by nested 1way ANOVA with Tukey´s multiple comparisons. **d**–**h** EHM data for resLVNC-DCM.W (*n* = 11 EHM). ResLVNC-DCM.W resembles the DCM phenotype and differs from LVNC. **d** Force of contraction (FOC) measurements under rising Ca^2+^ concentrations. The resLVNC-DCM.W-EHM is almost congruent with the DCM1-EHM measurements. P-values were calculated by Two-way RM ANOVA with Geisser-Greenhouse correction. P-value (column factor) for resDCM1 vs DCM1; LVNC vs resDCM1; resDCM1 vs resLVNC-DCM.W; LVNC vs resLVNC-DCM.W (significant values are marked *p < 0.05, **p < 0.01, ***p < 0.001) and DCM1 vs resLVNC-DCM.W (not significant). **e** Resting Force measurements under rising Ca^2+^ concentration. P-values were calculated by Two-way RM ANOVA with Geisser-Greenhouse´s correction. P-value (column factor) for resDCM1 vs DCM1 and resDCM1 vs resLVNC-DCM.W, LVNC vs resDCM1 and DCM1 vs resLVNC-DCM.W (not significant). **f**–**h** *P*-value by Kruskal-Wallis with Dunn´s multiple comparisons (F) or Brown-Forsythe and Welch ANOVA with Dunnett´s multiple comparisons test (G, H). Data is presented as bar graph with mean ± SEM with each dot representing one independent EHM experiment. **f** Active/passive force (at 4 mM Ca^2+^) calculation. **g** Contraction time (T1-90: to 90% of baseline) of EHM in ms. **h** Relaxation (T2-50: to 50% of baseline) time of EHM in ms

Since the two RBM20 variants show distinct phenotypes, we used the resLVNC-line to introduce the DCM-variant p.R634W using CRISPR/Cas9 (Supplementary Fig. 7a). Similar to all the other lines, the newly generated iPSC are positive for pluripotency markers (Supplementary Fig. 7b) and retained a normal karyotype (46, XX) after the second gene edit (Supplementary Fig. 7c). This novel line, termed resLVNC-DCM.W, represents the DCM variant but now in a LVNC background to address the question of whether the DCMvariant per se will cause a DCM phenotype or if the genetic background or sex of the LVNC patient contributes to the development of the LVNC phenotype. Intriguingly, resLVNC-DCM.W-CM exhibited a phenotype closer to the DCM than the LVNC. We observed the same splice profile as for DCM1 (Fig. 9a), no alteration in ΔΨ_m_ and decreased sarcomeric regularity compared to resDCM1 (Fig. 9b, c). Furthermore, the force of contraction of the resLVNC-DCM.W-EHM was virtually identical to the force generation of the DCM-EHM, and resting force was similarly elevated as in the DCM-EHM (Fig. 9d–f). Accordingly, the relaxation time resembled the DCM-EHM rather than the LVNC-EHM phenotype (Fig. 9g, h).

## Discussion

**Fig. 10:**
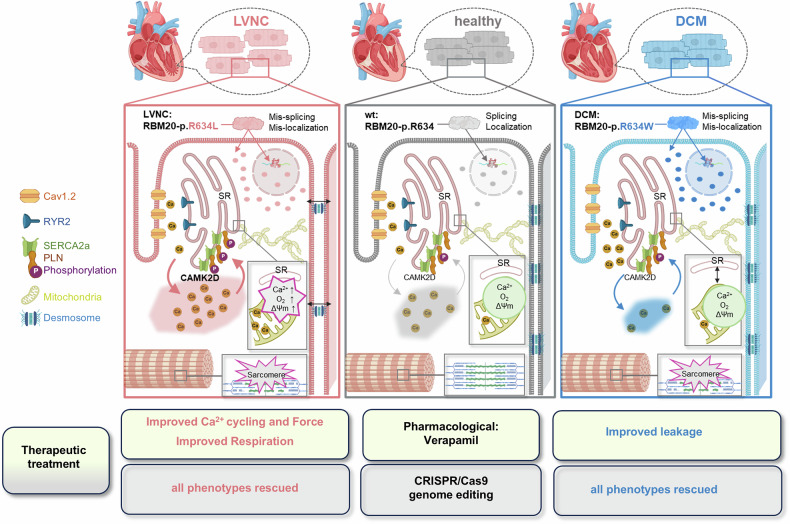
Summary Fig.: RBM20 variants have shared and differential pathologies. LVNC-Variant RBM20-p.R634L causes desmosomal derangement, fastened Ca^2+^ cycling, elevated systolic Ca^2+^ amplitudes, and enhanced respiration. The DCM-variant RBM20-p.R634W causes greater spatial distance between SR (sarcoplasmic reticulum) and mitochondria, elevated Ca^2+^ leakage, and decreased systolic Ca^2+^ amplitudes. Both variants result in RBM20 mis-localization, distinct target-gene mis-splicing, sarcomeric disarray, and reduced force generation. Verapamil has positive effects on both variants. CRISPR/Cas9 genome editing rescued all phenotypes. Created in BioRender; BF29NGRZUQ

It is well established that in HFrEF, a reduced amplitude of cytosolic Ca^2+^ transients and slowed reuptake of Ca^2+^ into the SR are the dominant causes for systolic and diastolic dysfunction.^44^ Furthermore, antagonization of neuroendocrine activation typically ameliorates these defects in EC coupling and is therefore the mainstay of pharmacological treatment, irrespective of the underlying etiology of HFrEF.^44,45^ Here, we revealed that in RBM20-related cardiomyopathy, a relevant cause for non-ischemic DCM, a single nucleotide can determine the clinical and cellular phenotype and potentially, the therapeutic responsiveness towards personalized treatment approaches. In a human patient-specific approach, we show that two distinct missense mutations in the same amino acid of RBM20 (R634) can lead to very different functional and metabolic phenotypes of EC coupling and mitochondrial energetics (Fig. 10).

While both patients have HFrEF, which was closely mirrored by substantially decreased force generation in our EHM studies, RBM20-p.R634W-based DCM-CM display the “classical” cellular DCM/HFrEF phenotype, with diminished Ca^2+^ transient amplitudes, increased resting Ca^2+^ leak, and disturbed spatial association between the SR and mitochondria. In contrast, a Leu substitution of RBM20-position 634 leads LVNC-CM to adopt a more pre-activated state characterized by an overstimulated β-adrenergic system with elevated cAMP levels, high PLN phosphorylation, elevated systolic and mitochondrial Ca^2+^ transient amplitudes, accelerated Ca^2+^ kinetics, and higher energy consumption, already at rest, likely reflecting a compensatory response to the ultrastructural impaired desmosomal integrity responsible for the reduced compaction of the tissue. On the molecular level, we observed erroneous splicing and cytoplasmatic accumulation of RBM20 as disease drivers for both LVNC and DCM, while specifically in LVNC, mis-splicing of *CAMK2D* and desmosome genes likely causes the observed hyperactivation and non-compaction morphology.

It is currently a substantial matter of debate whether the primary disease drivers in RBM20 variants are erroneous splicing, gain of (maladaptive) function through RBM20 cytoplasmic accumulation, or lack of function by RBM20 abundance, or a combination of any of these. For all RBM20 mutations and knock-out models, mis-splicing was observed across multiple target genes, consistently including *TTN.*^15^ On the other hand, recent reports have highlighted that aberrant cytoplasmic RBM20-granulae formation is the pathological culprit, increasingly viewing RBM20-cardiomyopathies as a granulae/condensate disease rather than a definite spliceopathy.^15^ Reducing RBM20 or increasing RBM20 nuclear import both improve the underlying DCM phenotype.^46,47^ However, for RBM20 variants outside the RS domain, mis-splicing without RBM20 cytoplasmic accumulation was observed, resulting in a DCM phenotype.^15^ Here, we report RBM20 granulae for both missense variants and shared mis-splicing for several target genes exemplified by *TTN* and *RYR2*. Additionally, distinct mis-splicing events occur for other targets depending on the RBM20 variant, e.g., for Ca^2+^ handling genes (*CAMK2D*, *TRDN*) in LVNC-CM concomitant with a distinct Ca^2+^ handling phenotype. Since the CAMK2D inhibitor AIP reduced elevated Ca²⁺ transient amplitude and increased mitochondrial respiration in LVNC-iPSC-CM, CAMK2D seems to be a key effector in LVNC. However, broader phenotypes likely result from multiple mis-spliced targets, which warrants further investigation. Notably, AIP showed differential Ca²⁺ effects, reducing Ca^2+^ transient amplitude but elevating potentially harmful diastolic Ca²⁺ in both LVNC- and DCM-CM. This indicates that differential mis-splicing, rather than cytoplasmic RBM20 aggregation (observed in both variants), primarily drives the pathologies, as supported by another study showing variant-specific effects on mRNA splicing.^48^ The sequencing data support this hypothesis, as overall gene expression profiles do not substantially differ between LVNC- and DCM-specific phenotypes in Ca^2+^ and metabolic homeostasis. Rather, the differential exon usage of RBM20 target genes shows more pleiotropic differences between RBM20-mutation-dependent LVNC and DCM. However, this should be taken with caution, as the mis-localization and RBM20-granulae formation require deeper investigation regarding their spatiotemporal resolution and organelle interaction. Aberrant granulae formation underlies many neurodegenerative diseases, in which temporal and dynamic localization of aggregates are key to understanding the pathomechanism.^49,50^ As a common theme, we observe *TTN* mis-splicing and sarcomeric disarray, a shared pathology across all RBM20 variants.^15^ Ca^2+^ handling aberrations are also common in RBM20-dependent cardiomyopathies. However, these vary depending on the specific RBM20 variant, highlighting that RBM20 mutations can lead to different clinical outcomes. A study in RBM20 knockout mouse models reported increased systolic Ca^2+^, SR Ca^2+^ load, and Ca^2+^ leakage,^24^ suggesting that full RBM20 knockout affects multiple aspects of Ca^2+^ homeostasis. In this study, we made a direct side-by-side comparison of elevated systolic Ca^2+^ without Ca^2+^ leakage in LVNC-CM, whereas DCM-CM showed Ca^2+^ leakage but reduced systolic Ca^2+^. In our previous study of iPSC-CM with RBM20-p.S635A, we reported decreased resting and increased systolic Ca^2+,^ ^22^ similar to LVNC-CM here, but p.S635A/p.P633L showed increased Ca²⁺ transient rise times, contrasting LVNC-CM’s faster kinetics.^22,28^ Treatment with the Ca^2+^ channel blocker verapamil and the CAMK2D inhibitor AIP showed promising potential by restoring multiple aspects of Ca^2+^ handling and even force of contraction. This is in line with the previous observation that verapamil treatment attenuated Iso-induced Ca^2+^ leakage in RBM20 knockout mice.^24^ Although, unlike AIP, verapamil is already available in the clinic, it is contraindicated in patients with HFrEF due to its profound negative inotropic effects.^45^ However, our in vitro data generates the hypothesis that, in earlier disease states before HFrEF unfolds, the use of verapamil may prevent the development of a HFrEF phenotype in patients with the LVNC-inducing RBM20 variant. While limited studies have investigated Ca^2+^ channel blockers in heart failure with preserved or mildly reduced ejection fraction, these studies suggest safety and potential improvements in exercise capacity and symptoms.^51^ Together, our preclinical data thus suggest that verapamil could be cautiously explored in a personalized approach for LVNC-causing RBM20 variants prior to overt HFrEF.

To translate the in vitro findings of this study into therapeutic implications, in vivo validation is usually required; however, recent regulatory reforms, including the FDA Modernization Act 3.0, now support new approach methodologies (NAMs) as supplements or alternatives to animal testing.^52^ Along these lines, another study has already provided evidence that drug testing in patient-specific iPSC-CM and a tailored medication switch can be beneficial for individual patients.^23^ This underscores how iPSC-CM can serve as a diagnostic tool guiding therapy in cardiac personalized medicine. Finally, it should be noted that rescue of the gene mutation reversed the majority of phenotypes observed, suggesting that gene and base editing, especially for RBM20, are feasible approaches. Although this is not yet available in the clinics, the first reports show promising results.^53^

The EHM results show a uniform decrease in systolic force generation for LVNC- and DCM-CM. The EHM data are suggestive of two hypotheses: First, the ultrastructurally impaired desmosome integrity in LVNC is responsible for a loss of contractile cells and the reduced compaction of the tissue, leading to lower force generation in LVNC-EHM. Second, the hyperactivated LVNC-CM with increased Ca^2+^ handling and metabolism at the cellular level likely reflects a compensatory response to organ-level dysfunction. This partially resembles the cellular and global phenotypes of plakophilin 2 (PKP2)-related arrhythmogenic cardiomyopathy, with increased cellular Ca²⁺ transients despite in vivo LV dysfunction, potentially due to desmosomal defects impairing force transmission.^54,55^ Furthermore, the chronic endogenous activation of adrenergic signaling in LVNC-CM not only blunts the β-adrenergic reserve during exercise but may also contribute to maladaptive cardiac remodeling and dysfunction, a well-established consequence of chronic adrenergic activation, driven by PKA and CAMK2D-dependent mechanisms.^44^

In contrast, in the DCM-CM, decreased systolic Ca^2+^ concentrations correlate with reduced systolic force generation, resembling rather the “classical” defects in EC coupling in HFrEF.^44^ Furthermore, we and others previously revealed that in HFrEF, defective mitochondrial Ca^2+^ uptake contributes to energetic deficit and oxidative stress, a concept termed “mechano-energetic uncoupling” in HF.^42^ Mitochondrial Ca²⁺ uptake depends on the close SR mitochondria distance, and increased organelle distance impairs this process.^56^ Such structural uncoupling has been observed in human DCM^57^ and was likewise evident in our DCM-CM model compared to LVNC. Together, these data indicate that the DCM-causing RBM20 variant (R634W) induces a cellular phenotype that shares most properties of the classical HFrEF phenotype and may therefore also be optimally treated with guideline-directed HF therapies.

This is the first study of metabolic aberrations in an RBM20 missense variant, in which activated EC coupling in LVNC-CM was accompanied by metabolic activation, essential for the high energy demands of Ca²⁺ cycling and chronic adaptation via Ca²⁺-dependent pathways, including CAMK2D, that eventually converge to induce biogenesis.^58^ This contrasts with the declining metabolic activity observed in rare loss-of-function RBM20 mutations.^26^ Verapamil, metoprolol, or AIP attenuated elevated respiratory rates (though not always significantly), suggesting that the metabolic phenotype is partially dependent on Ca^2+^ aberrations in LVNC-CM. Since granulae were shown to damage mitochondria in neurodegenerative diseases,^50^ their role in cardiac RBM20 diseases and their impact on metabolism of RBM20 variants without mis-localization warrants investigation. Thus, metabolic phenotypes depend on the RBM20 variant, implying not all carriers benefit from metabolic therapies.

### Study limitations

A limitation of the current study is the inherent immaturity of iPSC-CM. It has been shown that iPSC-CM resemble fetal-like CM more closely than adult CM and could therefore mask possible phenotypes.^59^ In this study, we use prolonged culture times of 60-90 days or culture in 3D cardiospheres and EHM constructs to enhance maturation, as previously shown.^60,61^ Nevertheless, some functional/metabolic endpoints are still based on long-term 2D cultures, and therefore, caution is warranted when extrapolating to adult human myocardium.

Furthermore, the patient’s genetic background, sex, and age may influence the results, which we partially circumvented by using isogenic lines in addition to control lines. Nevertheless, due to the limited number of patients used here, this study represents a thorough investigation on the patient-specific level and is therefore too limited to draw definite conclusions regarding broader RBM20 populations and therapeutic relevance. In this line, the study is limited since only two male (DCM) and one female (LVNC) patient were analyzed, so potential sex-specific differences in gene expression or splicing cannot be assessed. While previous studies report mixed findings regarding sex effects in RBM20 cardiomyopathy, our dataset is too limited to draw conclusions.

Another limitation of the study is that single iPSC-CM and EHM are not feasible for generating pCa-force curves and assessing myofilament Ca^2+^ sensitivity using permeabilized cardiomyocytes. While most experiments were performed with high n-numbers across multiple independent samples, a few analyses (e.g., isoprenaline-treated Western blots) have lower *n*-numbers that could potentially be increased in future studies.

In conclusion, RBM20 variants are often discussed as one entity. Still, this report highlights that mutation matters and results in different pathologies, which should be considered when moving forward in personalized medicine.

## Material and methods

### Ethical statement

For reprogramming into iPSC, human cardiac tissue or blood samples were obtained in compliance with the ethical committee of the University Medical Center Goettingen (Az -10/9/15) and the ethical committee of University Heidelberg (Ethical approval number: S-329/2012).

### Cell culture

#### Reprogramming and iPSC culture

The iPSC lines for DCM1, resDCM1,^31^ and healthy control cells^21^ have previously been published by our group. For LVNC and DCM2, skin samples were obtained from the patients and reprogrammed with plasmids for LVNC or with Sendai virus for DCM2 (Supplementary Table 2). Details for reprogramming with plasmids and Sendai virus are published elsewhere.^21^

The iPSCs were cultured in monolayer in cell culture dishes coated for 30 min at 37 °C with Geltrex (167 mg/L) (Thermo Fisher Scientific). The medium Essential 8 (E8) (Thermo Fisher Scientific) was refreshed daily. Upon reaching 80–90% confluency, the cells were passaged using 3–5 min Versene (Thermo Fisher Scientific) solution at 37 °C. After passaging, iPSCs were cultured for 24 h in E8 medium supplemented with 2 µM (M = mol/l) Thiazovivin (TZV).

#### iPSC-CM differentiation

For iPSC-CM differentiation, iPSCs were plated and subsequently cultured until they reached 80–90% confluent monolayer, and Wnt signaling was modulated as previously described.^21,31^ Briefly, on day 0, the medium was exchanged for Differentiation medium (RPMI 1640 Glutamax (Thermo Fisher Scientific) with 500 mg/L Albumin and 200 mg/L L-ascorbic acid) with the addition of 4 μM CHIR 99021 (Merck). On day 2, the medium was exchanged to Differentiation medium supplemented with 2 μM Wnt inhibitor IWP2 (Merck). On day 6, the medium was changed to Differentiation medium without any additions. On day 8, the medium was changed to Cardio Culture medium (RPMI-Glutamax + B27 supplement (Gibco, 17504044)) and was subsequently refreshed every other day. CM beating was observed around day 11. Between day 14 and 21, cells were replated in Cardio Digest medium (Cardio Culture medium with 10% FCS and 2 µM TZV) onto Geltrex-coated 6 well plates at a cell number of 800.000 and after 24 - 48 h medium was replaced to glucose-deprived Selection medium (RPMI without HEPES and glucose (Thermo Fisher Scientific) with 4 mM lactate/HEPES, 500 mg/L Albumin and 200 mg/L L-Ascorbic acid) for 3–5 days. After the metabolic selection step, Cardio Culture medium was renewed twice a week. IPSC-CM were cultured for at least 60 days before used in experiments.

#### Differentiation into iPSC-EC

For EC, iPSCs were passaged onto Geltrex-coated (167 µg/mL, Gibco) 12 Well plates (Sarstedt). Upon reaching 70–85% confluency, the endothelial differentiation was initiated using a standard published small-molecule differentiation protocol.^62^ For iPSC-EC maintenance, the Endothelial Growth Medium (Merck) medium was used and refreshed every other day.

#### 3D spheroid cast and culture

To enhance maturation, iPSC-CM were cast and cultured in 3D spheroids in a fatty-acid and hormone-based maturation medium as previously described by us^32^. In short, 85 × 10^4^ iPSC-CM (25 days old) and 15 × 10^4^ iPSC-EC (passage 1-3) were transferred into a 96-well format (V-bottm, Thermo Fisher) in 150 µL Cardio Digest medium and centrifuged for 5 min at 200 g. After two days, Maturation Media I was added (Cardio Culture supplemented with Palmitate, Oleate, T3-hormone, dexamethasone, and GW7647) for 9 days and refreshed every other day. Subsequently, the medium was exchanged for Maturation Media II (Cardio Culture supplemented with Palmitate and Oleate) for at least 5 days. For contractility measurements, the spheroids were dissolved using 0.25% Tryp/EDTA for 20 min on a 37 °C shaker and subsequently stopped with FCS and plated on 35 mm dishes (MatTek).

### CRISPR/Cas9 editing of RBM20

The CRISPR/Cas9 gene editing using a crRNA-tracrRNA-Cas9 complex, HDR-template, ssDNA template, and electroporation enhancer (all from IDT) is described in detail for resDCM1.^31^ The edit for resLVNC was performed identically with the crRNA: 5´-CTCACCGGACTACGAGA CAG-3´ and the HDR-template: 5´GGTGTGAAGATTCTAAATCCTGCTCCTTGGCTCCC TCACAGATATGGCCCAGAAAGGCCGCGGTCTCGTAGTCCCGTCAGCCGGTCACTCTCCCCGAGGTCCCACACTCCCAGCTTCACCTCC-3´. Gene edit was performed on LVNC-iPSC at a passage < 30. The editing efficiency was 45% (32 positive clones of 72). The edit for resLVNC-634.W was performed with the crRNA: 5’-CGGTCTCGTAGTCCGGTGAG-3’ and the HDR-template: 5’-GGTGTGAAGATTCTAAATCCTGCTCCTTGGCTCCCTC.

AGATATGGCCCAGAAAGGCCGTGGTCTCGTAGTCCGGTGAGCCGGTCACTCTCCCCGGTCCCACACTCCCAGCTTCACCT-3’. The editing efficiency was 23.5% (17 positive clones of 30).

### Engineered Heart Muscle (EHM)

#### EHM generation

EHM was generated in accordance with previously published protocols with minor modifications.^60,63^ Briefly, 28 days old iPSC-CM of high purity were combined with human foreskin fibroblasts (HFF-1, ATCC, SCRC-1041) in a 70:30% ratio and resuspended in Iscove’s medium supplemented with 4% B27 minus insulin, MEM non-essential amino acids, 300 µM L-ascorbic acid 2-phosphate, 100 ng/mL IGF-1, 10 ng/mL FGF-2, 5 ng/mL VEGF_165_ and 5 ng/mL TGF-β1 (Peprotech). To prepare the collagen mixture required for tissue hydrogel formation, equal volumes of collagen (Collagen Solutions, FS22024) and RPMI 2x (Thermo Fisher Scientific, 51800) were mixed and neutralized with 0.1 N NaOH (Merck, 137031). The cell suspension was resuspended in an appropriate volume of the collagen mixture, with 180 µL of the subsequent hydrogel containing 0.5×10^6^ cells being gently pipetted in a ring-shape in each well of a custom 48 multi-well plate (myriamed GmbH, myrPlate Uniform, TM5-MED). For the first three days of tissue culture, the medium was supplemented with 5 ng/mL TGF-β1 and exchanged daily. For the following 11 days the EHM medium was exchanged daily, which was subsequently exchanged for an adapted EHM maturation medium (MEM α, Thermo Fisher Scientific, 22561021, 4% B27 minus insulin, MEM non-essential amino acids, 100 ng/mL IGF-1, 10 ng/mL FGF-2, 5 ng/mL VEGF_165_) for the remainder of the tissue culture period. For drug treatment, verapamil (30 nM) for added to the culture for 7 days. To monitor contractile activity by video-optical measurements of pole deflection, non-destructive imaging of the EHM plates was performed with a myrImager (myriamed GmbH).

#### Isometric force measurements

Measurements of contractile function were performed under isometric conditions in organ baths at 37 °C in gassed Tyrode´s solution (120 NaCl, 1 MgCl_2_, 0.2 CaCl_2_, 5.4 KCl, 22.6 NaHCO_3_, 4.2 NaH_2_PO_4_, 5.5 glucose, and 5.5 L-ascorbic acid; all in mM). EHM were electrically stimulated at 1.5 Hz with 5 ms square pulses of 150 mA and mechanically stretched at 62.5 µm intervals until reaching the maximum force amplitude. Responses to increasing extracellular calcium concentrations (0.2–4.0 mM) were investigated for each individual EHM. Ratios of active and passive force were obtained by comparing actively generated force with the resting tension of the EHM at saturating calcium levels. Measurements were performed on day 35 after EHM casting, corresponding to a total iPSC-CM age of approximately 60–63 days, as the tissues were cast on day 28 post-differentiation.

#### EHM digestion

EHM were enzymatically digested to isolate single cells for analysis of CM content according to previously published protocols.^60^ The tissues were incubated for 1 h in a collagenase 1 (Sigma Aldrich) solution at 37 °C, with the supernatant transferred into a conical tube. The loose EHM was further incubated in an Accutase (Thermo Fisher) solution supplemented with 0.025% Trypsin (Thermo Fisher) and 20 μg/mL DNase I (Calbiochem) for 30 min at RT. The EHM was gently resuspended to ensure cell separation, and the solution transferred to the tube containing the collagenase supernatant. The digestion dish was washed with double volume of PBS supplemented with 5% v/v FBS, 1% BSA, and 0.5% Triton X-100 (blocking buffer). The complete suspension was centrifuged at 100 × *g*, 10 min, 4 °C, and resuspended in a new volume of blocking buffer before counting using the Casy automated cell counter (OMNI Life Science). The suspension was centrifuged again with the same parameters, with subsequent fixation in 70% ice-cold ethanol.

### Gene analysis and Western Blot

The gene and protein analysis methods employed are standard procedures, and detailed information can be found in the Supplementary Materials. The gene expression via qPCR was performed with the BioRad system, RBM20 locus analysis by Sanger sequencing (Microsynth, Göttingen), and mRNA sequencing by the Illumina system.

### Karyotyping

Karyotyping was performed at Life & Brain (Bonn, Germany) via array-based genome-wide genotyping utilizing the Illumina BeadArray. Data was analyzed in GenomeStudio v. 2.0 (Illumina) with the cnvPartition 3.2.0 plug-in.

### Cell staining

Details on cell stainings and microscopy are provided in the supplements.

### Seahorse

For simultaneous measurements of oxygen consumption rate (OCR), iPSC-CM were digested onto a Geltrex-coated 96-well cell plate (Seahorse, Agilent Billerica, MA, USA) at a density of 5 × 10^4^ cells per well. The cells were cultivated for 5–7 days under standard conditions as described above. Baseline respiration was measured in Seahorse XF base medium supplemented with 1 mM pyruvate and 5.5 mM glucose after calibration at 37 °C in an incubator without CO_2_. Repeated measurements of OCR were performed over time. Metabolic states were measured after subsequent addition of 3 µM Oligomycin, 1 µM Carbonyl cyanide 4-(trifluoromethoxy) phenylhydrazone (FCCP), 3 µM Antimycin A, and 3 µM Rotenone. The data were normalized to protein amount using a BCA assay to correct for cell seeding differences. For drug treatments, either 30 nM verapamil, 5 µM metoprolol, or 1 µM AIP was added to the medium for 24 h.

### Data analysis

#### Sarcomere

The sarcomeric regularity was analysis with FFT transformation, using the first peak height as a measurement of regularity.^22^

#### Mitochondrial network

Cells were incubated with 250 nM MitoSpy orange (Biolegend) for 30 min prior to fixation. After image acquisition, the mitochondrial network was analyzed using the ImageJ plugin MiNA as described previously.^64^

#### Statistics

All statistical calculations were performed using GraphPad Prism 10.0. Statistical analyses were performed using appropriate tests depending on data distribution and experimental design. For comparisons between two or more groups, either a standard non-parametric *t*-test or ANOVA was applied. When multiple measurements originated from the same differentiation batch, nested statistical analyses were employed to account for hierarchical data structure. *P*-values are reported for every statistical test performed, regardless of significance, to ensure full transparency, except for the EHM curves that are marked with *p < 0.05, **p < 0.01, ***p < 0.001, and ****p < 0.0001. Details regarding sample sizes (*n*) and the type of statistical test used are provided in the figure legends for each graph.

## Supplementary information

Supplemental Material

Original Western Blot membranes

## Data Availability

Newly generated bulk RNA sequencing data were produced in this study. All sequencing datasets have been deposited in the Gene Expression Omnibus (GEO) under accession number GSE329393 and are publicly available.
