# Supplementary material for: RBM20 variants disrupt Ca2+ handling and metabolism in dilated and non-compaction cardiomyopathy stem cell models
Source: Signal Transduct Target Ther. 2026 Jul 14;11:276. doi: 10.1038/s41392-026-02838-7 (PMC13365226; doi:10.1038/s41392-026-02838-7)
Supplement: Supplementary file 1 — Supplemental Material [file 41392_2026_2838_MOESM1_ESM.docx]

Supplementary Materials for

**RNA-Binding-Motif 20-variants disrupt Ca^2+^-handling and metabolism in dilated and non-compaction cardiomyopathy stem cell models**

Sabine Rebs, Farbod Sedaghat-Hamedani, Elham Kayvanpour, Hanna Eberl, Branimir Berecic, Jan Dudek, Nicole Wagensohner, Aylin Seedorf, Julia K. Unsöld, Daniela Hübscher, Christoph Reich, Teresa Klein, Sören Doose, Viacheslav O Nikolaev, Tjark Buchwald, Ahmed Wagdi, Michael Kohlhaas, Nataliya Dybkova, Patricia M. Costa, Kaomei Guan, Gerd Hasenfuss, Eva A. Rog-Zielinska, Samuel Sossalla, Markus Sauer, Christoph Maack,^,^ Malte Tiburcy, Benjamin Meder^#^, Katrin Streckfuss-Bömeke ^#, *^

**Correspondence to:** [katrin.streckfuss-boemeke@uni-wuerzburg.de](mailto:katrin.streckfuss-boemeke@uni-wuerzburg.de)

**This PDF file includes:**

Materials and Methods

Fig. S1 to S7

Tables S1 to S9

Supplementary Text

**Material and methods**

**Cell staining**

***Fixation of the cells for IF and FLOW***

IPSC or iPSC-CM were plated onto Geltrex-coated coverslips or 8-chambered coverslip system (Cellvis). For fixation, the cells were washed once with 1xPBS and incubated 20 min with 4 % Histofix solution (Roth). After Histofix was discarded, the cells were washed with 1x PBS and 1-2 mL Blocking solution (1% BSA in 1x PBS) was added. The cells were stored in Blocking solution at 4 °C until usage (up to 2 months).

For FLOW, one well with iPSC-CM was detached using 0.25 % trypsin/EDTA for 10 min at 37 °C, stopped with FCS (1:1) and transferred to a FCS centrifugation tube (all centrifugation steps at 200 g for 5 min). Cell pellets were washed once in 1x PBS and subsequently stored in Blocking buffer at 4 °C for up to one month.

***Antibody stainings for IF and FLOW***

For IF stainings, primary and secondary antibodies were diluted in Staining solution (0.1 % triton-x in 1 % BSA/PBS). Dilutions and all antibodies used are listed in Supplementary table 3. The secondary antibody dilutions ranged between 1:500 to 1:1000.

For FLOW- cTNT staining, the cells were incubated over night at 4 °C with the primary antibody cTNT in a 1:500 dilution in FACS buffer (1 % BSA/PBS with 0.1 % Triton-X). The next day, the cells were washed thrice with FACS buffer and subsequently incubated with the secondary antibody in a 1:1000 dilution in FACS buffer (Supplementary table 2). The cells were washed thrice again in FACS buffer und diluted in 200 µL 1x PBS for measurements using a FACS Canto II with the following settings: 10000 events, forward scatter 228 V, side scatter 440 V, 488 Alexa Fluor laser 390 V. The detection threshold was set according to the blank sample and cTNT positive cells were presented as cTNT positive cells in [%]. Cardiac differentiations with >85% positive for cardiac troponin T were used for further analysis.

For CM analysis of dissolved EHM, the fixed cells were centrifuged at 300 g, 5 min, 4° C and the ethanol supernatant aspirated. The cell pellet was resuspended in 0.5 mL blocking buffer and distributed into a U-shaped 96-multi well plate with one well used for an unstained IgG control and a second well for the antibody staining for each EHM sample. The antibody staining was sequentially performed in blocking buffer with an unspecific IgG or sarcomeric α-actinin primary antibody, followed by a double wash with PBS^-/-^ and a secondary antibody staining with anti-IgG and Hoechst 33342, each at RT for 1 h. The cell populations were analysed for viability and singularization, then assessed for the stained protein of interest using the BD LSR II flow cytometer and the FACSDiva software (BD Biosciences).

**Gene analysis**

***RNA Isolation and cDNA synthesis***

Total RNA was isolated as described in the SV Total RNA Isolation System (Promega) according to the manufacturer’s instructions for “RNA Isolation from fibrous tissue”. The DNase-digestion step was expanded to 30 min at RT. 100-300 ng of RNA was used in first-strand cDNA synthesis using the iScript cDNA Synthesis Kit (BioRad).

***Quantitative PCR***

Quantitative PCR was performed using 1x iQ™ SYBR® Green Supermix (Bio-Rad) with 5 ng cDNA and 500 nM primers (each forward and reverse) and run on the CFX real-time PCR detection system (Bio-Rad). Primers used are listed in Supplementary table 4.

**Sequencing**

***Sanger Sequencing***

To sequence the RBM20-exon 9 locus, gDNA was isolated using the QIAamp DNA Mini Kit (Qiagen) following the manufacturer´s instructions. The gDNA was measured using a Nanodrop and adjusted to a value between 100 – 200 ng/µL. To amplify the exon 9 locus, a PCR was conducted with 2 µL cDNA in a PCR mix consisting of 1 µL forward and reverse primer (10 µM, Supplementary table 3), 5 µL Green buffer (5x) (Promega), 1.6 µL dNTP mix (10 mM) (Bioline), 0.1 µL Tag-polymerase (Promega) and 14.3 µL water (end volume 25 µL). The PCR was rum with 56 °C annealing temperature with 30 cycles. The PCR product was sent for Sanger sequencing to Mircosynth Seqlab (Göttingen).

***Illumina Sequencing***

RNA sequencing using poly(A) enrichment of the mRNA was performed for a total of 16 samples, comprising 3 LVNC, 5 resLVNC, 3 DCM1, and 5 resDCM1 samples. A median read depth of 76.52 million was achieved per pair-ended sequencing data, with a read length of 75 base pair. The reads were subjected to a quality check using the tool fastp ^1,2^ The reads were then aligned to human genome GRCh38 using the tool Subread ^3,4^ using the splice-aware option. BAM files were then used as an input to the *featureCounts* tool of the Subread package, to count the number of reads aligning to the gene features as defined in NCBI RefSeq annotation for hg38 (build 38.2). For the counting, only uniquely mapping fragments were considered. Read counts were imported in R package *DESeq2* ^5^ for normalization of sequencing depth and analysis of the differentially expressed genes between groups. Wald tests were used to calculate log2 fold changes and associated p-values, which were adjusted for multiple testing using the Benjamini-Hochberg procedure. Differentially expressed genes with an adjusted p-value < 0.05 were considered significant. DEXSeq was used for the analysis of differential exon usage, focusing on variations in exon-level expression patterns between groups. The exon counts were normalized, and generalized linear models (GLMs) were employed to test for significant differences in exon usage across conditions ^6^. To account for hidden batch effects, Surrogate Variable Analysis (SVA) was performed. The normalized counts for a total of 28,395 genes are visualized as a volcano plot. Gene Ontology (GO) term enrichment analysis was performed on the differentially expressed genes identified through DESeq2 using Cytoscape with ClueGo plugin (settings: Ontologie KEGG, pathway with p-value < 0.05).

**Karyotyping**

Karyotyping was performed at Life & Brain (Bonn, Germany) via array-based genome-wide genotyping utilizing the Illumina BeadArray. Data was analyzed in GenomeStudio v2.0 (Illumina) with the cnvPartition 3.2.0 plug-in.

**Western Blot**

***Protein lysis***

Cell pellets were lysed in lysis buffer (20 mM Tris-HCl [pH 7.4], 200 mM NaCl, 20 mM NaF, 1 % Igepal, 1mM Na_3_VO_4_, 1mM DTT, 1x PhosStop, 1x cOmplete, EDTA-free protease inhibitor). The solution was incubated 30 min on ice, and subsequently centrifuged and the supernatant collected. Protein concentration was determined with the Pierce BCA protein assay kit according to manufactrurer`s instructions.

***SDS-electrophoresis, blotting and imaging***

20 µg protein were denaturated in Laemmli buffer at 37 °C for 30 min and separated in SDS-polyacrylamide gels (4-15 % Mini-Protean precast Gel; BioRad) with constant 15-20 mA/gel. Next, the proteins were transferred to either 0.45 µm nitrocellulose membrane (RBM20) or methanol activated 0.45 µm PVDF (PLN, PLN-S16p, PLN-T17p) membranes using a semi-dry transfer system (BioRad). Membranes were blocked in 5 % milk or 5 % BSA depending on the antibody (Supplementary table 2). Primary antibodies were diluted in 1 % milk or 1 % BSA (Supplementary table 2) and incubated over night at 4 °C. HRP-coupled secondary antibodies (Supplementary table 2) were incubated for 1 h at room temperature. For imaging with the ChemiDoc XRS+ imaging system (BioRad), the membranes were incubated for 5 min with Immobiolon chemiluminescent HRP substrate (Millipore) and imaged with increasing exposure time.

**Microscopy**

For morphology and ALP stainings, the Axio Observer. A1 (Zeiss) was used. Immunofluorescence stainings were imaged with Axio Observer. Z1 (Zeiss) or the LAS X THUNDER (Leica). For live cell imaging of FURA-2-AM calcium imaging, an Olympus Motic AE31 microscope was used with an IonOptix epifluorescence acquisition system. For live cell imaging of Fluo-4-AM calcium imaging, the LSM710 confocal system (Zeiss) was used. For live cell imaging of TMRM/Mitotracker measurements the LAS X SP5 (Leica) was used.

***dSTORM***

7000 iPSC-CM were plated onto an eight-well chamber coverslip system (Cellvis). Cells were fixated 7 days later and stained with 1:500 RBM20 primary antibody and 1:500 Alexa-647 secondary antibody and a nucleus staining (DAPI). Instead of mounting solution, 500 µL 1x PBS was added to each well. The dSTORM imaging was performed using an Olympus IX-71 inverted microscope equipped with an APON 60xOTIRF (NA 1.49) oil immersion objective and a nosepiece stage (IX2-NPS, Olympus). Alexa Fluor 647 was excited using a 639 nm laser (Genesis MX639-1000, Coherent) at an intensity of approximately 2 kW/cm^2^ to induce photoswitching. The imaging buffer consisted of PBS supplemented with 100 mM cysteamine hydrochloride (Sigma-Aldrich), pH 7.4, 4 % glucose, 8 units/ml glucose oxidase and 160 units/ml catalase. The excitation light was filtered through a 642/10 nm bandpass filter (Semrock) and focused on the back focal plane of the objective. A lens system and mirror, positioned on a linear translation stage, enabled switching between different illumination modes (EPI, HILO, and TIRF). Fluorescence emission was collected by the same objective and transmitted through a beam splitter (FF410/504/582/669-Di01, Semrock). The emitted light was further filtered using a bandpass filter (Brightline HC 679/41, Semrock) and projected onto an electron-multiplying CCD camera (iXon Ultra 897, Andor). Additional lenses in the detection path resulted in a final pixel size of 128.9 nm. A total of 15,000 frames were acquired at a frame rate of 67 Hz (15 ms exposure time). Image reconstruction was performed using rapi*d*STORM 3.3 ^7^, applying a threshold to exclude signals with fewer than 700 photons from the localization data. Further analysis was conducted using the software LOCAN ^8^. To investigate RBM20 density within the nucleus, a region of interest (ROI) encompassing the nucleus was selected for each *d*STORM image. For analyzing RBM20 in the cytoplasm, an extended region around the nuclear ROI, with a distance of 2 µm, was chosen. For cluster analysis the localizations within these ROIs were grouped using a DBSCAN (Density-Based Spatial Clustering of Applications with Noise) ^9^ algorithm with parameters set to ε = 20 and minPoints = 3 ^10^. A cluster was defined as a group of localizations with a minimum of three localizations, originating from one or a few secondary antibodies. Cluster densities in the nucleus and cytoplasm, as well as the ratio of these two densities (nucleus density : cytoplasm density) per cell were calculated.

***Electron microscopy***

3D spheroids were washed once with 1× PBS and fixed overnight at 4 °C in isosmotic Karnovsky’s fixative. On the following day, the fixative was replaced with 0.1 M cacodylate buffer (pH 7.4). Samples were embedded in 4% low melting agarose then stained with 1% osmium tetroxide and 1% uranyl acetate, dehydrated through a graded ethanol series, followed by acetone, and embedded in Durcupan resin using a laboratory microwave. Ultrathin sections (70 nm) were mounted on copper grids, post-stained with 3% lead citrate, and imaged using a Talos L120C transmission electron microscope (Thermo Scientific) operated at 120 kV and equipped with a 4 K × 4 K Ceta CMOS camera (Thermo Scientific). The analysis and visualization were performed in IMOD software.

**Live cell imaging**

***Calcium kinetics with Fluo-4-AM***

2.5 – 3 x 10^5^ iPSC-CM were plated onto Geltrex-coated 24 mm round coverslips and imaged 7-9 days later. Cells were loaded with 1.5 mL Tyrode solution (140 mM NaCl, 5.4 mM KCl, 1 mM MgCl_2_, 1.8 mM CaCl_2_, 10 mM Na-HEPES, and 10 mM glucose, pH=7.4) containing 2,5 µM Fluo-4-AM and 0.025 % Pluronic (both from Thermo Fischer Scientific). Image acquisition was performed at RT with a Zeiss LSM 710 confocal microscope and a 63x 1.4 oil objective in line scan mode (512 pixels, 45.5 µm (zoom factor 3), 1057.7 Hz, 12 bit and 20,000 cycles at maximal speed). The cells were continually paced at 0.25 Hz by placing a customized electrode into the measuring set-up. Calcium analysis was done as described by us previously for calcium kinetics and spark analysis with ImageJ Sparkmaster plugin ^11^. For the spark analysis the 3D (width*height*amplitude) sum of a defined area during diastole was calculated. Drug treatments were conducted by directly diluting the compound into the measuring solution with the appropriate concentrations of Iso (1 µM), verapamil (30 nM) and metoprolol (5 µM). The drugs were incubated for 15 min before the subsequent measurement.

***Calcium content with FURA-2-AM***

3 – 4x10^4^ iPSC-CMs were digested onto Geltrex – coated 35 mm round Fluoro dishes (World Precision Instruments) and imaged 7-9 days later. Cells were loaded with 5 µM FURA-2-AM dye was dissolved in 1 ml Tyrode solution (140 mM NaCl, 5.4 mM KCl, 1 mM MgCl_2_, 1.25 mM CaCl_2_, 10 mM Na-HEPES, and 10 mM glucose, pH=7.4) for 15 min at 37 °C followed by incubation with Tyrode solution for 15 min at 37 °C. Finally, the Tyrode solution was refreshed before measuring the cells at RT. The FURA-2 loaded cells were excited at 340 and 380 nm and the emissions were measured at 510 nm. Data is shown as ratios of 340/380 nm with subtracted background values. For every dish, 3-4 single cells were measured by recording Ca^2+^ transients with increased pacing: 0.25, 0.5, and 1.0 Hz. The parameters were analyzed using the IonOptix software. For AIP treatment, 1 µM AIP was directly diluted into the measuring solution after the basal measurements and incubated for 15 min.

***Mitochondrial calcium with mtPericam***

For mitochondrial calcium measurements, 3x10^5^ iPSC-CM were seeded on 35 mm dishes (MatTek) and transfected 5 days later with ratiometric Pericam Mt3.1 (mtPericam) ^12^ with the Lipofectamine LTX kit (Thermo Fisher) mixing 2 µg mtPericam-plasmid, 300 µL OptiMEM, 12 µL lipofectamine LTX reagent and 1 µg lipofectamine PLUS reagent. This mixture was incubated 5 min at RT and subsequently added to the iPSC-CM in 1.7 mL Cardio Culture medium supplemented with 1x Pen/strep. After 6 h, at 37 °C medium was exchanged for Cardio Culture medium. 48 h after transfection, the iPSC-CM were imaged using the SP8 confocal microscope (Leica). To conduct measurements, the medium was exchanged to Tyrode solution (140 mM NaCl, 5.4 mM KCl, 1 mM MgCl2, 1.8 mM CaCl2, 10 mM Na-HEPES, and 10 mM glucose, pH=7.4) and the cells were paced at 0.5 Hz. Ca^2+^-bound and Ca^2+^-unbound mtPericam was visualized with excitations of 488 nm (14%) and 405 nm (8%), respectively, in a sequential scan and emissions were captured with photomultiplier tubes set to 500-550 nm. Images were analyzed using ImageJ determining the mean pixel intensity for each picture. Afterwards, the ratio for the mean intensities were determined for Ca^2+^-bound divided by the Ca^2+^-unbound values.

***TMRM measurement***

2.5 – 3 x 10^5^ iPSC-CM were plated onto Geltrex-coated 24 mm round coverslips and imaged 7-9 days later. Cells were stained with 2.5 nM TMRM (Thermo Fisher Scientific) and 100 nM Mitotracker Green (Thermo Fisher Scientific) in Cardio Culture medium for 1 h at 37 °C. After 1 h , the solution was replaced with Cardio culture medium with 2.5 nM TMRM and images were acquired on the confocal system SP5 from Leica. For analysis, the mean value pixel intensity for the TMRM and Mitotracker channel were calculated using ImageJ and subsequently the ratio of TMRM/Mitotracker was used for quantification.

***Contractility measurements***

Contractility measurements were performed using the IonOptix Calcium and Contractility System in combination with an inverted microscope equipped with a 40x objective. Contractility as a parameter for systolic function was quantified with pixel intensity and pixel correlation algorithms, which detect changes in image intensity patterns (from a brightfield image) within a defined region of interest over time relative to a reference frame. Seven days prior to the experiments, 3 – 5 spheroids were digested and seeded onto Geltrex-coated 35 mm dishes (MatTek). Measurements were recorded in IonWizard 6.3 software at 34 – 36 °C, analyzing 10-20 cells at a pacing frequency of 0.5 Hz. Cells were sequentially perfused with Tyrode solutions (140 mM NaCl, 5.4 mM KCl, 1 mM MgCl_2_, 10 mM HEPES, 10 mM glucose, pH=7.4) containing increasing CaCl_2_ concentrations (0.6 mM, 1.2 mM and 1.8 mM) with 5 min intervals between each step to ensure complete solution exchange and contractility was recorded for 17 s per cell. Data analysis to calculate the contractility peak height was performed using CytoSolver 2.2.2 and Microsoft Excel.


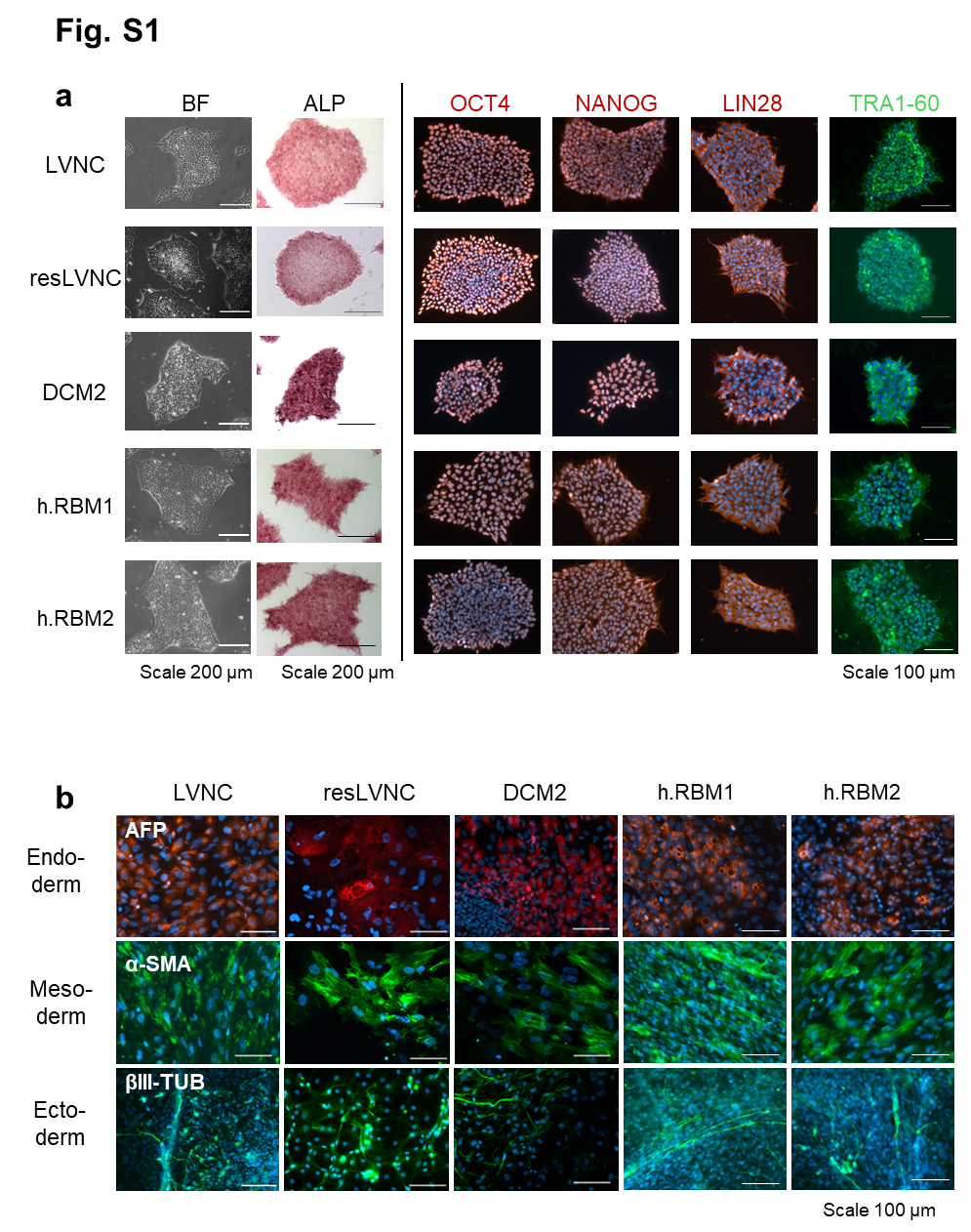


**Fig. S1: All iPSC lines show full pluripotency.**

Generated iPSC lines from LVNC-family: LVNC II.3 and from the DCM family: DCM2 III.10, h.RBM1 II.3 and h.RBM2 III.3. h = healthy

**a:** Brightfield and immunofluorescence stainings of iPSC. All iPSC lines are positive for stem cell morphology, ALP activity, and stem cell marker expression OCT4, NANOG, LIN28, and TRA1-60. Brightness and contrast have been enhanced. BF: Brightfield; ALP: alkaline phosphatase.

**b:** All iPSC lines differentiate into cells from all three germ layers stained with the appropriate antibodies against endoderm (AFP: alpha-1-fetoprotein), mesoderm (α-SMA: alpha smooth muscle) and ectoderm (βIII-TUB: tubulin beta 3 class III). Brightness and contrast have been enhanced.


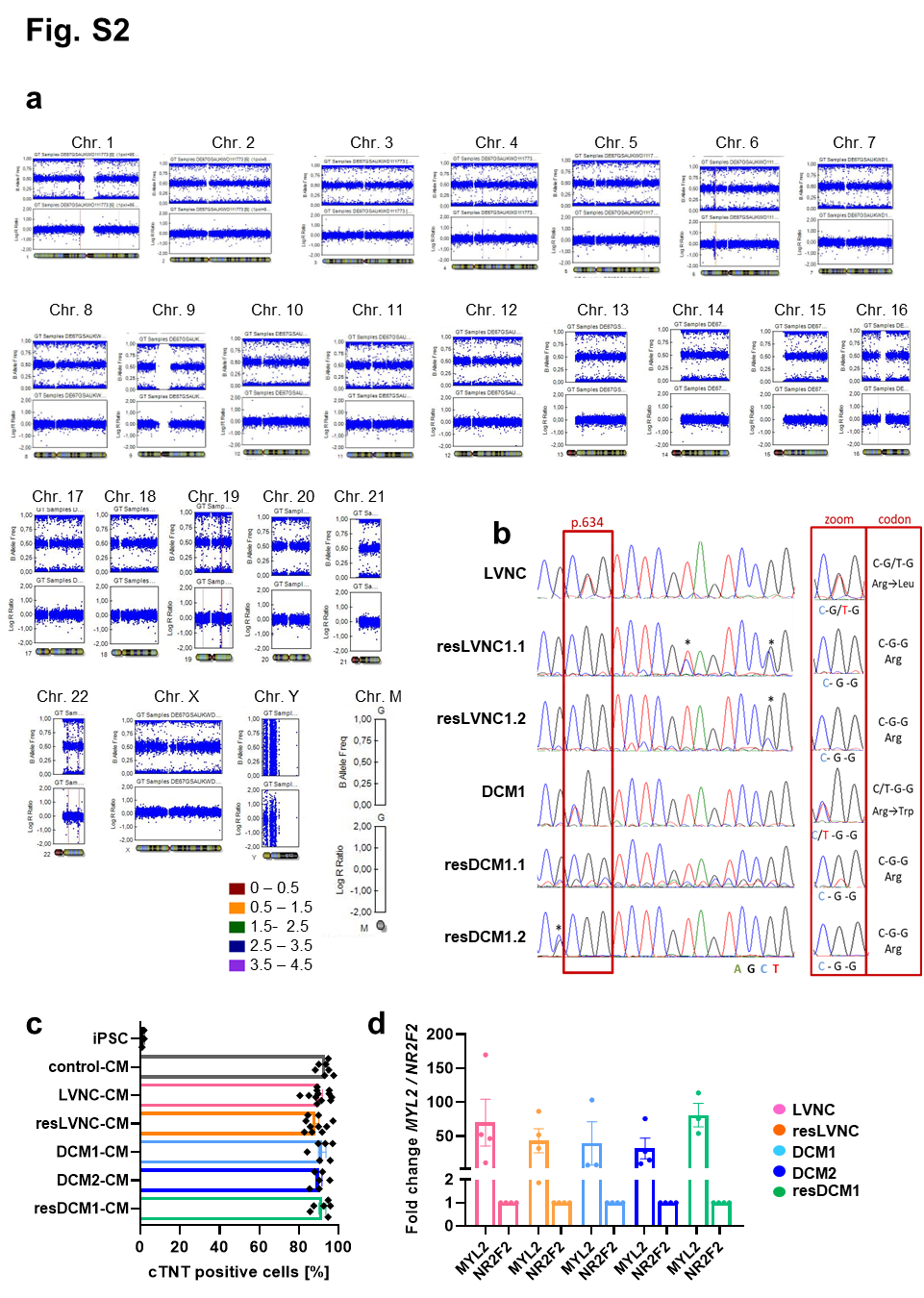


**Fig. S2: Genomic integrity and differentiation into iPSC-CM of LVNC- and DCM-affected family members with RBM20 mutations.**

**a:** All iPSC lines retain genetic integrity and a normal karyotype (XX or XY, 46). Exemplary karyotype shown for resLVNC iPSC (XX, 46). Graphs show B Allele Frequency and Log R Ratio for each chromosome. Chr. = chromosome

**b:** Respective Sanger sequencing results of the RBM20 locus exon 9. The zoom visualizes position c.1900-1902, which encodes the amino acid number 634 within the RBM20 gene. Wt-RBM20 encodes C-G-G for p.R634 as an arginine (Arg), whereas LVNC harbors a heterozygous missense mutation C-G/T-G leading to p.R634L (leucine (Leu)) and DCM with C/T-G-G leading to p.R634W (tryptophane (Trp)).

**c:** FLOW cytometry analysis of 60-90 days-old patient and control iPSC-CM stained with cardiac Troponin T (cTNT) antibody. Each dot represents one cardiac differentiation. Undifferentiated iPSC served as negative control.

**d:** QPCR analysis of ventricular marker *MYL2* and atrial marker *NR2F2*. Both genes were normalized to the housekeeper *18s* and subsequently normalized to NR2F2 as a value of one to show the fold change of *MYL2* in relation to the atrial *NR2F2* in every sample. Each dot represents one cardiac differentiation.


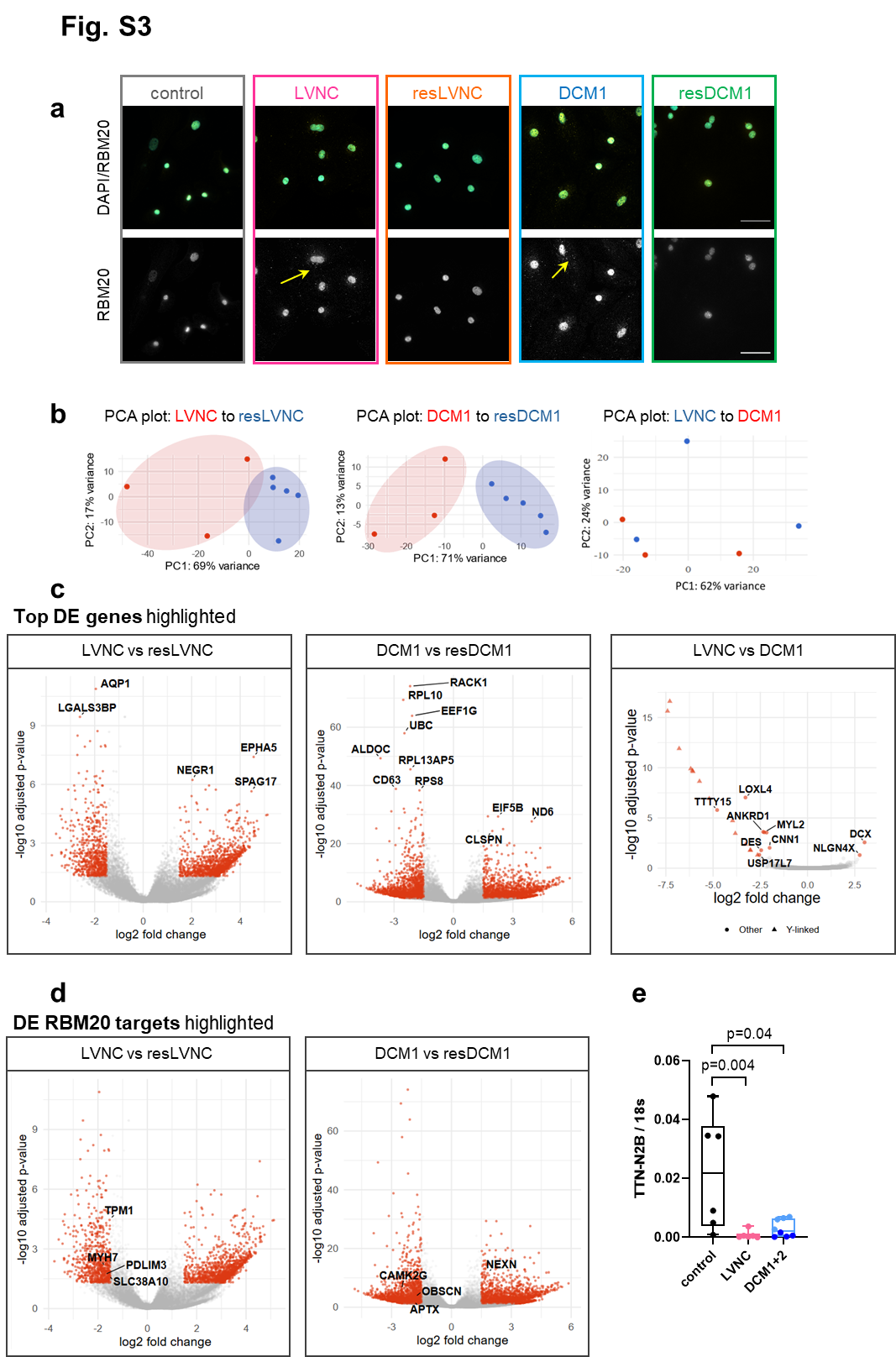
**Fig. S3: RBM20-dependent mis-localization and *TTN* splicing.**

**a:** RBM20 accumulates in the cytoplasm. Representative immunofluorescence stainings of control-, LVNC-, resLVNC-, DCM1- and resDCM1-CM. Yellow arrows marks exemplary RBM20 cytoplasmatic localization. Antibody staining against RBM20 (yellow) with counterstaining of the nucleus (cyan). Scale bars: 50 µm.

**b:** The figure shows Principal Component Analysis (PCA) plots generated before differential expression analysis that highlights clusters as expected (patient vs rescue). The PCA plot for LVNC vs DCM1 shows some overlap between the two groups. This suggests that the gene expression profiles of LVNC and DCM1 are more similar to each other than compared to their isogenic controls.

**c, d:** Differential gene expression analysis:

**c:** Volcano plots showing DE genes with key markers labeled in comparison of LVNC vs resLVNC, DCM1 vs resDCM1, and LVNC vs DCM. The -log10 adjusted p-value is plotted against the log2 fold change for each comparison.

**d:** Volcano plots highlighting DE genes that are targets of RBM20 in the same comparison as shown in c. Specific RBM20 target genes are labeled.

DE = Differential (gene) expression, vs = versus

**e:** QPCR analysis of RBM20 splice target *TTN* as *N2B* isoform normalized to *18s*. Data is shown as box plots, whereas every dot represents one differentiation experiment. P-values by Mann-Whitney test Volcano plots highlighting DE genes that are targets of RBM20 in the same comparison as shown in D. Specific RBM20 target genes are labeled.

DE = Differential (gene) expression, vs = versus


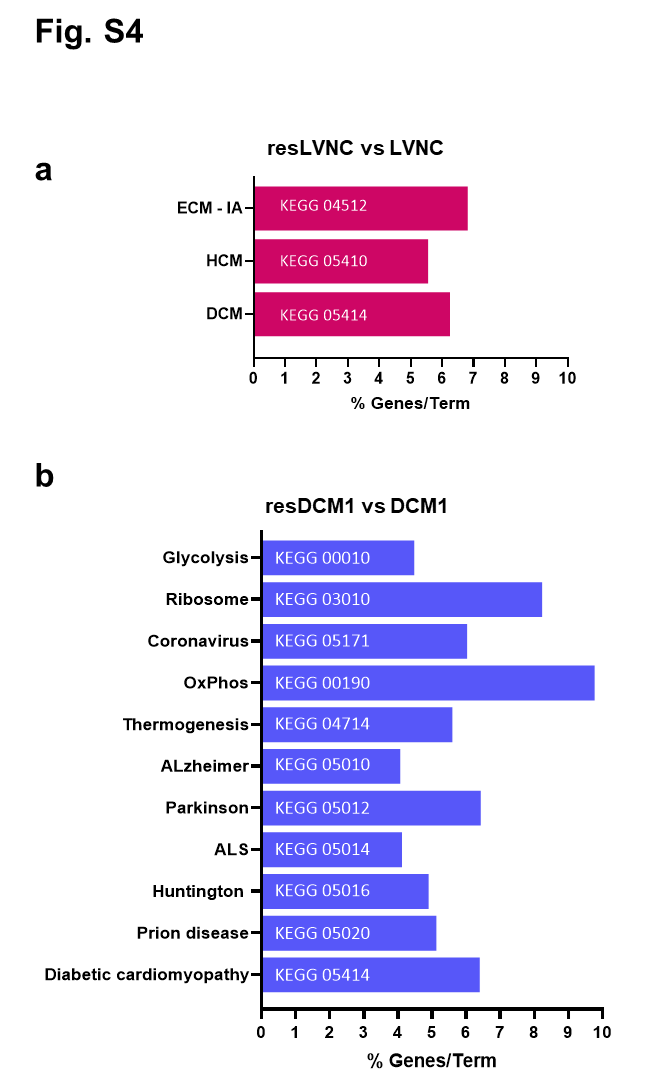
**Fig. S4: KEGG GO-term hits (pV<0.05) for the Top100 differentially expressed genes.**

**a:** ResLVNC vs LVNC.

**b:** ResDCM1 vs DCM1.

IA: interaction, HCM: hypertrophic cardiomyopathy, DCM: dilated cardiomyopathy, OxPhos: oxidative phosphorylation, ALS: amyotrophic lateral sclerosis


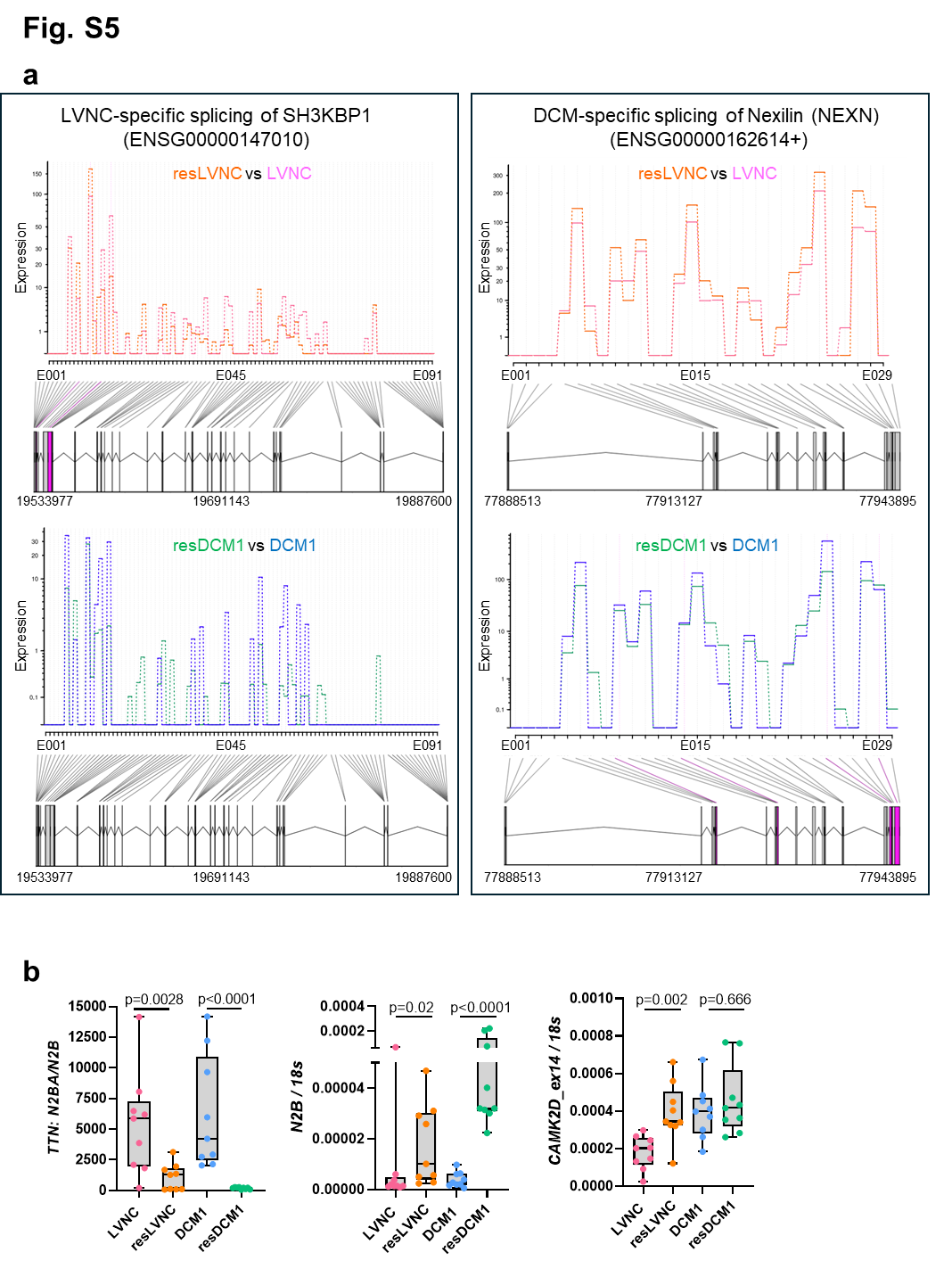


**Fig. S5: Splice graphs with exonic bin usage of LVNC and DCM-specific targets.**

**a:** Graphs represent the differential exon expression profiles. The y-axis represents normalized read counts of exons (exon usage), and the x-axis shows individual exons within a gene. The lower panels show the gene structure, with the bars below the x -axis representing exons, and the lines between the bars representing introns. The numbers at the bottom are genomic locations of the gene. Purple bars mark exons with significantly altered usage (FDR-adjusted p-value < 0.05) in the DEX analysis of isogenic versus patient cell lines.

**b:** Analysis of *TTN* and *CAMK2D* splicing in 3D-sheroid matured iPSC-CM. *TTN* splicing for the isoforms N2B and N2BA and *CAMK2D* splicing for exon 14. Data is shown as box plots, where each dot represents independently cultured spheroids. P-values are determined with Mann-Whitney test LVNC vs resLVNC and DCM1 vs resDCM1.


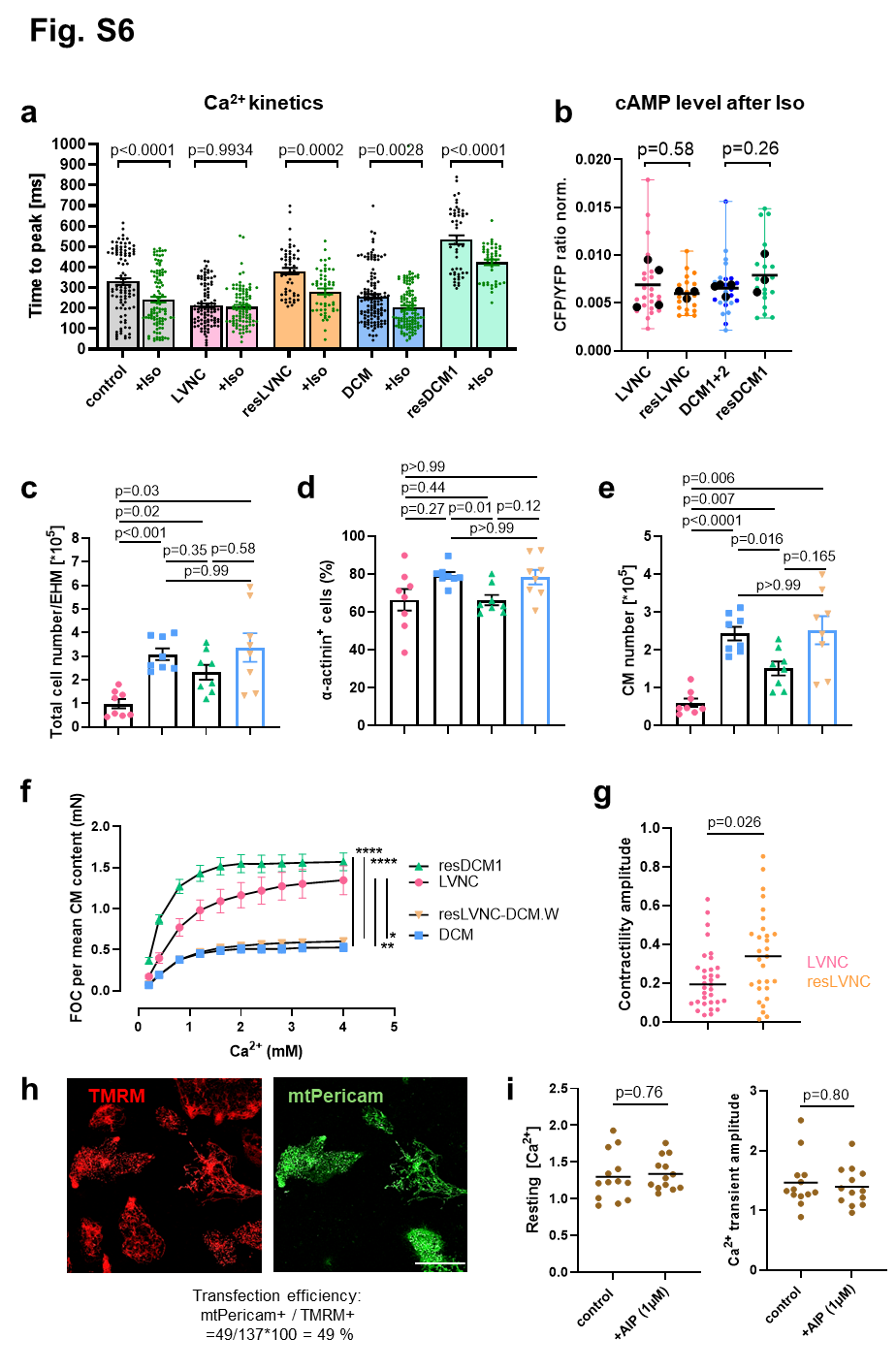


**Fig. S6: LVNC- and DCM-CM show differential Ca^2+^ handling pathologies.**

**a:** Ca^2+^ transient rise time (time to peak) time at basal (untreated) condition and after 15 min Isoprenaline (Iso, 1 µM) treatment with Fluo-4-AM. LVNC-CM showed decreased basal rise time levels and no measurable reaction to Iso. Data is presented as bar graph with mean ± SEM showing each measured cell as a single dot with [number of differentiations/analyzed cells] for basal: control [6/94], LVNC [6/101], resLVNC [3/55], DCM [7/126] and resDCM1 [3/51] and Iso stimulated: control [6/101], LVNC [6/97], resLVNC [3/53], DCM [7/119] and resDCM1 [3/44]. P-values by Two-way ANOVA Sidak´s multiple comparison test Basal vs Iso.

**b:** cAMP changes after Iso treatment among the iPSC-CM lines. Quantification of maximal FRET response to IBMX after Isoprenaline (Iso) stimulation. P-value was calculated by nested t-test.

**c-e:** Number of cells from digested EHM following organ bath measurements: [number of differentiations/analyzed EHM] for LVNC [3/8], DCM1 [3/8], resDCM1 [3/8], and resLVNC-DCM1.W [3/8]. P-value by Brown-Forsythe and Welch ANOVA with Dunnett´s multiple comparisons test.

**c:** Total cell number per EHM measured using Casy cell counter. LVNC-EHM show decreased total and iPSC-CM (CM) number compared to all other analyzed EHM.

**d:** Alpha-actinin-positive cells (%) after digestion of EHM measured via Flow Cytometry.

**e:** Proportion of CM calculated by total cell number and a-actinin positive cells**.**

**f:** FOC per CM normalized to viable iPSC-CM. The resDCM1- and LVNC-EHM show increased FOC compared to DCM1 and resLVNC-DCM.W line. P-values were calculated by Two-way RM ANOVA with Geisser-Greenhouse correction. P-value (column factor) for resDCM1 vs DCM1; resDCM1 vs resLVNC-DCM.W; LVNC vs resLVNC-DCM.W; LVNC vs DCM (significant values are marked * p<0.05, ** p<0.01, ***p<0.001) and LVNC vs resDCM1; DCM1 vs resLVNC-DCM.W (not significant). 8 EHM were analyzed for each line.

**g:** Contractility measurements using microscopy with edge-detection algorithm in LVNC- and resLVNC-spheroid isolated iPSC-CM. As a parameter for systolic function, the contractility peak amplitude was calculated. Data is presented as scatter plot showing single cell measurements from n=2 spheroid-experiments for LVNC [n=33] and resLVNC [n=24]. P-value was determined by Mann Whitney test.

**h:** Transfection efficiency for mtPericam. mtPericam was transfected into iPSC-CM and measured 2-4 days afterwards. For transfection efficiency and localization verification iPSC-CM were counterstained with TMRM. Brightness and contrast has been enhanced for visualization purposes. Scale: 50 µm

**i:** Treatment of control-CM with a CAMK2D-inhibitor (AIP) and its effect on diastolic/resting Ca^2+^ and Ca^2+^ transient amplitude. After basal measurements, cells were treated with AIP (1 µM) for 15 min. Data shows all single cell measurements (n=13) as scatter plot with mean. P-values were calculated by Mann Whitney test.


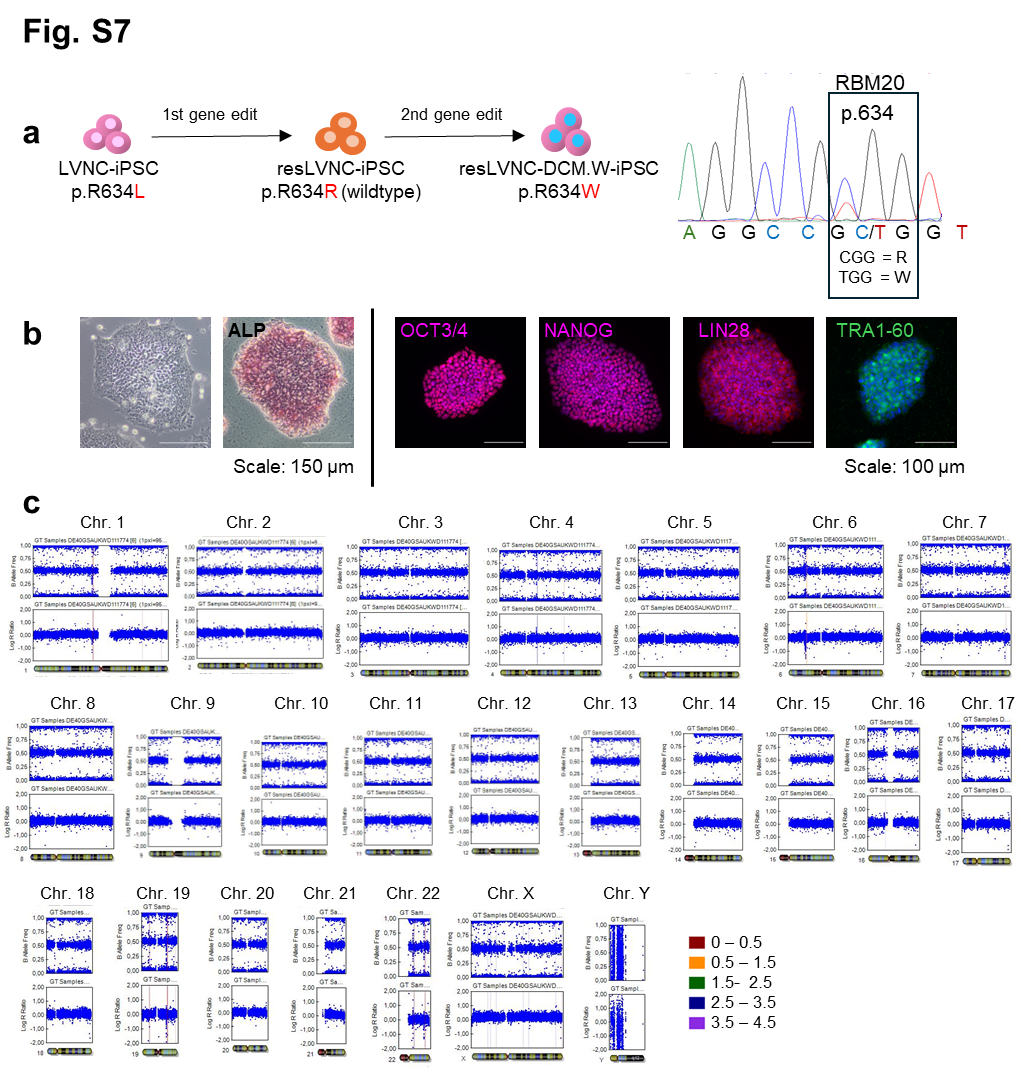


**Fig. S7: Characterization of resLVNC-DCM.W-iPSC.**

**a:** The resLVNC-DCM.W iPSC line is the LVNC patient background with the DCM mutation introduced.

**b:** Brightfield and immunofluorescence stainings of iPSC. The resLVNC-DCM.W-line is positive for stem cell morphology, ALP activity and stem cell markers OCT4, NANOG, LIN28 and TRA1-60. Brightness and contrast has been enhanced. BF: Brightfield; ALP: alkaline phosphatase.

**c:** Molecular karyotyping of resLVNC-DCM.W iPSC showing B Allele Frequency and Log R Ratio for each chromosome. Cells retain a normal karyotype (46, XX). Chr. = chromosome

**Supplemental tables**

**Table S1: Patient data from the latest follow-up visit**

|  | **LVNC** | **DCM1** | **DCM2** |
| --- | --- | --- | --- |
| **Age at last visit** | **69** | **57** | **53** |
| **Gender** | **female** | **male** | **male** |
| **Age of diagnosis** | **39** | **43** | **38** |
| **NYHA** | **II** | **I** | **I** |
| **hsTnT** | **9** | **7** | **5** |
| **Nt-ProBNP** | **65** | **95** | **50** |
| **GFR** | **55** | **74** | **106** |
| **Hypertension** | **No** | **No** | **No** |
| **Hyerlipidemia** | **Yes** | **Yes** | **No** |
| **DM-II** | **No** | **Yes** | **No** |
| **Smoking** | **No** | **No** | **No** |
| **Coronary artery disease** | **No** | **No** | **No** |
| **atrial fibrillation** | **No** | **No** | **No** |
| **Ventricular tachycardia** | **Yes** | **Yes** | **No** |
| **LGE in MRI** | **Yes** | **Yes** | **Yes** |
| **LVEF in MRI** | **46** | **48** | **48** |
| **ACE/AT1** | **Yes** | **Yes** | **Yes** |
| **MRA** | **Yes** | **Yes** | **Yes** |
| **Betablocker** | **Yes** | **Yes** | **Yes** |
| **SGLT2-inhibitor** | **No** | **Yes** | **No** |
| **ICD** | **Yes (55years old)** | **No** | **No** |

**Table S2: Summary of generated iPSC-lines from donor samples.**

| Patient donor | sample | Reprogramming | Intern-ID of clones |
| --- | --- | --- | --- |
| Healthy-1 from DCM II.3 | Skin/Fibroblasts | Sendai | S3RBM7  S3RBM8 |
| Healthy -2 from DCM III.3 | Skin/Fibroblasts | Sendai | S4RBM2  S4RBM6 |
| LVNC: II.3 | Skin/Fibroblasts | Plasmids | P6RBM1  P6RBM2 |
| DCM1: III.8 | Blood/PMBC | Sendai | S9RBM2  S9RBM4 |
| DCM2: III.10 | Skin/Fibroblasts | Sendai | S10RBM3  S10RBM5 |
| ResLVNC1 | Rescue in P6RBM1 | - | 6cr11  6cr20  6cr21  6cr29 |
| ResDCM1 | Rescue in S9RBM2 | - | 9cr35  9cr49 |
| LVNC-DCM.W | 634W mutation into 6cr29 | - | 6cr634W_clon20 |

**Table S3: List of antibodies used in this study.**

| Marker | Antibody | Dilution | ID and company |
| --- | --- | --- | --- |
| Pluripotency marker | IF: goat anti-OCT4 | 1:40 | R and D; AF1759 RRID AB_354975 |
|  | IF: mouse anti-SOX2 | 1:200 | R and D; MAB2018 RRID AB_358009 |
|  | IF: goat anti-LIN28 | 1:300 | R and D; AF3757 RRID AB_2234537 |
|  | IF: goat anti-NANOG | 1:50 | R and D; AF1997 RRID AB_355097 |
|  | IF: mouse anti-TRA1-60 | 1:200 | Abcam, ab16288 RRID AB_778563 |
|  | IF: mouse anti-SSEA4 | 1:200 | Abcam, ab16287 RRID AB_778073 |
| Germlayer marker | IF: rabbit anti-AFP | 1:500 | Dako, A0008 RRID AB_2650473 |
|  | IF: mouse anti-α-SMA | 1:3000 | Sigma, A2547 RRID AB_476701 |
|  | IF: mouse anti-β-III-TUBULIN | 1:2000 | Covance, MMS-435P RRID AB_2313773 |
| Sarcomeric marker | IF: mouse anti-α-actinin | 1:1000 | Sigma, A7811 RRID: AB_476766 |
|  | IF: rabbit anti-Titin | 1:750 | MyoMedix, TTN-9 RRID:AB_2734750 |
|  | FLOW: mouse anti-cTNT | 1:500 | Thermo Fisher, MS295PABX  RRID:AB_61810 |
| Cardiac function | IF: rabbit anti-RBM20 | 1:500 | Myomedix, RBM20-1 |
|  | Western: mouse anti-PLN | 1:5000  in milk | Thermo Fisher, MA3-922  RRID:AB_2252716 |
|  | Western: rabbit anti-PLN-S16p | 1:5000  in milk | Badrilla, A010-12AP  RRID:AB_2617047 |
|  | Western: rabbit anti-PLN-T17p | 1:5000  in milk | Badrilla, A010-13  RRID:AB_2617048 |
|  | Western: rabbit anti-RBM20 | 1:750  In BSA | Myomedix, RBM20-1 |
| Secondary antibody | IF: AF488 donkey anti-rabbit IgG | 1:1000 | Invitrogen, A31572 RRID AB_162543 |
|  | IF: AF555 donkey anti-goat IgG | 1:1000 | Invitrogen, A21432 RRID AB_141788 |
|  | IF: AF647 donkey anti-rabbit | 1:500 | Invitrogen, A31573  RRID:AB_2536183 |
|  | IF and FLOW: AF488 donkey anti-mouse IgG | 1:1000 | Invitrogen, A21202 RRID AB_141607 |
|  | IF: Cy3 goat anti-mouse IgG + IgM | 1:300 | Jackson Immuno, 115–165-068 RRID AB_2338686 |
|  | Western:  ECL Mouse IgG HRP-Linked 1ml | 1:10000 | Th. Geyer, NA931  RRID:AB_772210 |
|  | Western: ECL Rabbit IgG HRP-Linked 1ml | 1:10000 | Th. Geyer, NA934  RRID:AB_772206 |
| Nucleus staining | IF: Hoechst | 1:5000 | Sigma, 33258 |

**Table S4: Primer list.**

| Gene | Forward 5´-> 3´ | Reverse 5´-> 3´ |
| --- | --- | --- |
| 18s | ACCCGTTGAACCCCATTCGTGA | GCCTCACTAAACCATCCAATCGG |
| GAPDH | AGAGGCAGGGATGATGTTCT | TCTGCTGATGCCCCCATGTT |
| RBM20 – exon9 | GAGTGTACACAGTTACATGCAC | GTG GGACCTCGGGGAGA |
| RBM20 | CCTCCACTTGCCGCATATCTGT | AGACCAGGCATTTCTGAGCGTG |
| MYL2 | CGGAGAGGTTTTCCAAGGAGGA | CTCTTCTCCGTGGGTGATGATG |
| NR2F | CCGACCGGGTGGTCGCCTTTATGGA | CGGCTGGTTGGGGTACTGGCTCCTA |
| TTN-N2B | CCAATGAGTATGGCAGTGTCA | TACGTTCCGGAAGTAATTTGC |
| TTN-N2BA | GCCACACTAACTGTGACAGAGG | GGCTGCCTTACCCACAAAAG |
| RYR2-24bp | GTCACAGGATCCCAACGCAG | CTTTGCTGGCACTGATTGTCTG |
| RYR2 | CTTGAGGTTGGCTTTCTGCCAG | TGTGCCAGCAAAGAGAGGAGAC |
| LDB3-exon5 | TCAAAGCGTCCCATTCCCATC | CGGGAGAA GCAGGGCTAAA |
| LDB3 | ACCTCGTGGTGGCCATTG | GTGGAGATGGGAATGGGACG |
| CAMk2D-exon14 | CCATCTTGACAACTATGCTGGCT | GAACACTCGAACTGGACTTCCT |
| CAMk2D | ACACGGTGACTCCTGAAGCCAA | GTCTCCTGTCTGTGCATCATGG |
| TRDN-exon9 | GTCCATGGGGATTTAAAACCAGG | CTTCAAGGGCAGGTGATGC |
| TRDN | GGAGGACAAAGAGAAAGCAGCTG | AGGTGGAATGGCTGGGCTTTGT |
| IMMT-exon5-6 | AAACAGCCTGCCTCACAACT | TCCTTCAATGCACCCTCCAC |
| IMMT | CAGGCTGTCAATGCACACTCCA | CATCTACTGCCTTTCTGCGTTCC |

**Table S5: Top 100 differentially expressed genes resLVNC vs LVNC.**

| ENTREZID | SYMBOL | GENENAME | Log2 Fold Change | Raw P-value | Adjusted P-value |
| --- | --- | --- | --- | --- | --- |
| 358 | AQP1 | aquaporin 1 (Colton blood group) | -1.951±0.238 | 6.32 × 10^-16 | 1.30 × 10^-11 |
| 3959 | LGALS3BP | galectin 3 binding protein | -2.607±0.338 | 4.99 × 10^-14 | 3.52 × 10^-10 |
| 102724652 | LOC102724652 | crystallin alpha A2 | -0.756±0.804 | 6.79 × 10^-14 | 3.52 × 10^-10 |
| 1409 | CRYAA | crystallin alpha A | -0.754±0.804 | 6.83 × 10^-14 | 3.52 × 10^-10 |
| 112 | ADCY6 | adenylate cyclase 6 | -1.882±0.256 | 4.54 × 10^-13 | 1.87 × 10^-9 |
| 26001 | RNF167 | ring finger protein 167 | -1.402±0.193 | 6.01 × 10^-13 | 2.07 × 10^-9 |
| 80023 | NRSN2 | neurensin 2 | -2.705±0.373 | 1.09 × 10^-12 | 3.20 × 10^-9 |
| 3949 | LDLR | low density lipoprotein receptor | -2.188±0.308 | 2.37 × 10^-12 | 6.10 × 10^-9 |
| 37 | ACADVL | acyl-CoA dehydrogenase very long chain | -1.572±0.224 | 4.33 × 10^-12 | 9.93 × 10^-9 |
| 6840 | SVIL | supervillin | -0.945±0.137 | 6.11 × 10^-12 | 1.15 × 10^-8 |
| 8048 | CSRP3 | cysteine and glycine rich protein 3 | -1.743±0.250 | 6.04 × 10^-12 | 1.15 × 10^-8 |
| 6604 | SMARCD3 | SWI/SNF related, matrix associated, actin dependent regulator of chromatin, subfamily d, member 3 | -1.552±0.223 | 6.73 × 10^-12 | 1.16 × 10^-8 |
| 11180 | WDR6 | WD repeat domain 6 | -2.405±0.342 | 7.39 × 10^-12 | 1.17 × 10^-8 |
| 327 | APEH | acylaminoacyl-peptide hydrolase | -2.713±0.395 | 2.12 × 10^-11 | 3.12 × 10^-8 |
| 2044 | EPHA5 | EPH receptor A5 | 4.556±0.641 | 2.87 × 10^-11 | 3.94 × 10^-8 |
| 7284 | TUFM | Tu translation elongation factor, mitochondrial | -2.311±0.357 | 1.81 × 10^-10 | 2.12 × 10^-7 |
| 6711 | SPTBN1 | spectrin beta, non-erythrocytic 1 | -1.223±0.189 | 1.85 × 10^-10 | 2.12 × 10^-7 |
| 10221 | TRIB1 | tribbles pseudokinase 1 | -1.661±0.257 | 1.71 × 10^-10 | 2.12 × 10^-7 |
| 1523 | CUX1 | cut like homeobox 1 | -1.108±0.173 | 2.05 × 10^-10 | 2.22 × 10^-7 |
| 4240 | MFGE8 | milk fat globule EGF and factor V/VIII domain containing | -2.654±0.407 | 2.56 × 10^-10 | 2.64 × 10^-7 |
| 7057 | THBS1 | thrombospondin 1 | -1.927±0.302 | 3.81 × 10^-10 | 3.74 × 10^-7 |
| 286336 | FAM78A | family with sequence similarity 78 member A | -1.999±0.314 | 4.17 × 10^-10 | 3.91 × 10^-7 |
| 25805 | BAMBI | BMP and activin membrane bound inhibitor | -1.781±0.282 | 5.41 × 10^-10 | 4.85 × 10^-7 |
| 163702 | IFNLR1 | interferon lambda receptor 1 | -2.356±0.375 | 6.12 × 10^-10 | 5.26 × 10^-7 |
| 257194 | NEGR1 | neuronal growth regulator 1 | 2.033±0.326 | 7.14 × 10^-10 | 5.89 × 10^-7 |
| 3693 | ITGB5 | integrin subunit beta 5 | -1.973±0.318 | 9.20 × 10^-10 | 7.30 × 10^-7 |
| 30845 | EHD3 | EH domain containing 3 | -1.924±0.310 | 1.16 × 10^-9 | 8.86 × 10^-7 |
| 196385 | DNAH10 | dynein axonemal heavy chain 10 | 2.718±0.433 | 1.60 × 10^-9 | 1.14 × 10^-6 |
| 593 | BCKDHA | branched chain keto acid dehydrogenase E1 subunit alpha | -2.845±0.453 | 1.60 × 10^-9 | 1.14 × 10^-6 |
| 826 | CAPNS1 | calpain small subunit 1 | -1.693±0.275 | 1.66 × 10^-9 | 1.14 × 10^-6 |
| 146691 | TOM1L2 | target of myb1 like 2 membrane trafficking protein | -1.667±0.273 | 1.79 × 10^-9 | 1.15 × 10^-6 |
| 58498 | MYL7 | myosin light chain 7 | -2.473±0.395 | 1.77 × 10^-9 | 1.15 × 10^-6 |
| 27122 | DKK3 | dickkopf WNT signaling pathway inhibitor 3 | -1.347±0.223 | 2.83 × 10^-9 | 1.77 × 10^-6 |
| 6506 | SLC1A2 | solute carrier family 1 member 2 | 2.574±0.421 | 3.13 × 10^-9 | 1.85 × 10^-6 |
| 2896 | GRN | granulin precursor | -3.089±0.500 | 3.06 × 10^-9 | 1.85 × 10^-6 |
| 3679 | ITGA7 | integrin subunit alpha 7 | -2.036±0.338 | 3.27 × 10^-9 | 1.85 × 10^-6 |
| 65997 | RASL11B | RAS like family 11 member B | -1.859±0.310 | 3.32 × 10^-9 | 1.85 × 10^-6 |
| 55089 | SLC38A4 | solute carrier family 38 member 4 | 2.996±0.496 | 3.42 × 10^-9 | 1.86 × 10^-6 |
| 1917 | EEF1A2 | eukaryotic translation elongation factor 1 alpha 2 | -2.815±0.462 | 3.92 × 10^-9 | 2.07 × 10^-6 |
| 200162 | SPAG17 | sperm associated antigen 17 | 4.466±0.686 | 4.44 × 10^-9 | 2.26 × 10^-6 |
| 57658 | CALCOCO1 | calcium binding and coiled-coil domain 1 | -1.204±0.203 | 4.48 × 10^-9 | 2.26 × 10^-6 |
| 4607 | MYBPC3 | myosin binding protein C3 | -2.193±0.362 | 4.73 × 10^-9 | 2.32 × 10^-6 |
| 7436 | VLDLR | very low density lipoprotein receptor | -1.059±0.180 | 5.31 × 10^-9 | 2.55 × 10^-6 |
| 4192 | MDK | midkine | -1.866±0.311 | 5.51 × 10^-9 | 2.55 × 10^-6 |
| 57158 | JPH2 | junctophilin 2 | -1.832±0.308 | 5.56 × 10^-9 | 2.55 × 10^-6 |
| 3213 | HOXB3 | homeobox B3 | 1.997±0.340 | 8.83 × 10^-9 | 3.96 × 10^-6 |
| 1284 | COL4A2 | collagen type IV alpha 2 chain | -2.368±0.402 | 1.25 × 10^-8 | 5.47 × 10^-6 |
| 65018 | PINK1 | PTEN induced kinase 1 | -2.786±0.467 | 1.32 × 10^-8 | 5.67 × 10^-6 |
| 146862 | UNC45B | unc-45 myosin chaperone B | -1.374±0.238 | 1.37 × 10^-8 | 5.75 × 10^-6 |
| 2975 | GTF3C1 | general transcription factor IIIC subunit 1 | -1.249±0.219 | 1.67 × 10^-8 | 6.90 × 10^-6 |
| 94005 | PIGS | phosphatidylinositol glycan anchor biosynthesis class S | -1.589±0.281 | 2.15 × 10^-8 | 8.40 × 10^-6 |
| 4924 | NUCB1 | nucleobindin 1 | -2.381±0.411 | 2.16 × 10^-8 | 8.40 × 10^-6 |
| 7074 | TIAM1 | TIAM Rac1 associated GEF 1 | 2.195±0.382 | 2.10 × 10^-8 | 8.40 × 10^-6 |
| 30008 | EFEMP2 | EGF containing fibulin extracellular matrix protein 2 | -2.143±0.377 | 2.26 × 10^-8 | 8.65 × 10^-6 |
| 4258 | MGST2 | microsomal glutathione S-transferase 2 | -1.423±0.252 | 2.41 × 10^-8 | 9.05 × 10^-6 |
| 9844 | ELMO1 | engulfment and cell motility 1 | 2.221±0.387 | 2.66 × 10^-8 | 9.78 × 10^-6 |
| 777 | CACNA1E | calcium voltage-gated channel subunit alpha1 E | 3.955±0.659 | 2.86 × 10^-8 | 1.04 × 10^-5 |
| 147906 | DACT3 | dishevelled binding antagonist of beta catenin 3 | -1.751±0.313 | 3.14 × 10^-8 | 1.12 × 10^-5 |
| 23467 | NPTXR | neuronal pentraxin receptor | -1.689±0.300 | 3.25 × 10^-8 | 1.14 × 10^-5 |
| 8828 | NRP2 | neuropilin 2 | -1.377±0.247 | 3.37 × 10^-8 | 1.16 × 10^-5 |
| 9348 | NDST3 | N-deacetylase and N-sulfotransferase 3 | 3.872±0.646 | 3.45 × 10^-8 | 1.17 × 10^-5 |
| 3913 | LAMB2 | laminin subunit beta 2 | -2.071±0.363 | 3.66 × 10^-8 | 1.22 × 10^-5 |
| 115123 | MARCHF3 | membrane associated ring-CH-type finger 3 | -1.640±0.298 | 4.21 × 10^-8 | 1.38 × 10^-5 |
| 79915 | ATAD5 | ATPase family AAA domain containing 5 | 1.722±0.310 | 4.37 × 10^-8 | 1.41 × 10^-5 |
| 3673 | ITGA2 | integrin subunit alpha 2 | -1.838±0.338 | 4.48 × 10^-8 | 1.42 × 10^-5 |
| 8566 | PDXK | pyridoxal kinase | -1.529±0.276 | 4.65 × 10^-8 | 1.45 × 10^-5 |
| 5156 | PDGFRA | platelet derived growth factor receptor alpha | -1.898±0.345 | 4.81 × 10^-8 | 1.48 × 10^-5 |
| 5922 | RASA2 | RAS p21 protein activator 2 | 1.515±0.277 | 5.15 × 10^-8 | 1.56 × 10^-5 |
| 23038 | WDTC1 | WD and tetratricopeptide repeats 1 | -2.258±0.412 | 5.70 × 10^-8 | 1.70 × 10^-5 |
| 10723 | SLC12A7 | solute carrier family 12 member 7 | -3.345±0.569 | 6.20 × 10^-8 | 1.83 × 10^-5 |
| 51555 | PEX5L | peroxisomal biogenesis factor 5 like | 2.645±0.470 | 6.50 × 10^-8 | 1.89 × 10^-5 |
| 10622 | POLR3G | RNA polymerase III subunit G | 2.255±0.408 | 7.05 × 10^-8 | 2.02 × 10^-5 |
| 2621 | GAS6 | growth arrest specific 6 | -2.942±0.521 | 7.15 × 10^-8 | 2.02 × 10^-5 |
| 8626 | TP63 | tumor protein p63 | 3.631±0.619 | 7.61 × 10^-8 | 2.12 × 10^-5 |
| 219595 | FOLH1B | folate hydrolase 1B (pseudogene) | 3.548±0.623 | 7.96 × 10^-8 | 2.19 × 10^-5 |
| 2846 | LPAR4 | lysophosphatidic acid receptor 4 | 3.554±0.617 | 8.07 × 10^-8 | 2.19 × 10^-5 |
| 6900 | CNTN2 | contactin 2 | 4.292±0.734 | 8.81 × 10^-8 | 2.36 × 10^-5 |
| 672 | BRCA1 | BRCA1 DNA repair associated | 1.768±0.328 | 8.93 × 10^-8 | 2.36 × 10^-5 |
| 5339 | PLEC | plectin | -2.342±0.423 | 9.48 × 10^-8 | 2.48 × 10^-5 |
| 9469 | CHST3 | carbohydrate sulfotransferase 3 | -1.829±0.338 | 9.69 × 10^-8 | 2.50 × 10^-5 |
| 10690 | FUT9 | fucosyltransferase 9 | 3.495±0.619 | 1.03 × 10^-7 | 2.61 × 10^-5 |
| 29106 | SCG3 | secretogranin III | 3.862±0.668 | 1.09 × 10^-7 | 2.74 × 10^-5 |
| 25844 | YIPF3 | Yip1 domain family member 3 | -1.948±0.361 | 1.12 × 10^-7 | 2.79 × 10^-5 |
| 5819 | NECTIN2 | nectin cell adhesion molecule 2 | -2.739±0.501 | 1.17 × 10^-7 | 2.86 × 10^-5 |
| 389 | RHOC | ras homolog family member C | -2.059±0.378 | 1.20 × 10^-7 | 2.89 × 10^-5 |
| 5315 | PKM | pyruvate kinase M1/2 | -1.854±0.340 | 1.19 × 10^-7 | 2.89 × 10^-5 |
| 2137 | EXTL3 | exostosin like glycosyltransferase 3 | -1.296±0.243 | 1.33 × 10^-7 | 3.16 × 10^-5 |
| 7168 | TPM1 | tropomyosin 1 | -1.529±0.283 | 1.49 × 10^-7 | 3.49 × 10^-5 |
| 105375661 | NA | NA | 5.129±0.864 | 1.54 × 10^-7 | 3.58 × 10^-5 |
| 23385 | NCSTN | nicastrin | -1.005±0.190 | 1.60 × 10^-7 | 3.66 × 10^-5 |
| 84457 | PHYHIPL | phytanoyl-CoA 2-hydroxylase interacting protein like | 3.390±0.599 | 1.63 × 10^-7 | 3.69 × 10^-5 |
| 58476 | TP53INP2 | tumor protein p53 inducible nuclear protein 2 | -1.388±0.262 | 1.65 × 10^-7 | 3.71 × 10^-5 |
| 5454 | POU3F2 | POU class 3 homeobox 2 | 5.074±0.839 | 1.69 × 10^-7 | 3.75 × 10^-5 |
| 91304 | TMEM259 | transmembrane protein 259 | -2.016±0.375 | 1.73 × 10^-7 | 3.79 × 10^-5 |
| 56981 | PRDM11 | PR/SET domain 11 | -1.270±0.242 | 1.78 × 10^-7 | 3.87 × 10^-5 |
| 339123 | JMJD8 | jumonji domain containing 8 | -3.411±0.598 | 1.88 × 10^-7 | 4.03 × 10^-5 |
| 558 | AXL | AXL receptor tyrosine kinase | -1.991±0.379 | 1.91 × 10^-7 | 4.07 × 10^-5 |
| 7005 | TEAD3 | TEA domain transcription factor 3 | -2.006±0.377 | 2.07 × 10^-7 | 4.35 × 10^-5 |
| 3636 | INPPL1 | inositol polyphosphate phosphatase like 1 | -1.666±0.313 | 2.40 × 10^-7 | 5.00 × 10^-5 |
| 780 | DDR1 | discoidin domain receptor tyrosine kinase 1 | -2.286±0.426 | 2.52 × 10^-7 | 5.09 × 10^-5 |

**Table S6: Top 100 differentially expressed genes resDCM1 vs DCM1.**

| ENTREZID | SYMBOL | GENENAME | Log2 Fold Change | Raw P-value | Adjusted P-value |
| --- | --- | --- | --- | --- | --- |
| 10399 | RACK1 | receptor for activated C kinase 1 | -2.185±0.116 | 4.01 × 10^-79 | 7.99 × 10^-75 |
| 6134 | RPL10 | ribosomal protein L10 | -2.533±0.139 | 4.09 × 10^-74 | 4.07 × 10^-70 |
| 1937 | EEF1G | eukaryotic translation elongation factor 1 gamma | -2.088±0.119 | 1.82 × 10^-68 | 1.21 × 10^-64 |
| 7316 | UBC | ubiquitin C | -2.474±0.148 | 2.34 × 10^-62 | 1.16 × 10^-58 |
| 230 | ALDOC | aldolase, fructose-bisphosphate C | -3.683±0.237 | 1.21 × 10^-53 | 4.81 × 10^-50 |
| 728658 | RPL13AP5 | ribosomal protein L13a pseudogene 5 | -2.172±0.146 | 9.01 × 10^-50 | 2.99 × 10^-46 |
| 967 | CD63 | CD63 molecule | -2.905±0.209 | 5.61 × 10^-43 | 1.60 × 10^-39 |
| 6202 | RPS8 | ribosomal protein S8 | -1.717±0.125 | 2.06 × 10^-42 | 5.13 × 10^-39 |
| 6143 | RPL19 | ribosomal protein L19 | -1.668±0.129 | 2.88 × 10^-38 | 6.38 × 10^-35 |
| 4637 | MYL6 | myosin light chain 6 | -1.778±0.141 | 4.23 × 10^-36 | 8.43 × 10^-33 |
| 6208 | RPS14 | ribosomal protein S14 | -2.504±0.197 | 5.80 × 10^-36 | 1.05 × 10^-32 |
| 498 | ATP5F1A | ATP synthase F1 subunit alpha | -1.588±0.128 | 1.62 × 10^-35 | 2.68 × 10^-32 |
| 3959 | LGALS3BP | galectin 3 binding protein | -3.136±0.251 | 5.36 × 10^-35 | 8.21 × 10^-32 |
| 1642 | DDB1 | damage specific DNA binding protein 1 | -1.419±0.115 | 1.11 × 10^-34 | 1.58 × 10^-31 |
| 5213 | PFKM | phosphofructokinase, muscle | -2.462±0.200 | 1.52 × 10^-34 | 2.01 × 10^-31 |
| 10939 | AFG3L2 | AFG3 like matrix AAA peptidase subunit 2 | -1.598±0.132 | 1.05 × 10^-33 | 1.31 × 10^-30 |
| 800 | CALD1 | caldesmon 1 | 1.738±0.145 | 3.72 × 10^-33 | 4.35 × 10^-30 |
| 9669 | EIF5B | eukaryotic translation initiation factor 5B | 2.253±0.188 | 4.53 × 10^-33 | 5.01 × 10^-30 |
| 4720 | NDUFS2 | NADH:ubiquinone oxidoreductase core subunit S2 | -1.894±0.159 | 1.54 × 10^-32 | 1.62 × 10^-29 |
| 3945 | LDHB | lactate dehydrogenase B | -1.929±0.164 | 6.91 × 10^-32 | 6.88 × 10^-29 |
| 6130 | RPL7A | ribosomal protein L7a | -2.125±0.181 | 2.28 × 10^-31 | 2.16 × 10^-28 |
| 4541 | ND6 | NADH dehydrogenase subunit 6 | 3.950±0.337 | 2.64 × 10^-31 | 2.39 × 10^-28 |
| 5250 | SLC25A3 | solute carrier family 25 member 3 | -1.654±0.145 | 4.05 × 10^-30 | 3.51 × 10^-27 |
| 7415 | VCP | valosin containing protein | -1.926±0.171 | 2.06 × 10^-29 | 1.71 × 10^-26 |
| 6132 | RPL8 | ribosomal protein L8 | -2.745±0.243 | 6.39 × 10^-29 | 5.09 × 10^-26 |
| 6415 | SELENOW | selenoprotein W | -3.895±0.343 | 8.37 × 10^-29 | 6.41 × 10^-26 |
| 6122 | RPL3 | ribosomal protein L3 | -1.926±0.173 | 1.10 × 10^-28 | 8.10 × 10^-26 |
| 374393 | FAM111B | FAM111 trypsin like peptidase B | 2.504±0.226 | 1.42 × 10^-28 | 1.01 × 10^-25 |
| 388524 | RPSA2 | ribosomal protein SA 2 | -2.141±0.194 | 4.01 × 10^-28 | 2.75 × 10^-25 |
| 63967 | CLSPN | claspin | 1.961±0.179 | 7.10 × 10^-28 | 4.56 × 10^-25 |
| 4678 | NASP | nuclear autoantigenic sperm protein | 1.249±0.114 | 7.03 × 10^-28 | 4.56 × 10^-25 |
| 9500 | MAGED1 | MAGE family member D1 | -2.188±0.201 | 1.90 × 10^-27 | 1.18 × 10^-24 |
| 1499 | CTNNB1 | catenin beta 1 | -1.549±0.143 | 3.73 × 10^-27 | 2.25 × 10^-24 |
| 6175 | RPLP0 | ribosomal protein lateral stalk subunit P0 | -1.525±0.142 | 1.17 × 10^-26 | 6.83 × 10^-24 |
| 2170 | FABP3 | fatty acid binding protein 3 | -1.571±0.148 | 2.26 × 10^-26 | 1.29 × 10^-23 |
| 653513 | LOC653513 | phosphodiesterase 4D interacting protein-like | -2.169±0.204 | 3.28 × 10^-26 | 1.82 × 10^-23 |
| 23326 | USP22 | ubiquitin specific peptidase 22 | -1.220±0.117 | 2.20 × 10^-25 | 1.19 × 10^-22 |
| 3921 | RPSA | ribosomal protein SA | -2.601±0.247 | 3.17 × 10^-25 | 1.66 × 10^-22 |
| 1340 | COX6B1 | cytochrome c oxidase subunit 6B1 | -1.512±0.146 | 3.53 × 10^-25 | 1.80 × 10^-22 |
| 506 | ATP5F1B | ATP synthase F1 subunit beta | -1.563±0.152 | 1.11 × 10^-24 | 5.55 × 10^-22 |
| 84081 | NSRP1 | nuclear speckle splicing regulatory protein 1 | 1.765±0.172 | 1.27 × 10^-24 | 6.15 × 10^-22 |
| 1345 | COX6C | cytochrome c oxidase subunit 6C | -1.428±0.139 | 1.60 × 10^-24 | 7.60 × 10^-22 |
| 5660 | PSAP | prosaposin | -2.357±0.230 | 1.99 × 10^-24 | 9.23 × 10^-22 |
| 25824 | PRDX5 | peroxiredoxin 5 | -3.232±0.308 | 3.36 × 10^-24 | 1.52 × 10^-21 |
| 118 | ADD1 | adducin 1 | -1.488±0.147 | 4.45 × 10^-24 | 1.97 × 10^-21 |
| 10574 | CCT7 | chaperonin containing TCP1 subunit 7 | -1.571±0.155 | 6.99 × 10^-24 | 3.03 × 10^-21 |
| 517 | ATP5MC2 | ATP synthase membrane subunit c locus 2 | -2.211±0.219 | 9.40 × 10^-24 | 3.99 × 10^-21 |
| 1666 | DECR1 | 2,4-dienoyl-CoA reductase 1 | -1.791±0.178 | 1.06 × 10^-23 | 4.39 × 10^-21 |
| 5858 | PZP | PZP alpha-2-macroglobulin like | 2.820±0.281 | 1.71 × 10^-23 | 6.93 × 10^-21 |
| 23107 | MRPS27 | mitochondrial ribosomal protein S27 | -2.372±0.236 | 2.62 × 10^-23 | 1.05 × 10^-20 |
| 3032 | HADHB | hydroxyacyl-CoA dehydrogenase trifunctional multienzyme complex subunit beta | -1.385±0.140 | 6.07 × 10^-23 | 2.37 × 10^-20 |
| 309 | ANXA6 | annexin A6 | -1.379±0.140 | 8.71 × 10^-23 | 3.34 × 10^-20 |
| 3006 | H1-2 | H1.2 linker histone, cluster member | -2.927±0.292 | 8.89 × 10^-23 | 3.34 × 10^-20 |
| 4538 | ND4 | NADH dehydrogenase subunit 4 | 2.711±0.275 | 9.77 × 10^-23 | 3.61 × 10^-20 |
| 10541 | ANP32B | acidic nuclear phosphoprotein 32 family member B | 2.222±0.227 | 1.09 × 10^-22 | 3.95 × 10^-20 |
| 5430 | POLR2A | RNA polymerase II subunit A | 1.908±0.195 | 1.12 × 10^-22 | 3.99 × 10^-20 |
| 23521 | RPL13A | ribosomal protein L13a | -1.510±0.153 | 1.20 × 10^-22 | 4.19 × 10^-20 |
| 150082 | LCA5L | lebercilin LCA5 like | 3.067±0.311 | 1.30 × 10^-22 | 4.46 × 10^-20 |
| 284459 | ZNF875 | zinc finger protein 875 | -2.032±0.206 | 1.43 × 10^-22 | 4.82 × 10^-20 |
| 10106 | CTDSP2 | CTD small phosphatase 2 | -1.443±0.148 | 1.80 × 10^-22 | 5.96 × 10^-20 |
| 195828 | ZNF367 | zinc finger protein 367 | 1.524±0.157 | 2.30 × 10^-22 | 7.51 × 10^-20 |
| 37 | ACADVL | acyl-CoA dehydrogenase very long chain | -1.642±0.169 | 2.55 × 10^-22 | 8.21 × 10^-20 |
| 6136 | RPL12 | ribosomal protein L12 | -1.723±0.177 | 2.75 × 10^-22 | 8.68 × 10^-20 |
| 29896 | TRA2A | transformer 2 alpha homolog | 1.381±0.143 | 3.66 × 10^-22 | 1.14 × 10^-19 |
| 2137 | EXTL3 | exostosin like glycosyltransferase 3 | -2.093±0.216 | 4.63 × 10^-22 | 1.42 × 10^-19 |
| 8367 | H4C5 | H4 clustered histone 5 | -2.407±0.246 | 4.89 × 10^-22 | 1.48 × 10^-19 |
| 389247 | ING2-DT | ING2 divergent transcript | 4.380±0.451 | 1.07 × 10^-21 | 3.17 × 10^-19 |
| 2030 | SLC29A1 | solute carrier family 29 member 1 (Augustine blood group) | -2.442±0.253 | 1.28 × 10^-21 | 3.75 × 10^-19 |
| 4190 | MDH1 | malate dehydrogenase 1 | -1.312±0.137 | 1.44 × 10^-21 | 4.16 × 10^-19 |
| 6774 | STAT3 | signal transducer and activator of transcription 3 | -1.334±0.140 | 1.71 × 10^-21 | 4.87 × 10^-19 |
| 7314 | UBB | ubiquitin B | -1.216±0.128 | 1.87 × 10^-21 | 5.24 × 10^-19 |
| 5702 | PSMC3 | proteasome 26S subunit, ATPase 3 | -2.243±0.234 | 2.05 × 10^-21 | 5.68 × 10^-19 |
| 54892 | NCAPG2 | non-SMC condensin II complex subunit G2 | 1.680±0.176 | 2.13 × 10^-21 | 5.81 × 10^-19 |
| 10476 | ATP5PD | ATP synthase peripheral stalk subunit d | -2.236±0.235 | 4.50 × 10^-21 | 1.21 × 10^-18 |
| 55215 | FANCI | FA complementation group I | 1.595±0.169 | 5.33 × 10^-21 | 1.42 × 10^-18 |
| 1072 | CFL1 | cofilin 1 | -1.465±0.156 | 9.57 × 10^-21 | 2.51 × 10^-18 |
| 3073 | HEXA | hexosaminidase subunit alpha | -2.262±0.238 | 1.60 × 10^-20 | 4.15 × 10^-18 |
| 5518 | PPP2R1A | protein phosphatase 2 scaffold subunit Aalpha | -2.462±0.265 | 3.05 × 10^-20 | 7.78 × 10^-18 |
| 284361 | EMC10 | ER membrane protein complex subunit 10 | 2.196±0.239 | 3.19 × 10^-20 | 8.04 × 10^-18 |
| 161497 | STRC | stereocilin | 3.670±0.394 | 4.00 × 10^-20 | 9.83 × 10^-18 |
| 1327 | COX4I1 | cytochrome c oxidase subunit 4I1 | -2.252±0.244 | 3.95 × 10^-20 | 9.83 × 10^-18 |
| 105378510 | NA | NA | 3.944±0.427 | 5.93 × 10^-20 | 1.42 × 10^-17 |
| 9168 | TMSB10 | thymosin beta 10 | -1.659±0.181 | 5.97 × 10^-20 | 1.42 × 10^-17 |
| 27000 | DNAJC2 | DnaJ heat shock protein family (Hsp40) member C2 | 1.434±0.157 | 5.90 × 10^-20 | 1.42 × 10^-17 |
| 4509 | ATP8 | ATP synthase F0 subunit 8 | 2.997±0.329 | 1.22 × 10^-19 | 2.85 × 10^-17 |
| 8031 | NCOA4 | nuclear receptor coactivator 4 | -1.605±0.177 | 1.59 × 10^-19 | 3.68 × 10^-17 |
| 4539 | ND4L | NADH dehydrogenase subunit 4L | 2.440±0.269 | 1.67 × 10^-19 | 3.83 × 10^-17 |
| 6567 | SLC16A2 | solute carrier family 16 member 2 | -2.574±0.283 | 2.21 × 10^-19 | 5.00 × 10^-17 |
| 10916 | MAGED2 | MAGE family member D2 | -1.939±0.215 | 2.78 × 10^-19 | 6.22 × 10^-17 |
| 220869 | ZNG1E | Zn regulated GTPase metalloprotein activator 1E | 1.305±0.146 | 3.15 × 10^-19 | 6.97 × 10^-17 |
| 6155 | RPL27 | ribosomal protein L27 | -1.251±0.140 | 4.26 × 10^-19 | 9.33 × 10^-17 |
| 4540 | ND5 | NADH dehydrogenase subunit 5 | 2.769±0.308 | 4.59 × 10^-19 | 9.94 × 10^-17 |
| 2934 | GSN | gelsolin | -1.993±0.223 | 5.23 × 10^-19 | 1.12 × 10^-16 |
| 343099 | CCDC18 | coiled-coil domain containing 18 | 1.693±0.190 | 6.03 × 10^-19 | 1.28 × 10^-16 |
| 6141 | RPL18 | ribosomal protein L18 | -2.350±0.260 | 6.74 × 10^-19 | 1.41 × 10^-16 |
| 8508 | NIPSNAP1 | nipsnap homolog 1 | -2.286±0.254 | 6.85 × 10^-19 | 1.42 × 10^-16 |
| 6185 | RPN2 | ribophorin II | -1.588±0.178 | 6.96 × 10^-19 | 1.43 × 10^-16 |
| 284695 | ZNF326 | zinc finger protein 326 | 1.614±0.182 | 8.01 × 10^-19 | 1.63 × 10^-16 |
| 1938 | EEF2 | eukaryotic translation elongation factor 2 | -0.928±0.105 | 9.17 × 10^-19 | 1.85 × 10^-16 |
| 8899 | PRPF4B | pre-mRNA processing factor 4B | 1.401±0.159 | 9.67 × 10^-19 | 1.93 × 10^-16 |

**Table S7: Top 24 differentially expressed genes LVNC vs DCM1.**

| ENTREZID | SYMBOL | GENENAME | Log2 Fold Change | Raw P-value | Adjusted P-value |
| --- | --- | --- | --- | --- | --- |
| 8287 | USP9Y | ubiquitin specific peptidase 9 Y-linked | -7.291±0.597 | 1.28 × 10^-21 | 2.44 × 10^-17 |
| 8653 | DDX3Y | DEAD-box helicase 3 Y-linked | -7.411±0.607 | 2.42 × 10^-20 | 2.30 × 10^-16 |
| 7404 | UTY | ubiquitously transcribed tetratricopeptide repeat containing, Y-linked | -6.797±0.650 | 1.94 × 10^-16 | 1.23 × 10^-12 |
| 6192 | RPS4Y1 | ribosomal protein S4 Y-linked 1 | -6.183±0.666 | 2.64 × 10^-14 | 1.26 × 10^-10 |
| 8284 | KDM5D | lysine demethylase 5D | -6.096±0.671 | 4.93 × 10^-14 | 1.87 × 10^-10 |
| 9086 | EIF1AY | eukaryotic translation initiation factor 1A Y-linked | -6.069±0.676 | 7.59 × 10^-14 | 2.41 × 10^-10 |
| 7544 | ZFY | zinc finger protein Y-linked | -5.719±0.685 | 8.24 × 10^-13 | 2.24 × 10^-9 |
| 84171 | LOXL4 | lysyl oxidase like 4 | -3.285±0.494 | 3.87 × 10^-11 | 9.21 × 10^-8 |
| 246126 | TXLNGY | taxilin gamma Y-linked (pseudogene) | -5.205±0.704 | 5.54 × 10^-11 | 1.17 × 10^-7 |
| 64595 | TTTY15 | testis expressed transcript, Y-linked 15 | -4.786±0.723 | 8.51 × 10^-10 | 1.62 × 10^-6 |
| 22829 | NLGN4Y | neuroligin 4 Y-linked | -3.967±0.672 | 1.08 × 10^-8 | 1.86 × 10^-5 |
| 27063 | ANKRD1 | ankyrin repeat domain 1 | -2.349±0.445 | 1.52 × 10^-7 | 2.41 × 10^-4 |
| 4633 | MYL2 | myosin light chain 2 | -2.291±0.437 | 1.82 × 10^-7 | 2.67 × 10^-4 |
| 347273 | CAVIN4 | caveolae associated protein 4 | -2.168±0.416 | 2.07 × 10^-7 | 2.82 × 10^-4 |
| 378951 | RBMY1J | RNA binding motif protein Y-linked family 1 member J | -3.824±0.754 | 2.70 × 10^-7 | 3.42 × 10^-4 |
| 1641 | DCX | doublecortin | 3.018±0.662 | 2.24 × 10^-6 | 2.66 × 10^-3 |
| 5940 | RBMY1A1 | RNA binding motif protein Y-linked family 1 member A1 | -3.279±0.765 | 3.71 × 10^-6 | 4.15 × 10^-3 |
| 1264 | CNN1 | calponin 1 | -2.009±0.449 | 8.88 × 10^-6 | 9.39 × 10^-3 |
| 1674 | DES | desmin | -2.452±0.575 | 1.73 × 10^-5 | 1.62 × 10^-2 |
| 728403 | TSPY8 | testis specific protein Y-linked 8 | -3.043±0.768 | 1.71 × 10^-5 | 1.62 × 10^-2 |
| 378949 | RBMY1D | RNA binding motif protein Y-linked family 1 member D | -3.011±0.768 | 1.79 × 10^-5 | 1.62 × 10^-2 |
| 392197 | USP17L7 | ubiquitin specific peptidase 17 like family member 7 | -2.554±0.764 | 5.49 × 10^-5 | 4.75 × 10^-2 |
| 57502 | NLGN4X | neuroligin 4 X-linked | 2.761±0.719 | 6.12 × 10^-5 | 4.92 × 10^-2 |
| 100289087 | TSPY10 | testis specific protein Y-linked 10 | -2.649±0.769 | 6.20 × 10^-5 | 4.92 × 10^-2 |

**Table S8: KEGG GOterm hits for Top100 differentially expressed genes LVNC vs resLVNC**.

| **ID** | **Term** | **Ontology Source** | **% Associate No. Genes** | **No. Of Genes** | **Associated Genes Found** |
| --- | --- | --- | --- | --- | --- |
| KEGG:04512 | ECM-receptor interaction | KEGG_13.05.2021 | 6.82 | 6 | COL4A2, ITGA2, ITGA7, ITGB5, LAMB2, THBS1 |
| KEGG:05410 | Hypertrophic cardiomyopathy | KEGG_13.05.2021 | 5.56 | 5 | ITGA2, ITGA7, ITGB5, MYBPC3, TPM1 |
| KEGG:05414 | Dilated cardiomyopathy | KEGG_13.05.2021 | 6.25 | 6 | ADCY6, ITGA2, ITGA7, ITGB5, MYBPC3, TPM1 |

**Table S9: KEGG GOterm hits for Top100 differentially expressed genes DCM1 vs resDCM1**.

| **ID** | **Term** | **Ontology Source** | **% Associate No. Genes** | **No. Of Genes** | **Associated Genes Found** |
| --- | --- | --- | --- | --- | --- |
| KEGG:00010 | Glycolysis/ Gluconeogenesis | KEGG_13.05.2021 | 4.48 | 3 | ALDOC, LDHB, PFKM |
| KEGG:03010 | Ribosome | KEGG_13.05.2021 | 8.23 | 13 | RPL10, RPL12, RPL13A, RPL18, RPL19, RPL27, RPL3, RPL7A, RPL8, RPLP0, RPS14, RPS8, RPSA |
| KEGG:05171 | Coronavirus | KEGG_13.05.2021 | 6.03 | 14 | RPL10, RPL12, RPL13A, RPL18, RPL19, RPL27, RPL3, RPL7A, RPL8, RPLP0, RPS14, RPS8, RPSA, STAT3 |
| KEGG:00190 | Oxidative Phosphorylation | KEGG_13.05.2021 | 9.77 | 13 | ATP5F1A, ATP5F1B, ATP5MC2, ATP5PD, ATP8, COX4I1, COX6B1, COX6C, ND4, ND4L, ND5, ND6, NDUFS2 |
| KEGG:04714 | Thermogenesis | KEGG_13.05.2021 | 5.60 | 13 | ATP5F1A, ATP5F1B, ATP5MC2, ATP5PD, ATP8, COX4I1, COX6B1, COX6C, ND4, ND4L, ND5, ND6, NDUFS2 |
| KEGG:05010 | Alzheimer | KEGG_13.05.2021 | 4.07 | 15 | ATP5F1A, ATP5F1B, ATP5MC2, ATP5PD, ATP8, COX4I1, COX6B1, COX6C, CTNNB1, ND4, ND4L, ND5, ND6, NDUFS2, PSMC3 |
| KEGG:05012 | Parkinson | KEGG_13.05.2021 | 6.43 | 16 | ATP5F1A, ATP5F1B, ATP5MC2, ATP5PD, ATP8, COX4I1, COX6B1, COX6C, ND4, ND4L, ND5, ND6, NDUFS2, PSMC3, UBB, UBC |
| KEGG:05014 | Amyotrophic lateral sclerosis | KEGG_13.05.2021 | 4.12 | 15 | ATP5F1A, ATP5F1B, ATP5MC2, ATP5PD, ATP8, COX4I1, COX6B1, COX6C, ND4, ND4L, ND5, ND6, NDUFS2, PSMC3, VCP |
| KEGG:05016 | Huntington | KEGG_13.05.2021 | 4.9 | 15 | ATP5F1A, ATP5F1B, ATP5MC2, ATP5PD, ATP8, COX4I1, COX6B1, COX6C, ND4, ND4L, ND5, ND6, NDUFS2, POLR2A, PSMC3 |
| KEGG:05020 | Prion disease | KEGG_13.05.2021 | 5.13 | 14 | ATP5F1A, ATP5F1B, ATP5MC2, ATP5PD, ATP8, COX4I1, COX6B1, COX6C, ND4, ND4L, ND5, ND6, NDUFS2, PSMC3 |
| KEGG:05415 | Diabetic cardiomyopathy | KEGG_13.05.2021 | 6.4 | 13 | ATP5F1A, ATP5F1B, ATP5MC2, ATP5PD, ATP8, COX4I1, COX6B1, COX6C, ND4, ND4L, ND5, ND6, NDUFS2 |

**References**

1. Chen, S., Zhou, Y., Chen, Y. & Gu, J. fastp: an ultra-fast all-in-one FASTQ preprocessor. *Bioinformatics* **34**, i884-i890 (2018).

2. Chen, S. Ultrafast one-pass FASTQ data preprocessing, quality control, and deduplication using fastp. *Imeta* **2**, e107 (2023).

3. Liao, Y., Smyth, G.K. & Shi, W. The Subread aligner: fast, accurate and scalable read mapping by seed-and-vote. *Nucleic Acids Res* **41**, e108 (2013).

4. Liao, Y., Smyth, G.K. & Shi, W. featureCounts: an efficient general purpose program for assigning sequence reads to genomic features. *Bioinformatics* **30**, 923-930 (2014).

5. Love, M.I., Huber, W. & Anders, S. Moderated estimation of fold change and dispersion for RNA-seq data with DESeq2. *Genome Biol* **15**, 550 (2014).

6. Anders, S., Reyes, A. & Huber, W. Detecting differential usage of exons from RNA-seq data. *Genome Res* **22**, 2008-2017 (2012).

7. Wolter, S.*, et al.* rapidSTORM: accurate, fast open-source software for localization microscopy. *Nat Methods* **9**, 1040-1041 (2012).

8. Doose, S. LOCAN: a python library for analyzing single-molecule localization microscopy data. *Bioinformatics* **38**, 2670-2672 (2022).

9. Schubert, E., Sander, J., Ester, M., Kriegel, H.P. & Xu, X. DBSCAN Revisited, Revisited. *ACM Transactions on Database Systems* **42**, 1-21 (2017).

10. Ebert, V.*, et al.* Convex hull as diagnostic tool in single-molecule localization microscopy. *Bioinformatics* **38**, 5421-5429 (2022).

11. Haupt, L.P.*, et al.* Doxorubicin induces cardiotoxicity in a pluripotent stem cell model of aggressive B cell lymphoma cancer patients. *Basic Res Cardiol* **117**, 13 (2022).

12. Nagai, T., Sawano, A., Park, E.S. & Miyawaki, A. Circularly permuted green fluorescent proteins engineered to sense Ca2+. *Proc Natl Acad Sci U S A* **98**, 3197-3202 (2001).
