# Supplementary material for: RBM20 variants disrupt Ca2+ handling and metabolism in dilated and non-compaction cardiomyopathy stem cell models
Source: Signal Transduct Target Ther. 2026 Jul 14;11:276. doi: 10.1038/s41392-026-02838-7 (PMC13365226; doi:10.1038/s41392-026-02838-7)
Supplement: Supplementary file 2 — Original Western Blot membranes [file 41392_2026_2838_MOESM2_ESM.pdf]

## Original Western blot membranes; Rebs et al., 2026

### Fig. 1 b/c

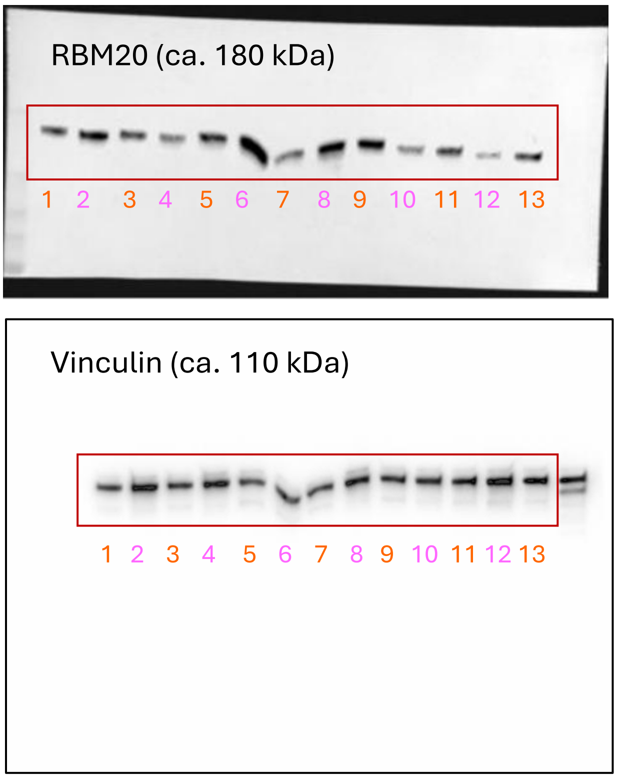


1 3 5 7 9 11 13 = resLVNC
2 4 6 8 10 12 = LVNC

### Fig. 1 b/c

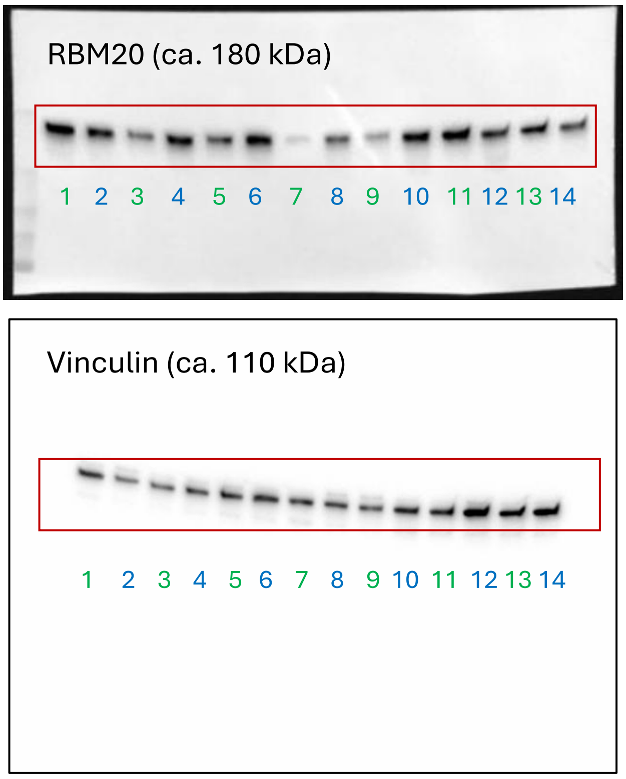


1 3 5 7 9 11 13 = resDCM
2 4 6 8 10 12 14 = DCM1

Used in analysis

# Original Western blot membranes; Rebs et al., 2026

**Fig. 6 d**

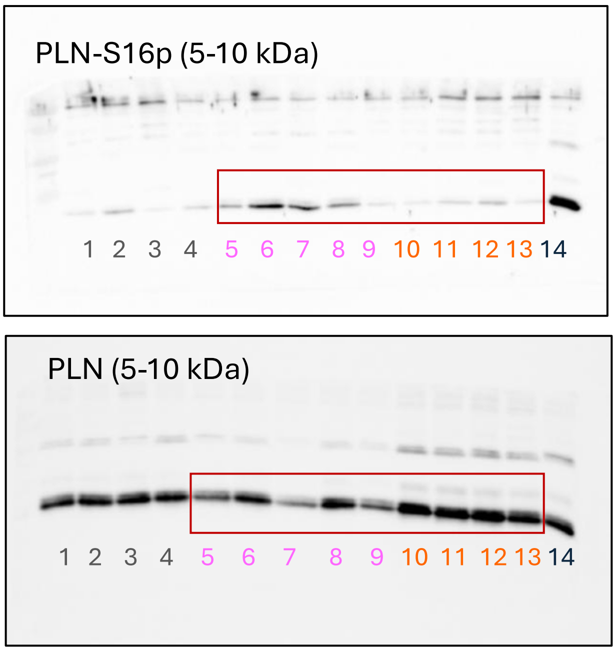


**Fig. 6 e**

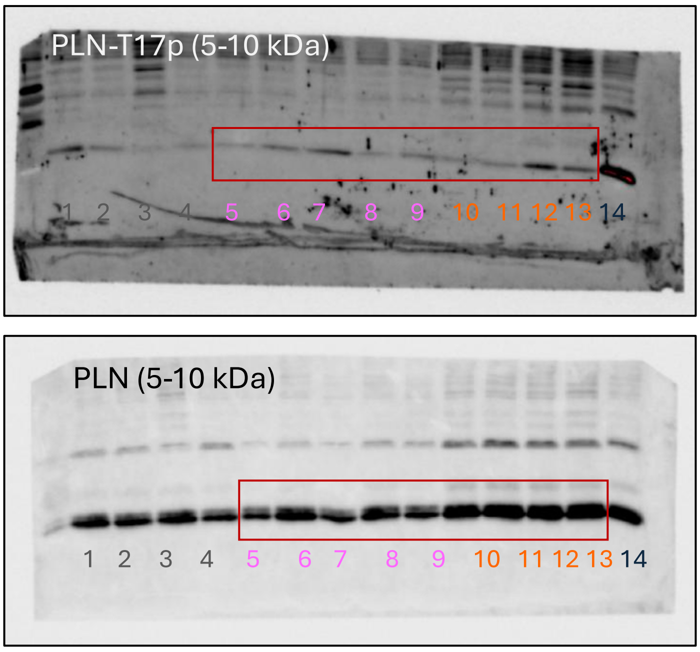


1 2 3 4 = other samples  5 6 7 8 9 = LVNC  10 11 12 13 = resLVNC  14 = Isoprenaline treated

**Fig. 6 d/f**

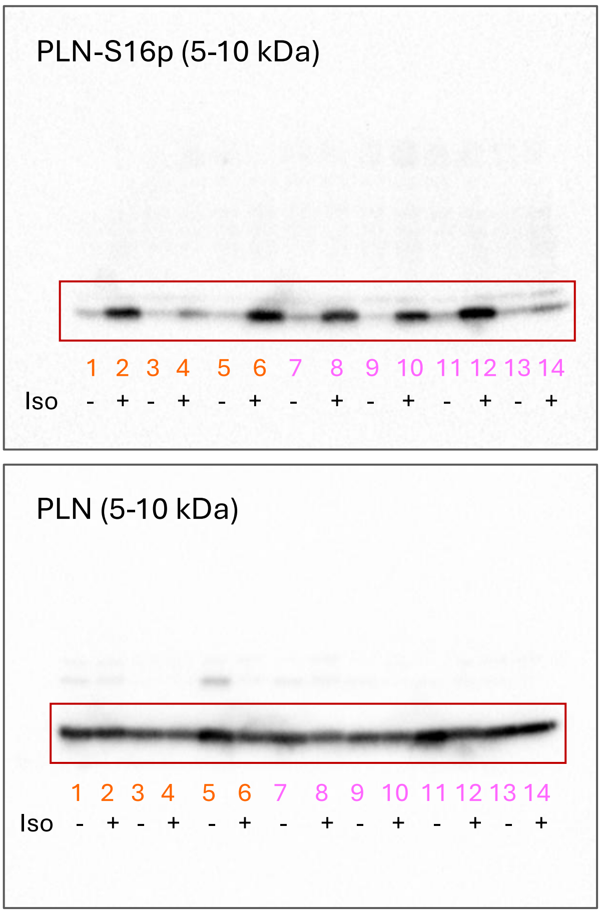


**Fig. 6 e/g**

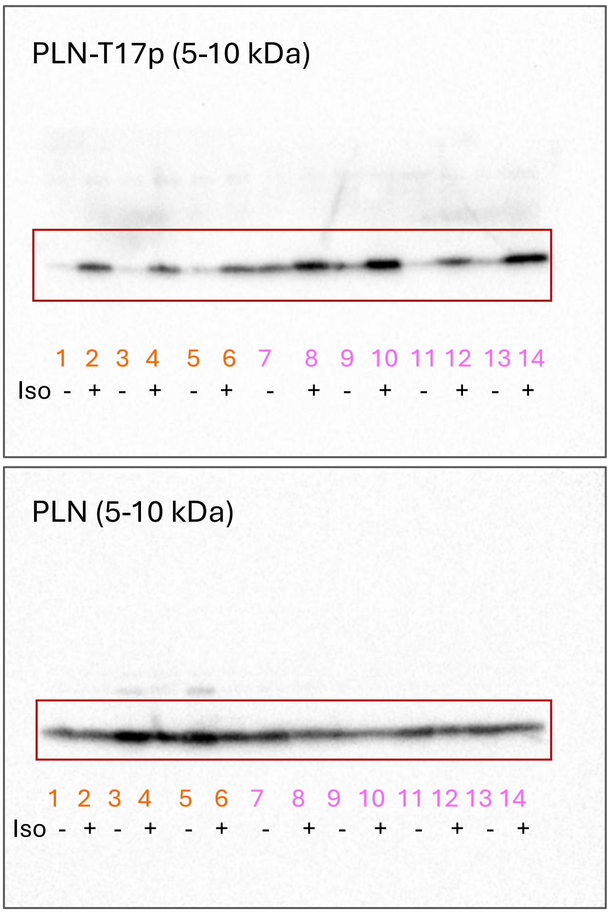


7 8 9 10 11 12 13 14 = LVNC 1 2 3 4 5 6 = resLVNC

Iso = Isoprenaline (1 µM; 15 min)

Basal samples also contributed to graph 6 d/e respectively

Used in analysis

# Original Western blot membranes; Rebs et al., 2026

### Fig. 6 d

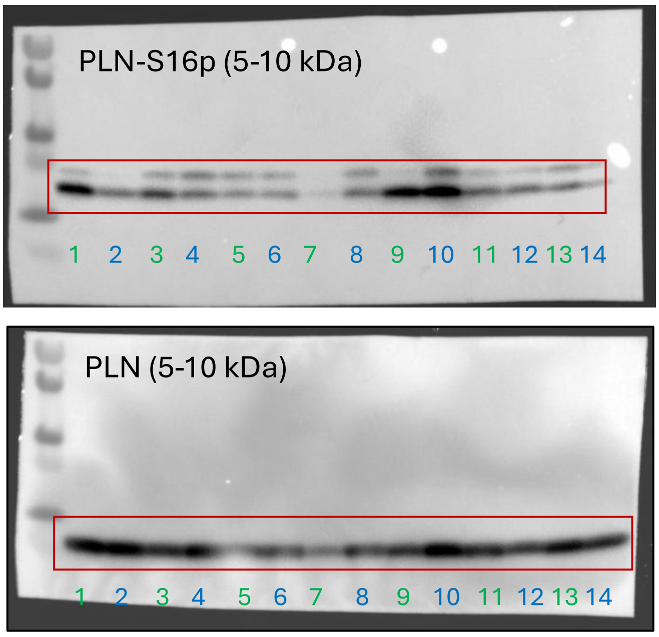


### Fig. 6 e

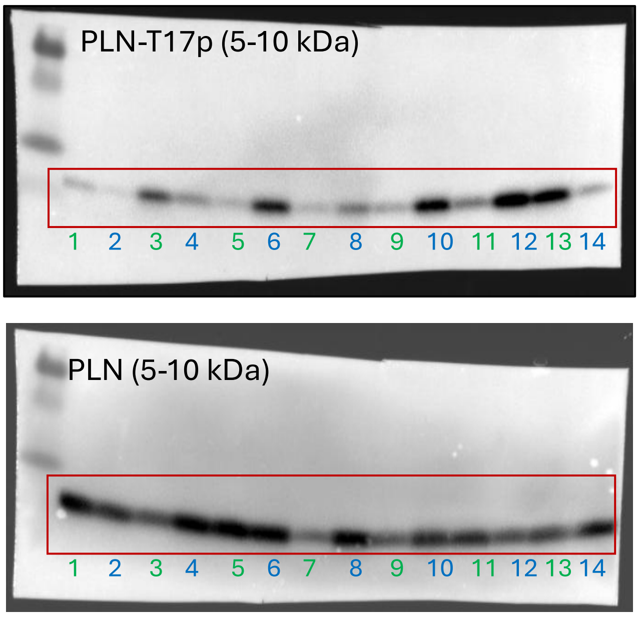


1 3 5 7 9 11 13 = resDCM 2 4 6 8 10 12 14 = DCM1

---

### Fig. 6 d/f

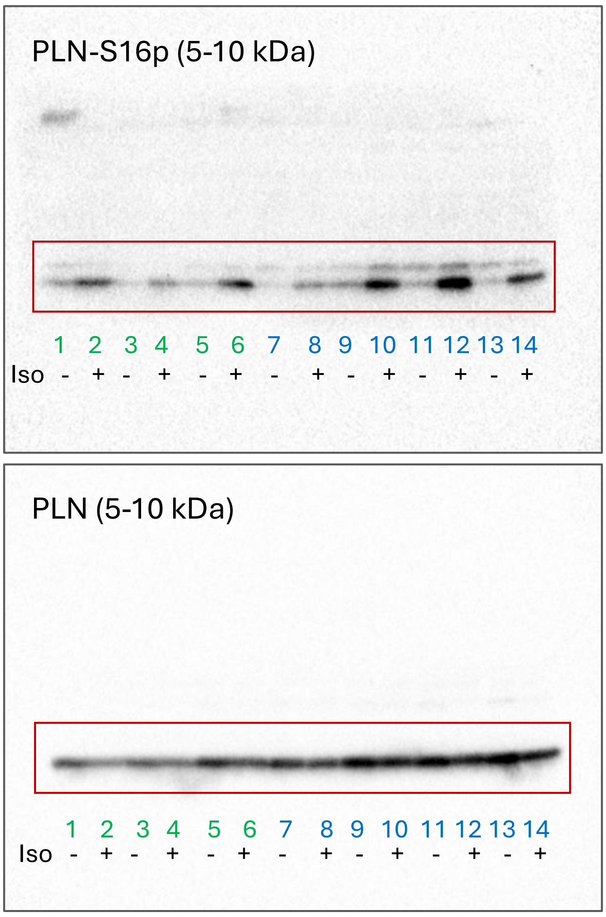


### Fig. 6 e/g

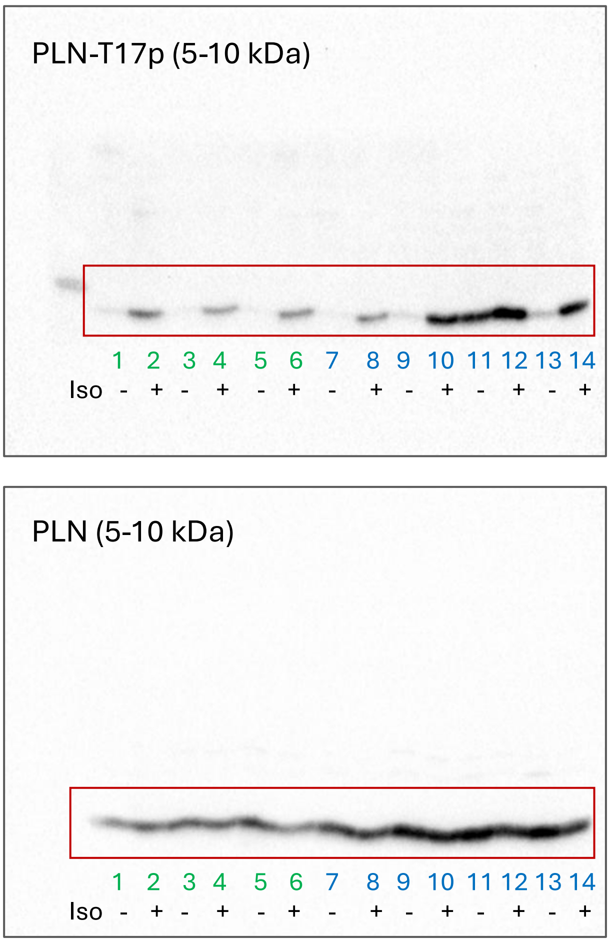


1 2 3 4 5 6 = resDCM 7 8 9 10 11 12 13 14 = DCM1+2

Iso = Isoprenaline (1 µM; 15 min)

Basal samples also contributed to graph 6 d/e respectively

Used in analysis
